# Spatiotemporal Mineral Phase Evolution and Arsenic Retention in Microfluidic Models of Zerovalent Iron-Based Water Treatment

**DOI:** 10.1021/acs.est.2c02189

**Published:** 2022-09-12

**Authors:** Jonas Wielinski, Joaquin Jimenez-Martinez, Jörg Göttlicher, Ralph Steininger, Stefan Mangold, Stephan J. Hug, Michael Berg, Andreas Voegelin

**Affiliations:** † Eawag, Swiss Federal Institute of Aquatic Science and Technology, 8600 Dübendorf, Switzerland; ‡ Department of Civil, Environmental and Geomatic Engineering, ETH Zürich, 8092 Zürich, Switzerland; § Institute for Photon Science and Synchrotron Radiation, Karlsruhe Institute of Technology, Hermann-von-Helmholtz-Platz 1, Eggenstein-Leopoldshafen, 76344 Karlsruhe, Germany

**Keywords:** arsenic, zerovalent iron, corrosion, water filter, microfluidics, microscopy, synchrotron X-ray spectroscopy

## Abstract

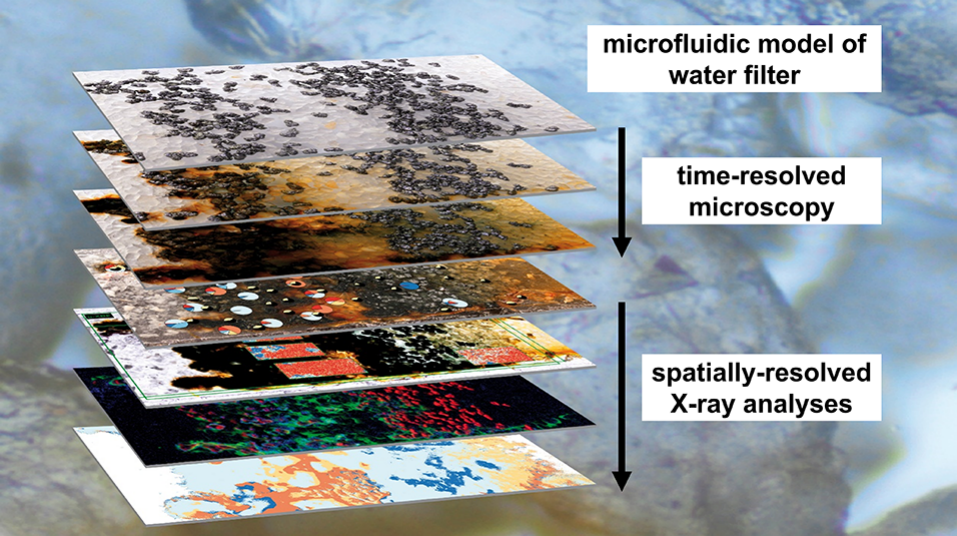

Arsenic (As) is a toxic element, and elevated levels of geogenic As in drinking water pose a threat to the health of several hundred million people worldwide. In this study, we used microfluidics in combination with optical microscopy and X-ray spectroscopy to investigate zerovalent iron (ZVI) corrosion, secondary iron (Fe) phase formation, and As retention processes at the pore scale in ZVI-based water treatment filters. Two 250 μm thick microchannels filled with single ZVI and quartz grain layers were operated intermittently (12 h flow/12 h no-flow) with synthetic groundwater (pH 7.5; 570 μg/L As(III)) over 13 and 49 days. Initially, lepidocrocite (Lp) and carbonate green rust (GRC) were the dominant secondary Fe-phases and underwent cyclic transformation. During no-flow, lepidocrocite partially transformed into GRC and small fractions of magnetite, kinetically limited by Fe(II) diffusion or by decreasing corrosion rates. When flow resumed, GRC rapidly and nearly completely transformed back into lepidocrocite. Longer filter operation combined with a prolonged no-flow period accelerated magnetite formation. Phosphate adsorption onto Fe-phases allowed for downstream calcium carbonate precipitation and, consequently, accelerated anoxic ZVI corrosion. Arsenic was retained on Fe-coated quartz grains and in zones of cyclic Lp-GRC transformation. Our results suggest that intermittent filter operation leads to denser secondary Fe-solids and thereby ensures prolonged filter performance.

## Abstract 2

Microfluidic studies combining spatiotemporal microscopy observations with X-ray spectroscopy data provide new insights into complex geochemical systems for water treatment.

## Introduction

In porous media such as aquifers, permeable reactive barriers, or water filters, geochemical processes at the mineral–water interface, including the formation, transformation, and dissolution of solids and colloidal transport, determine the retention of trace elements.^1^ Reactions at the mineral–water interface have been extensively studied in laboratory experiments using mineral suspensions, providing insights into surface chemical processes from the molecular and microscopic to the macroscopic level.^2^ Over the last decades, the understanding of surface reactions has also been substantially advanced through the use of synchrotron-based X-ray absorption spectroscopies.^2,3^ In porous media, however, the formation, transformation, and dissolution of solids and sorption processes are tightly coupled to water flow and the diffusive and advective transport of reactants in the pore space, which may lead to complex system behavior that differs from observations in suspensions.^1,4^ Spatially resolved information on the distribution and speciation of elements at the micrometer-scale is therefore crucial to further advance our understanding of mineral–water interface reactions in porous media. To date, however, spatially resolved studies have mostly focused on embedded samples and did not observe temporal evolution. Experiments in microfluidic flow channels (microchannels), coupled with optical microscopy and synchrotron X-ray spectroscopy, may allow us to bridge the gap between spatially and temporally resolved process-oriented geochemical studies in complex porous media.^4−9^ Although microchannels may not replicate the entire complexity of natural or engineered systems, their specifically reduced complexity allows us to disentangle the coupling between processes such as fluid–fluid and fluid–solid reactions under single and multiphase flow conditions.^4,7,10,11^ Microchannels are therefore well-suited for pore-scale investigations into phase formation and transformation in porous media.

The corrosion of zerovalent iron (ZVI) and secondary iron (Fe)-precipitation in drinking water filters for arsenic (As) removal is an example of a process that is highly dependent on pore-scale geochemical processes.^12−21^ Geogenic As in drinking water poses a threat to human health, and low-tech filters that make use of Fe corrosion are used for As removal in some low-income countries (see Supporting Information (SI) for more details).^16,22^ These filters contain metallic Fe (here denoted ZVI), which corrodes under oxic (eq 1) and anoxic conditions (eq 2) to form hydrous ferric oxides (HFO; here denoted as Fe(OH)_3_; eq 3) or carbonate green rust (GRC; Fe_6_(OH)_12_CO_3_ × 3H_2_O; eq 4) that provide sorption sites for As and other oxyanions.^13,14,16,23−26^
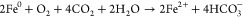
1
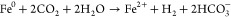
2
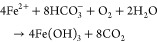
3
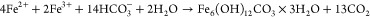
4

Longer-term filter operation performance has been proposed to benefit from periodic flow interruptions, possibly due to the formation of higher-density mixed valence magnetite (Fe_3_O_4_, ρ = 5.2 g/cm^3^) from HFO in the absence of dissolved oxygen (DO) (eq 5).^14^
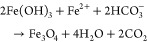
5

Magnetite formation reduces the volume of the precipitates in the pore-space and thus may counteract the loss of hydraulic conductivity over time.^14,25,27−29^ Moreover, magnetite binds both As(V) and As(III) through specific uptake mechanisms^30−32^ and its formation reduces ZVI passivation through increased electrical conduction compared to HFO.^33,34^

Here, we present the use of microchannels as models for pore-scale geochemical studies. The main goal was to improve the mechanistic understanding of the coupling of chemical and transport processes in ZVI-based filters for water treatment. For this purpose, we used (i) optical microscopy to follow the precipitation and transformation of Fe-solids in the microchannels by exploiting variations in the color of different types of Fe-minerals, (ii) synchrotron X-ray spectroscopy to determine the spatial distribution of elements, different types of Fe-solids, and As speciation after the experiments, and (iii) elemental analysis to quantify adsorption and precipitation in the microchannels. With this novel experimental setup, we gain insights into the formation and transformation of Fe phases in a ZVI/quartz matrix with spatiotemporal resolution, assess the interdependence of multiple co-occurring processes, and identify the effect of the cycling between periods of water flow and stagnation (no-flow) on ZVI corrosion, secondary Fe-phase formation, and As retention processes.

## Materials and Methods

### Preparation and Experimental Setup of Two Microchannels (Models A and B)

**Figure 1:**
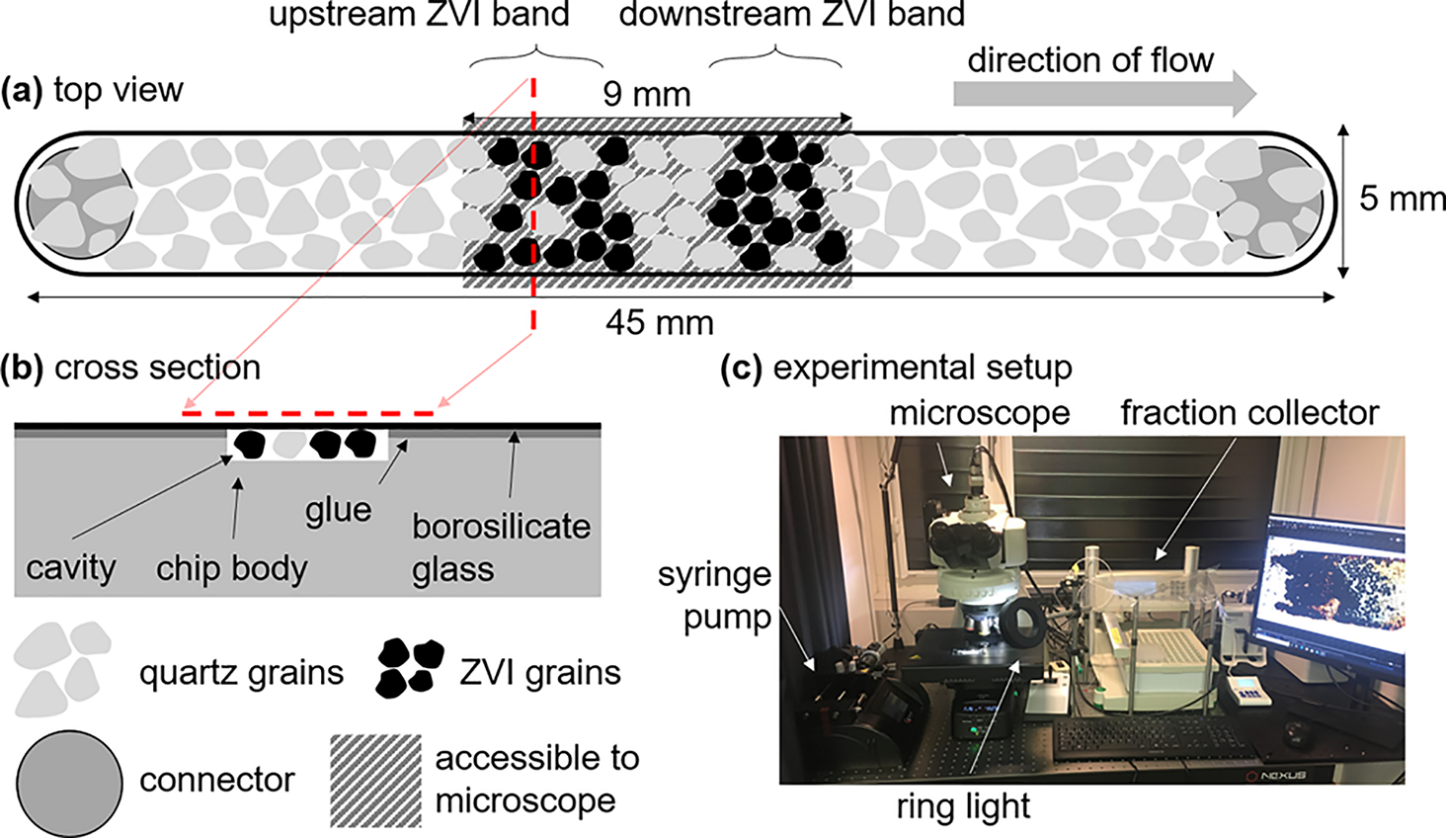
(a) Schematic depiction of the microflow channels (same for Models A and B) including the quartz grains (grey shapes), ZVI grains (black shapes), connectors (grey circles), and the area accessible to the microscope (striped area). Vertical, dashed, and red line indicates a cross-sectional cut, which is shown in (b). Channel is 45 mm long and 5 mm wide. Cavity is 250 μm deep and covered by a 170 ± 5 μm borosilicate glass (see b). Depiction is not true to scale. (c) Photograph of the experimental setup including the microscope, fraction collector, syringe pump, and ring light.

Two microchannels containing the Models A and B were prepared from gas-impermeable microfluidic flow-channels (45 mm long, 5 mm wide, 250 μm deep) covered by borosilicate glass (170 ± 5 μm) (μ-Slide, Luer Glass Bottom, Cat. No: 80167, ibidi GmbH, Gräfelfing, Germany). The channels were fitted with a Luer connector female on each end (Figure 1). Two Luer connectors male were filled with cotton to prevent gas bubbles from entering the channels. One of the connectors was plugged to the channel. Quartz grains (CAS #: 14808-60-7, Sigma-Aldrich, sieved to *d* = 200–250 μm) were added through the remaining opening, moved through the channel by gentle tapping and compacted using compressed air. Thereafter, ZVI grains (CAS #: 7439-89-6, 99.8+% purity, Table S1, American Elements, sieved to *d* = 200–250 μm) were added in the same manner. Another band of quartz and ZVI and more quartz were added until the gap was filled (Figure 1a). The two identical microchannels (Models A and B; Table S2) were used for subsequently different measurements. The pore volume was calculated from the channel volume and the masses and densities of added ZVI and quartz. Synthetic groundwater was supplied at a flow rate of 300 μL/h using a 50 mL glass syringe (1000 series Syringe, gastight, Hamilton) and a syringe pump (PHD Ultra, Harvard Apparatus). Based on the microchannel cross section, porosity, and volumetric flow rate, a water flow velocity of ∼2.2 mm/min (3.7 × 10^–5^ m/s) was estimated, which compares to pore velocities in ZVI-containing water filters in practice (2.8 mm/min^14^ and 2.1–4.2 mm/min).^35^ With respect to the transferability of the results from this study to field-scale filters, however, it should be noted that the thickness of the reactive layer in the microchannels extended only for a few millimeters, which is much shorter compared to ∼10 cm in field scale filters^14^ and that the micromodels did not include the secondary aeration step after ZVI passage that allows for further Fe(II) and As(III) oxidation and precipitation in commercial filters.

A silicon tube (0.5 mm inner diameter; ibidi GmbH) was used to connect the syringe to the microchannels. The microchannels were placed onto an automated stage (TANGO 3 Desktop, Märzhäuser, Germany) for positioning along three axes (*XYZ*) that were connected to an optical microscope operated in upright mode (Nikon Ni-E). With this setup, the length of image acquisition along the direction of water flow *(Y*-axis) extended over 9 mm (Figure 1a). A microscopy ring light (Schott EasyLED ringlight) was used to improve and ensure constant illumination and placed at a 45° (Model A) or 0° (Model B) angle relative to the channels to avoid reflection artifacts at the interface between the glass cover and the quartz/ZVI grains (Figure 1c). For Model A, a 0.5 mm inner diameter silicone tube was used to connect the outflow to a fraction collector (BioFrac, BioRad) equipped with a diverter valve and a micro-drop head (∼25 μL per drop) (total dead volume after channel outlet: 240 μL). Over each flow period, 12 fractions were collected. For Model B, one integrated sample was collected per flow period using a fraction collector (LKB Bromma 2212 Helirac).

The experimental runtimes of Models A and B were 13 and 49 days, respectively. Model B was in constant no-flow mode between days 37 and 42 to investigate the effect of the prolonged absence of water flow. Therefore, only 44 flow/no-flow cycles were run on Model B in total. At the end of the experimental runtime, the water in the channels was exchanged for an epoxy resin (EpoFix, Struers), and the glass cover was removed for X-ray analyses (details in SI).

### Synthetic Groundwater and Chemical Analysis

Synthetic groundwater contained sodium (Na; 73.4 mg/L, 3.2 mM), calcium (Ca; 62.5 mg/L, 1.6 mM), silicate (Si; 16.4 mg/L SiO_4_-Si, 0.59 mM), phosphate (P; 3.5 mg/L PO_4_-P, 0.11 mM), arsenite (As(III) 570 μg/L, 7.6 μM), and bicarbonate (HCO_3_^–^; 299 mg/L, 4.9 mM) dissolved with excess CO_2_. The solution was purged with air to adjust the pH to 7.5 and was thus saturated with O_2_ in air (∼8 mg/L dissolved oxygen (DO)). The resulting CO_2_ partial pressure was ∼10–12 mbar CO_2_ (calculated using Visual Mitec 3.1, see the SI).^36^ Element concentrations in the influent and effluent water were determined by inductively coupled plasma mass spectrometry (ICP-MS; Agilent 8900QQQ; Table S3). Fe, Na, Si, and Ca were measured on-mass in helium collision mode, P and As were measured by MS/MS in mass shift mode after reaction with O_2_. For Model B, the redox speciation of dissolved As was analyzed by high-performance liquid chromatography (HPLC) coupled to ICP-MS and shown to be dominated by As(III) (see the SI).

The effluent fractions from Model A were collected into 96-well plates. Because of the very small flow rate and sample volumes, partial evaporation occurred during sampling. Therefore, the 96-well plates were placed in a desiccator to dry, and the precipitates in each vial were redissolved in 250 μL of 1% (w/w) hydrochloric acid (HCl). Thereafter, 200 μL of the acid extract was diluted in 5 mL of 1% nitric acid (HNO_3_) to determine the total concentrations of Na, Si, P, Ca, Fe, and As by ICP-MS. The effluent of Model B was collected over longer time spans, immediately stabilized by the addition of HCl to reach 1% HCl and stored in the dark at 3 °C. The stabilized fractions were diluted in 1% HNO_3_ for the determination of total concentrations by ICP-MS and in doubly deionized (DDI) water for As speciation by HPLC-ICP-MS. Drying and resuspending induced complete As(III) oxidation; therefore, no As speciation could be measured for the effluent of Model A. Measured element concentrations were normalized to the measured influent concentration of Na (recovery Model A: 68 ± 11%; Model B: 92 ± 11%) to account for sampling losses.

### Optical Microscopy Measurements and Image Analysis

The automated stage, optical microscope, and CCD camera (DS-Fi3, Nikon) were controlled by the Advanced Research software code (Nikon). An area of 9 × 5.5 mm^2^ was recorded with a 0.8 μm/pixel resolution and stitched from multiple images with 10% overlap. Each individual image was autofocused by selecting focus from ten 20 μm steps through the focal plane. The exposure time was 20 ms, and no analog amplification was applied. An entire image was recorded every 30 min, exported in Tagged Image File Format (TIFF) and subsequently imported into Matlab for analysis and compression into MPG4 files. Size-reduced movies of the Models A and B from microscopy images are available in the Web Enhanced Objects Videos 1 and 2, respectively. Annotations are explained in the text. Model A was monitored with the microscope over the entire experimental runtime (13 days) and Model B for 5 days (day 44–49). During day 1–42, Model B was monitored using a CCD camera (ThorLabs), and a movie is included in Video 3. Repetitive color changes were observed in Model A, and a threshold was applied for the quantitative binary description of these color changes (Video 4). The change of bright to dark phases and vice versa between the two operational modes (flow/no-flow) in Model A in comparison to the As distribution is shown in Video 5.

### Synchrotron X-ray Spectroscopy

Micro-focus (μ-) and full field synchrotron X-ray spectroscopy measurements were performed at the SUL-X and XAS beamlines at the KIT Light Source (Karlsruhe Institute of Technology; Germany). At the SUL-X beamline, the spatial distributions of Fe, Ca, P, and As were determined by μ-X-ray fluorescence spectrometry (μ-XRF; resolution 40 × 40 μm^2^) (Models A and B), the speciation of Fe and As in selected points by μ-X-ray absorption spectroscopy (μ-XAS; spot size 50 × 50 μm^2^) (Models A and B). The Fe *K*-edge X-ray absorption near-edge structure (XANES) and extended X-ray absorption fine structure (EXAFS) spectra were evaluated by linear combination fitting (LCF) using six reference spectra: metallic Fe, vivianite (Viv), carbonate green rust (GRC), magnetite (Mag), lepidocrocite with a low degree of crystallinity (Lp), and amorphous Fe-phosphate precipitated in the Ca-containing background electrolyte (CaFeP). The Mag and metallic Fe spectra were recorded at the SUL-X beamline. A Viv spectrum recorded at the XAS beamline was kindly provided by Mingkai Ma (Utrecht University, The Netherlands), spectra of Lp and CaFeP were available from own earlier work,^37^ and the spectrum of GRC (from ref (21)) was kindly provided by Case van Genuchten (Geological Survey of Denmark and Greenland). The evaluation of the As K-edge μ-XANES spectra by LCF was based on the reference spectra of As(III) and As(V) adsorbed onto ferrihydrite (Fh) (As(III)-Fh or As(V)-Fh; recorded at the SUL-X beamline; from ref (38); kindly provided by Hongyang Wang (KIT, Germany)) and of As(V) incorporated in Mag (As(V)-Mag; from ref (39); kindly provided by Case van Genuchten).

The spatial Fe phase distribution in Model A was probed by chemical imaging at SUL-X and by full field transmission XANES spectroscopy at the XAS beamline. For chemical imaging, μ-XRF maps (resolution 40 × 40 μm^2^) were recorded on four selected areas at 20 energies across the Fe *K*-edge, and the resulting XANES maps were evaluated by LCF using the Fe *K*-edge reference spectra listed above. The full field Fe XANES dataset consisted of ∼2.2 × 10^6^ spectra recorded on an area of ca. 12 × 4.5 mm^2^ with 5 × 5 μm^2^ pixel resolution. This dataset was evaluated by principal component and cluster analysis.^40,41^ Details on the synchrotron data acquisition and evaluation are available in the SI.

## Results and Discussion

### Effluent Concentrations and Dissolved as Speciation

**Figure 2:**
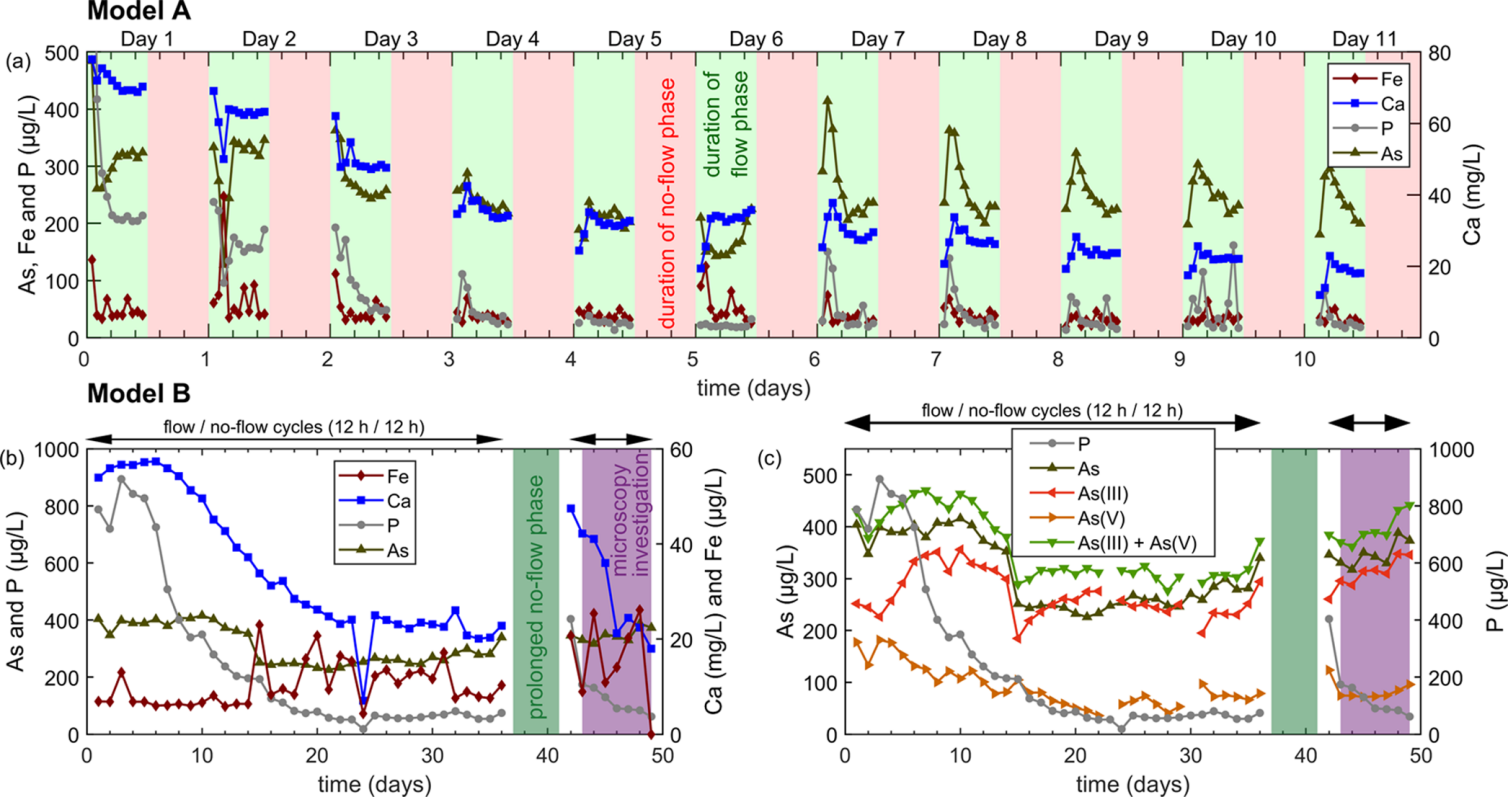
Models A and B. (a) Effluent concentrations of Fe, P, and As (left axis) and Ca (right axis) for the first 11 days of Model A operation. (b) Effluent concentrations of As and P (left axis) and Ca and Fe (right axis) of Model B. (c) Arsenic redox speciation and total As and P in the effluent of Model B. Shaded areas (a) indicate the flow (green)/no flow (red) phases in Model A. Shaded areas in (b) and (c) indicate the prolonged no-flow period in Model B (dark green) and the period investigated by optical microscopy (violet). Horizontal arrows indicate when Model B was operated with 12 h/12 h flow/no-flow cycles. Concentrations of Fe, Ca, P, and As were normalized to the recovery of Na (Model A: 68 ± 11%; Model B: 92 ± 11%) in the analyzed fraction. Blank values indicate missing data.

In Model A, effluent Fe concentrations were mostly constant, whereas As, Ca, and P decreased over time (Figure 2a). In total, ∼1.6 μg Fe was released with the effluent, more than two orders of magnitude less than the theoretical maximum of 0.74 mg of corroded Fe that would result from complete oxic corrosion (eq 1) and oxidation of Fe(II) (eq 3) during days 1–11 (Table S4). The Ca concentration during the first flow period (60 mg/L) was close to the influent concentration but progressively decreased over subsequent days. The P concentration was 1.2 mg/L in the first fraction (∼30% of the influent P), but after 2 days, it was mostly below 100 μg/L. The As concentration decreased to ∼200 μg/L (∼40% of influent concentration) over the first 6 days. From day 7 to 11, a marked spike in effluent As concentration was observed whenever water flow was resumed. A comparable trend of initially increasing effluent concentrations during each flow period was observed for Ca and partially P. Although the As concentration was as low as 130 μg/L in some fractions, it varied mostly between 200 and 300 μg/L. The results indicated that retention of As, P, and Ca occurred in Model A and that the available porous medium and fluid residence time downstream the ZVI bands or supply of DO was insufficient to precipitate all Fe released through ZVI corrosion.

In Model B, the Fe concentration varied between 5 and 30 μg/L with stronger variations after the prolonged no-flow period (days 37–42, Figure 2b). Calcium varied between 40 and 60 mg/L in the first 10 days but stabilized at around 25 mg/L after 20 days. After the prolonged no-flow period, Ca in the effluent increased to up to 50 mg/L but decreased again over the remaining 5 days. Phosphorous was at 800 μg/L during day 1–5 but then rapidly decreased to below 100 μg/L. The prolonged no-flow period resulted in an increased effluent P concentration during one day before the P concentration decreased again to the level before the extended interruption. The As concentration was initially at 400 μg/L, decreased to 240 μg/L after 15 days, and then increased progressively until reaching almost the initial values after 50 days. The prolonged no-flow period did not affect the effluent As concentration. Effluent As initially consisted of similar fractions of As(III) and As(V) but was increasingly dominated by As(III) over time (Figure 2c), which accounted for 90% of the total As after 22 days. This indicated that insufficient DO supply increasingly limited As(III) oxidation. Dissolved As(V) and P concomitantly decreased (days 1–5), which indicated that an increasing number of P and As(V) sorption sites became available. In total, 23 μg of As was introduced into Model A over 11 days, of which ∼60% were retained. In Model B, 90 μg of As was introduced over 45 days, and ∼44% were retained, reflecting the decreasing As retention over operation time.

### Color Cycles and Dynamics of Particles and Gas Bubbles from Time-Resolved Optical Microscopy

**Figure 3:**
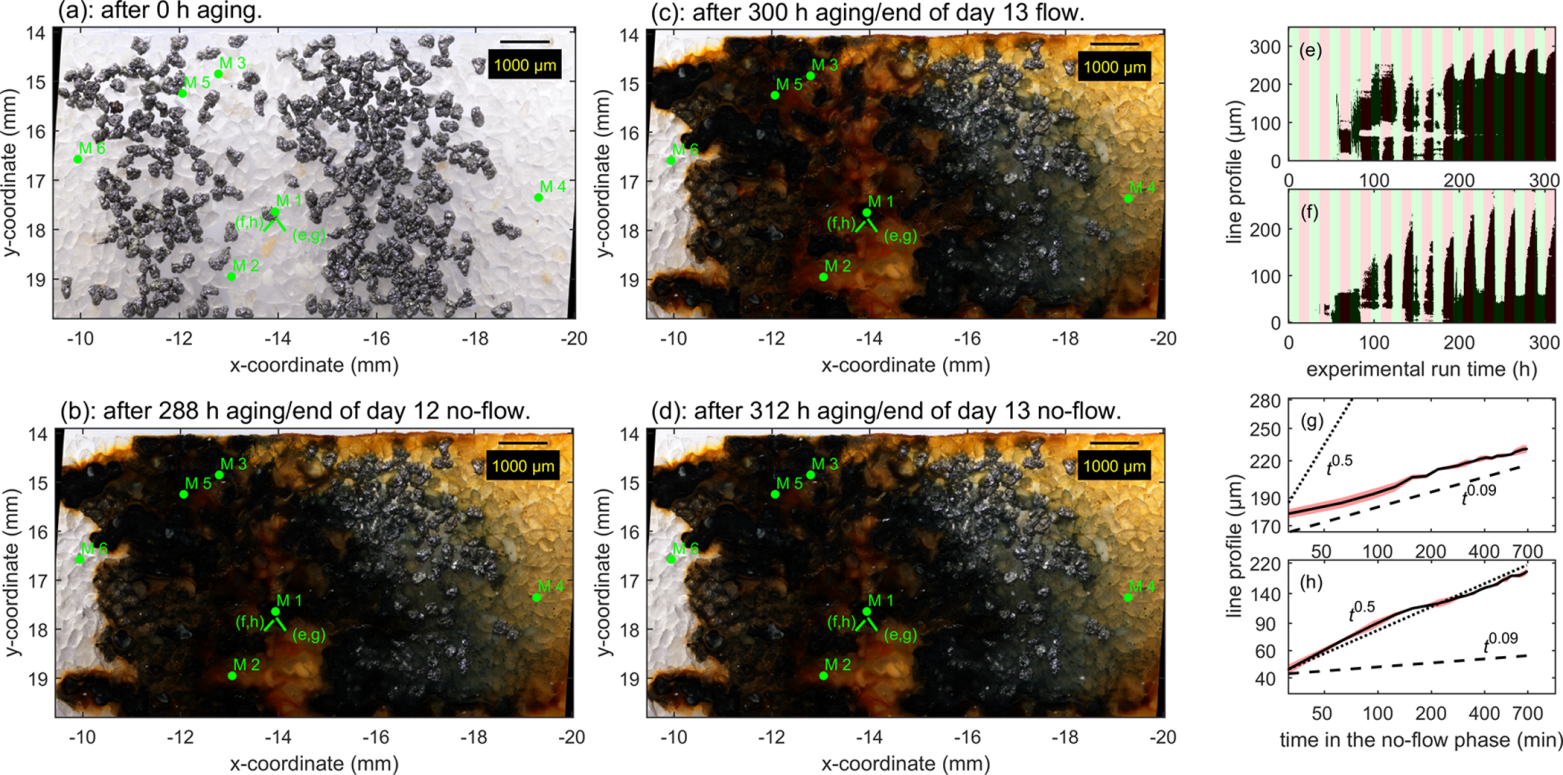
Model A. Optical microscopy images of Model A at 0, 288, 300, and 312 h of aging (a–d; see Video 1). Green markers (M1–M6) indicate locations at which redox cycling, particle transport, and gas bubbles were observed. Details are discussed in the text. Short, green bars indicate the orientation of line profiles presented in which repeated cycling between the bright anddark phase was observed (corresponds to the last frame inVideo 5). Green color indicates the distribution of As. (f) Full field Fe XANES results (colors correspond to color bar in (b); small rectangles indicate locations of chemical images in (a); white areas too low in Fe). Images (a, b) without annotations and individual element distribution maps (Fe, As, P, and Ca) are displayed in dotted lines indicate slopes of 0.09 and 0.5, the latter indicative of diffusion-limited reaction (see text).

At the beginning of the experiments, all Fe was contained in ZVI grains (Figure 3a). The contact with water initiated the corrosion of the ZVI and the precipitation of secondary Fe-phases (Figure 3b–d and Video 1). In this section, we discuss the color evolution of secondary Fe-phases over repeated oxidation–reduction cycles induced by water flow and no-flow periods, the formation and transport of colloidal particles, and the formation and behavior of gas bubbles in Model A as observed by time-resolved optical microscopy.

#### Color Cycles

After a 5-day initiation phase in which secondary Fe-solids began to accumulate, a pattern of cyclic color changes over-flow/no-flow periods evolved (25 s, Video 1). During no-flow, Fe-solids around ZVI grains turned black, with dark patches growing away from ZVI grains. Conversely, upon re-initiation of water flow, the dark patches rapidly retreated again to the vicinity of the ZVI grains. These zones of cyclic color change (labeled with markers M1 and M2, Video 1) were most prominent during the last 8 cycles (Video 4) and slowly expanded away from the ZVI grains. This is exemplarily shown in Figure 3b–d: at the end of the no-flow period of day 12, dark colors dominated around reacted ZVI (e.g., M1 and M2, Figure 3b), after the subsequent 12 h of flow, bright colors dominated the reaction zone (Figure 3c), and after another 12 h of no-flow, the dark color reappeared (Figure 3d).

Oxidized Fe-phases often assume bright, red (ferrihydrite (Fh), hematite)/orange (lepidocrocite) colors, mixed-valence Fe-phases dark green/blue (GRC)/brown/black (magnetite) colors. Therefore, the cyclic color changes indicated (partial) Fe-phase transformations, and the spatial relation to the corroding ZVI suggested that dissolved Fe(II) released by ZVI corrosion was involved in the observed reaction. For a semiquantitative analysis, two line profiles were drawn across expanding/shrinking zones (short, green bars Figure 3a–d), and the extension of the dark phase as a function of time was extracted (red shaded areas Figure 3e, f and Video 4). The mean and standard deviation of the extension of the dark phase away from the ZVI grain after no-flow initiation in the last four cycles (day 9–13, red shaded areas Figure 3e, f) were calculated and displayed on a double logarithmic scale (Figure 3g, h). The first line profile increased with a slope of 0.09 (dashed line, *R*^2^ = 0.9934, Figure 3g), the second with a slope of 0.5 (dotted line, *R*^2^ = 0.9923, Figure 3h). Diffusion-limited reactions exhibit a slope of 0.5 (dotted line, Figure 3f), resulting from the root mean squared displacement of molecules over a diffusion time σ *=* (2*Dt*)^0.5^, where *σ* is the distance and *D* is the diffusion coefficient. Therefore, the slope of the first line profile suggested that the bright to dark transformation was limited by Fe(II) availability, whereas the transformation along the second line profile appeared diffusion-limited. This anisotropic behavior probably resulted from decreasing ZVI corrosion as ZVI grains became inhomogenously passivated, DO was locally depleted (eq 1), and the pH locally increased (eq 2). The minimal extension of the dark phase from the ZVI grain increased 20 μm over 5 cycles (Figure 3e). In contrast to the expansion of the dark phase, the dark-to-bright transformation upon flow resumption was completed rapidly (green shaded areas, Figure 3e, f), indicating rapid oxidation initiated by the advective transport of DO into the reaction zones. Note that this data interpretation treated the microchannel as a two-dimensional lateral (*XY*) system and ignored its vertical (*Z*) extension, which may have introduced some bias into the analysis. In Model B, no redox cycling comparable to Model A was observed at a later stage of operation after a prolonged no-flow period, and only minimal color differences between flow and no-flow were observed by optical microscopy (Video 2).

#### Particle Formation and Transport

Fe precipitates initially formed on the surfaces of ZVI and neighboring quartz grains during the flow period (M3, Video 1). During flow, the initially green fresh precipitates (GRC) turned bright orange (Lp). During the following no-flow period, the precipitates gradually darkened. After a complete 24 h flow/no-flow cycle (5 s, Video 1) and re-initiation of the flow, dark particles (*d* = 10–15 μm) could be observed on bright quartz grains. Downstream of both ZVI bands, many green particles up to *d* = 30 μm were formed after the first 24 h cycle (M4, Video 1). Upon flow re-initiation, particles were partly detached and transported downstream for about 3 h (∼40 pore volumes) before they re-attached until the next flow re-initiation. During the first 3 h after flow resumption, the Ca concentration increased, e.g., from 19 to 33 mg/L during day 6 (Figure 2a). In this range, increasing concentrations of divalent cations strongly increase colloid deposition rates.^42^ Therefore, particle deposition was potentially influenced by temporal variations in Ca precipitation.

#### Gas Bubbles

Gas bubbles repeatedly appeared upstream or in the ZVI bands during flow (M5 and M6, Video 1). Some of these bubbles formed prior to entering the field of view. Gas bubbles that became trapped upstream the ZVI bands underwent repetitive growth during flow and shrinkage during no-flow (e.g., M6, Video 1). Considering that the inflow was equilibrated with 10–12 mbar CO_2_, we assume that these bubbles consisted of air and CO_2_ and that their growth during flow was largely due to CO_2_ outgassing, while their disappearance during no-flow was due to CO_2_ dissolution.

Within the upstream ZVI band, small, dark particles as described above accumulated at gas-water interfaces (e.g., M5, Video 1). This type of particle accumulation has previously been observed^7^ and can significantly enhance colloidal transport in unsaturated porous media in case of multiphase flow.^43^ Once gas bubbles in contact with ZVI grains started to shrink, they were dragging small precipitates from the surface of the solid grains downstream. Eventually, the dissolution of these gas bubbles led to the local aggregation of the micron-sized particles (5 s, M 5, Video 1). In Model B, gas bubbles mainly formed in the lower part of the downstream ZVI band (Video 3), indicative of the formation of H_2(g)_ during anoxic ZVI corrosion.^44−46^

### Element Distributions from X-ray Fluorescence Microspectrometry

**Figure 4:**
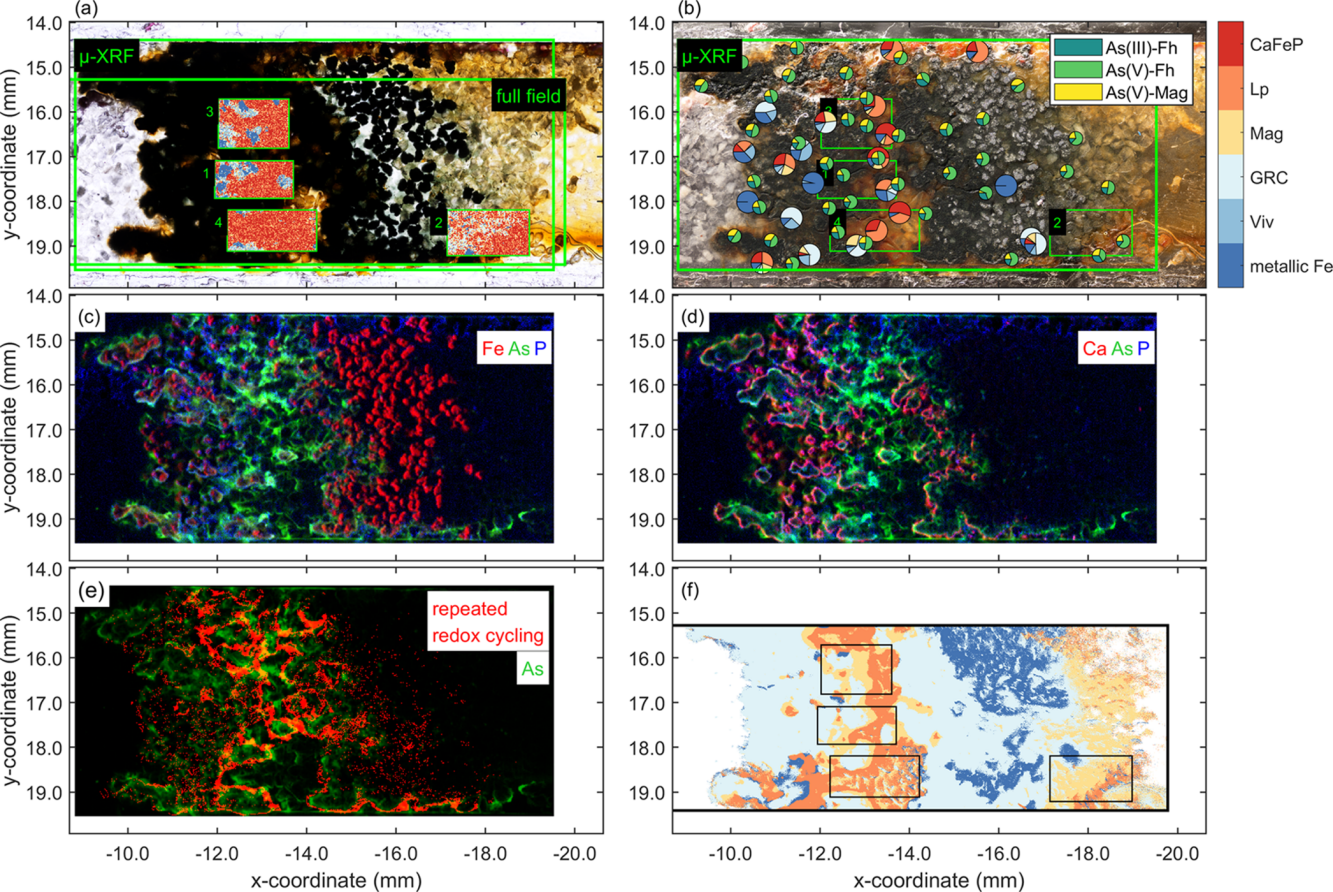
Model A. (a) Transmitted and (b) reflected light optical microscopy images of the resin-impregnated Model A after removal of the top glass. In (a), frames labeled “μ-XRF” and “full field” indicate areas probed by μ-XRF and full field Fe XANES. Insets labeled 1–4 show chemical imaging results for Fe (enlarged in Figures S9–S16; color bar legend in panel (b)). In (b), small pie charts indicate As μ-XAS speciation (As(III)-Fh in dark green, As(V)-Fh in light green and As(V)-Mag in yellow, see legend), large pie charts Fe μ-XAS speciation (references CaFeP, Lp, Mag, GRC, Viv, and metallic Fe; see the color bar) both derived from XANES LCF analysis. (c, d) Tri-color maps show the distribution of (c) Fe-As-P and (d) Ca-As-P. (e) Red color indicates locations in which repeated cycling between the bright and dark phase was observed (corresponds to the last frame in Video 5). Green color indicates the distribution of As. (f) Full field Fe XANES results (colors correspond to color bar in (b); small rectangles indicate locations of chemical images in (a); white areas too low in Fe). Images (a, b) without annotations and individual element distribution maps (Fe, As, P, and Ca) are displayed in Figure S1.

The resin-impregnated Model A was analyzed by transmitted and reflected light optical microscopy for visual inspection (Figure 4a, b) by μ-XRF to determine Fe, As, P, and Ca distributions (Figures 4c, d, e and S1), by Fe and As *K*-edge μ-XAS (pie charts, Figure 4b), to assess Fe and As speciation at selected points, by Fe *K*-edge chemical imaging (inserted frames, Figure 4a), and Fe *K*-edge full field XANES spectroscopy (Figure 4f) to assess the spatial distribution of Fe-phases over entire areas of interest.

In the transmitted light image, individual ZVI grains were visible against the bright background at *x* = −16 mm. The reflected light image showed that these grains had been truncated by sample polishing, which resulted in the exposure of the unreacted Fe metal surface. Higher Fe-fluorescence counts were recorded on these localized truncated ZVI grains than on partially corroded ZVI grains further upstream (Figure 4c), misleadingly indicating a strong Fe depletion of the upstream ZVI band. Because of the orientation of the sample (10° relative to the beam) and detector (90° relative to the beam), shading suppressed the emitted XRF on the right side of dense ZVI grains (Figure 4c), as confirmed by complementary data recorded on a laboratory μ-XRF instrument with dual detector configuration (Figure S2). In general, however, the Fe fluorescence data correctly reflected the more advanced ZVI corrosion and Fe-precipitate formation in the upstream compared to the downstream ZVI band, as also seen in the microscopy images.

Calcium was mostly located around reacted ZVI grains, frequently on the upstream side (Figure 4d). This was attributed to CaCO_3_ precipitation promoted by ZVI corrosion and resulting DO and H^+^ consumption.^45^ Phosphorous was mostly located in the upstream ZVI band (*x* = −13 to −10 mm, Figure 4c, d). Its retention was attributed to the strong binding of phosphate onto Fe-precipitates^47^ and to phosphate uptake into Ca phases that form at elevated pH around ZVI grains (as indicated by the colocalization of P with Ca, Figure 4d).^48^

Arsenic was mainly retained between the two ZVI bands (*x* = −14 to −12 mm, Figure 4c, d), downstream of P retention. This spatial separation of P and As removal can be attributed to the comparably weak adsorption of arsenite onto HFO (mainly uncharged H_3_As^III^O_3_, p*K*_a,1_ = 9.2) relative to phosphate (present as singly and doubly deprotonated oxyanion) and the limited interaction of arsenite with Ca. Downstream As retention was promoted by Fenton-catalyzed oxidation of weakly adsorbing arsenite to strongly adsorbing arsenate.^49^ In the center of the microchannel (*x* = −13.5 mm, *y* = 17 mm), As and Fe co-occur in Fe coatings around quartz grains (Figure 4c, d), in contrast to P or Ca, confirming that As is retained by adsorption onto Fe precipitates.

**Figure 5:**
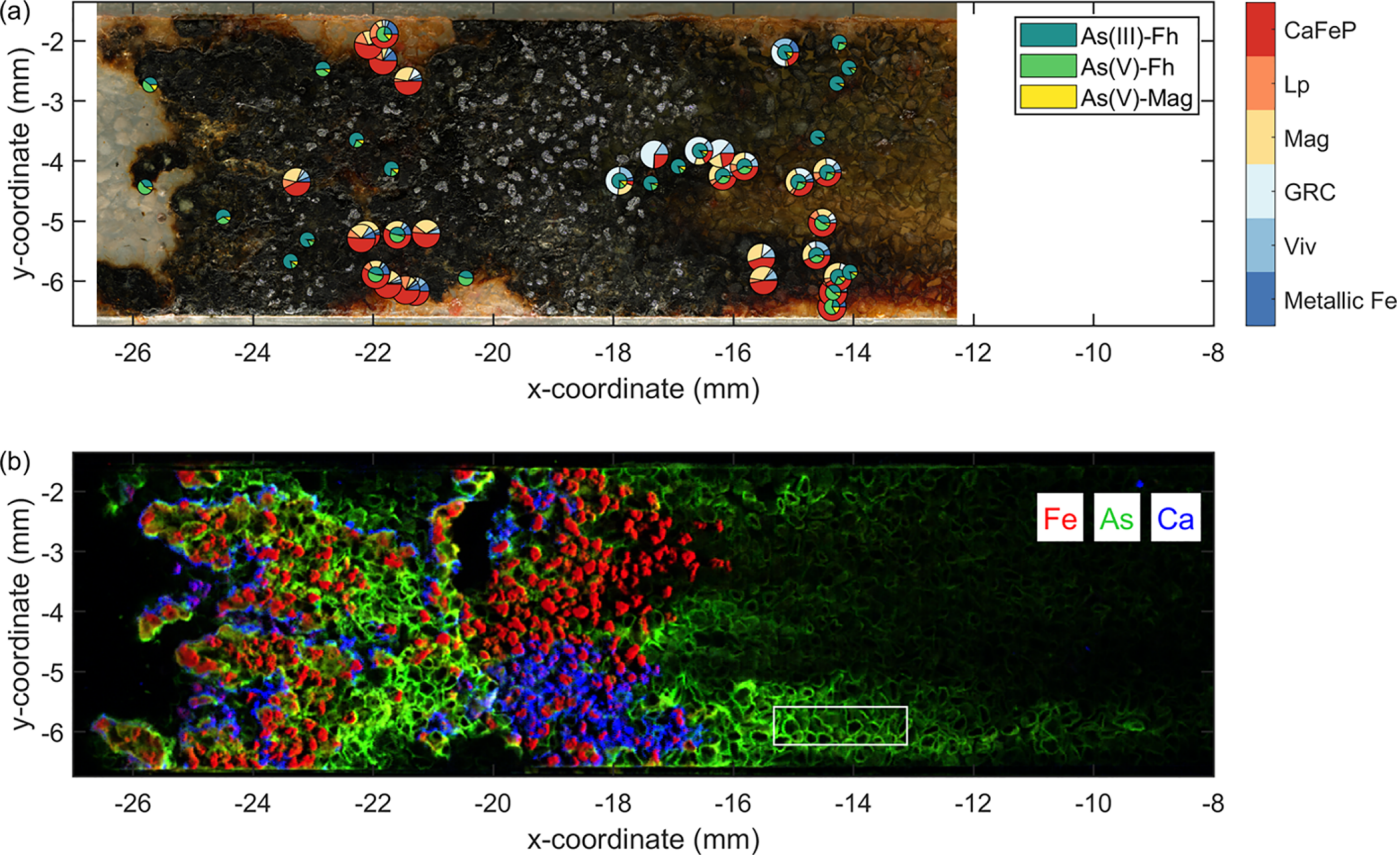
Model B. (a) Reflected light optical microscopy image of the resin-impregnated microchannel after removal of the top glass. Small pie charts indicate As μ-XAS speciation (As(III)-Fh in dark green, As(V)-Fh in light green, and As(V)-Mag in yellow; see legend), large pie charts Fe μ-XAS speciation (references CaFeP, Lp, Mag, GRC, Viv, and metallic Fe; see color bar) derived from XANES LCF analysis. (b) Tri-color distribution map for Fe, As and Ca. White frame indicates an area over which As–Fe correlation was assessed (Figure S5a). Black area around *x* = −20 and *y* = −3 to −2 corresponds to small sample fragment that broke off prior to synchrotron analyses.

For Model B, the reflected light image is shown together with Fe and As *K*-edge μ-XAS data (pie charts, Figure 5a); the μ-XRF data (Fe, As, Ca) are shown as a tri-color map (Figure 5b) or individually (Figure S3). Similar to Model A, higher Fe fluorescence intensities were observed in the less-corroded downstream ZVI band than the upstream band. Calcium precipitated at the front of the upstream ZVI band, locally inside this ZVI band (*x* = −26 to −22 mm, *y* = −4.25 mm) and in one large patch in the downstream ZVI band, most probably along a preferential flow path, where ZVI corrosion was most intense. The distribution map for P showed that P was mainly accumulated in the upstream ZVI band (Figure S4; data measured on a laboratory μ-XRF instrument for technical reasons). Like in Model A, As was mainly retained downstream of P, between the ZVI bands and downstream of the precipitated Ca. The close correlation between As and Fe downstream of the Ca patch (white frame Figures 5b and S5a) again showed that As (as in Model A) was mainly retained in Fe coatings on quartz grains (Figure S6e,h).

### Fe and As Speciation Distribution

The Fe speciation was determined using three complementary X-ray techniques: (i) μ-XAS (XANES and EXAFS) (Models A and B), (ii) chemical imaging (Model A), and (iii) full field XANES spectroscopy (Model A). Chemical imaging used the fluorescence signal, whereas full field XANES spectroscopy and μ-XAS used the transmission signal (except for one spectrum, for which the fluorescence signal was evaluated). Thus, chemical imaging was more sensitive to the surface speciation compared to the other two methods. The As speciation was determined by fluorescence μ-XAS (XANES). Data interpretation was based on LCF analysis using reference spectra.

#### Fe μ-XANES

On Model A, 22 Fe μ-XANES spectra were evaluated by LCF (large pie charts in Figure 4b, spectra in Figure S7a, LCF results in Table S5). In general, Lp dominated on points between the ZVI bands, and GRC dominated inside the ZVI bands. Significant fractions of Viv, Mag, and CaFeP were fitted in a limited number of locations, and three spectra recorded at the boundary of ZVI grains where the beam could still pass were dominated by metallic Fe.

On Model B, 31 Fe μ-XANES spectra were evaluated by LCF (Figures 5a, S8a, and Table S6). In the upstream ZVI band and between the two bands, the CaFeP reference dominated the LCF. GRC was the most important in the center (*y* = −4 mm) downstream the second ZVI band. Significant fractions of Mag were fitted at the interfaces between the ZVI bands, along a preferential flow path through the upstream ZVI band (*x* = −22 mm, *y* = −5 mm) and on the Fe-coated quartz grains.

#### Fe Chemical Imaging

Chemical images were derived from 20 μ-XRF stacks collected across the Fe *K*-edge in four selected areas, and each pixel was analyzed by LCF. The dominant fit component in each pixel is shown in the insets of Figure 4a (details in Figures S9–S16). In the center of the Fe precipitates around the reacted ZVI grains (frames labeled 1 and 3 Figure 4b), metallic Fe, Viv, and GRC dominated (insets 1 and 3 Figure 4a). Most distant from these locations, the oxidized phases (Lp, CaFeP) were prevalent. Although a coherent picture emerges, these pixels randomly indicated CaFeP or Lp because of the relative similarity of these reference spectra.^50^ At the red–blue interfaces, a thin zone of Mag was observed (e.g., Figure S9a, b).

#### Full Field XANES Spectroscopy

The full field data offered insights into Fe-phase distribution over a larger area. To treat the extensive amount of data (2.2 million spectra), principle component analysis (PCA) combined with cluster analysis was used to identify areas dominated by similar spectral features. This approach returned 4 areas referred to as metallic Fe-like, GRC-like, Mag-like, and Lp-like clusters (Figure 4f, color bar in Figure 4b, spectra in Figure S17). The spectrum representative of the metallic Fe-like cluster was characterized by high absorbance. Because the thickness of a 200-μm ZVI grain largely exceeds the X-ray adsorption length above the Fe *K*-edge (3.1 μm), corresponding zones on the fluorescence screen behind the sample should not have registered any transmitted X-ray signal. Nevertheless, pixels in such areas were retained for analysis by edge-step filtering and allocated to the metallic Fe cluster (Figure 4a, f). We speculate that the scattering of transmitted X-rays in the sample and the polymer support behind the sample caused a spectral signal in zones of the fluorescence screen blocked from direct irradiation and that this signal corresponded to the spectral signal of nearby zones, where the X-ray beam could pass. The spectrum of the GRC-like cluster also revealed spectral features of metallic Fe and Viv, the Mag-like cluster also contained spectral features of GRC and the Lp-like cluster also showed spectra features of CaFeP. This mixture of spectral components in the spectra of the GRC-, Mag-, and Lp-like clusters was attributed to the co-occurrence of multiple phases along the X-ray beam path through the sample. Despite all confounding factors, the compositional clusters identified by full field XANES analysis overall matched remarkably well with Fe *K*-edge μ-XANES data (Figure S17): the metallic Fe-like cluster was only observed in the downstream ZVI band, well correlated to the corrosion front visible by optical microscopy (Figure 4b, f); the GRC-like cluster was the most important in the upstream ZVI band and also prevalent in the downstream ZVI band (Figure 4f). The Lp-like cluster was mainly identified between the ZVI bands, and the Mag-like cluster was mostly located between the Lp- and GRC-like clusters.

#### As μ-XANES

On Model A, 40 As μ-XANES spectra were recorded and evaluated by LCF (small pie charts in Figure 4b, spectra in Figure S18, LCF results in Table S7). As redox speciation in most of the probed points was dominated by As(V) (sum of As(V)-Fh and As(V)-Mag; up to ∼87%), the largest fractions of As(III) (As(III)-Fh; up to ∼40%) were recorded within the corroded ZVI bands. With regard to As(V) speciation, the inclusion of the distinct reference spectrum of As(V) incorporated into Mag (As(V)-Mag) (Figure S18), in addition to As(V) adsorbed onto Fh (As(V)-Fh), led to marked improvements of the LCF reconstructions of most spectra-relevant spectral regions. The highest LCF-derived As(V)-Mag fractions (up to 42%) were observed at the borders of the ZVI bands.

On Model B, 31 As μ-XANES spectra were recorded, and LCF revealed that As(V)-Fh and As(V)-Mag contributed up to 75 and 15%, respectively, mostly at the inflow to the upstream ZVI band and the periphery (Figures 5a, S24, and Table S7). As(III)-Fh contributed up to 91% and dominated in the center of the channel. Higher fractions of As(III) were associated with higher fractions of reduced Fe-phases and higher fractions of As(V) with higher fractions of ferric Fe-phases (Figure S5b). Some of the spectra, for example, Model B p_As_ 23, showed two oscillations in the postedge region that could not be reproduced with the available reference spectra (Figure S20c), indicating the presence of another As species. However, this did not affect the LCF results with regard to As redox speciation.

#### XAS Data Comparison and As Distribution

Brighter colors in optical microscopy images co-occurred with Lp and CaFeP, whereas darker colors correlated with GRC, Mag Viv, and metallic Fe, as indicated by Fe μ-XANES. In Model A, *p*_Fe_ 14 and *p*_Fe_ 15 were recorded within the dark and bright spots that underwent cyclic color changes (M2, Video 1) and corresponded to GRC (dark spot) and Lp (bright spot), respectively (Figures S19 and S20). Chemical imaging data in the same area showed that bright phases were associated with oxidized Fe-phases (Lp, CaFeP). In contrast, dark phases were not necessarily dominated by reduced/mixed valence Fe phases (Figure 4a, b). Thus, in areas with dark color but Fe(III)-dominated speciation, a minor amount of a mixed-valence Fe-phase induced the dark color despite the prevalence of Fe(III) solids (Figures S9 and S10). Full field data partially indicated GRC and Mag-like components in dark areas that appear Lp-dominated by chemical imaging (Figure S21a,b). This suggested that the Fe-precipitates were more oxidized on the surface probed by fluorescence spectroscopy compared to the interior probed by transmission spectroscopy. Taking all the XAS evidence into account, we conclude that in areas with cyclic color changes, the bright phase formed during flow consisted mainly of Lp and that the re-occurring dark phase during no-flow reflects the (partial) transformation of Lp into mainly GRC.

Truncated, reacted ZVI grains were visible in the reflected light image of Model A (Figure 4b, d). The ZVI core was coated by a Mag layer (Figures S19d–f and S20a), which marked the minimal extension of the color cycling. The third layer likely consisted of GRC contained below the top of the resin and filled parts of the empty spaces between the quartz grains. Further away from the ZVI grains, orange Lp dominated the pore space. This suggested that Mag was formed from GRC near the ZVI grains.

In addition to the Fe μ-XANES spectra, also the μ-EXAFS spectra were analyzed by LCF (Tables S9 and S10; Figures S7 and S8). For Model A, the EXAFS LCF results roughly corresponded to the XANES LCF results and indicated similar trends (Figure S22). For Model B, the EXAFS LCF was dominated by CaFeP, Lp, and GRC and showed less variability compared to the XANES LCF results (Figure S23). For the CaFeP and GRC, a general correlation between XANES and EXAFS was observed. On the other hand, Mag, Viv, and metallic Fe, that were included in the XANES LCF results, were not included in significant shares in the EXAFS LCF results (Figure S24). Differences between XANES and EXAFS LCF results most probably arise from stronger distortions in the former, resulting from high absorber concentrations, which lead to an overestimation of reduced phases because of the onset of the edge at lower energies. Sample inhomogeneity or detector nonlinearity may also contribute to distortions. In addition, discrepancies in the crystallinity between sample and reference phases and the different sensitivities of the XANES and EXAFS signals to the oxidation state and to the local order beyond the first shell are likely responsible for deviations. In the case of Model B, for example, Mag is mostly fitted to the XANES but not the corresponding EXAFS spectra, possibly indicating the presence of nanoscale Mag or Mag with a low degree of crystallinity.

The combination of the edge jump height normalized full field-derived Fe species distribution with the As distribution suggested that ∼40% of As was localized in the area of the Lp-like cluster, 34% in the area of the Mag-like cluster, and 26% in the area of the GRC-like cluster (excluding the metallic Fe cluster form the calculation, Figure S25). Integrated Fe XANES spectra, calculated from multiple pixels of the chemical images (frames and corresponding spectra in Figures S9–S16) revealed that As mainly occurred where the Fe speciation was represented by more than 50% of Fe(III)-phases with minor contributions of reduced phases (spectra 2 and 7 in Figure S10, spectra 1 and 2 in Figure S12, spectra 1 and 2 and 3 in Figure S14, and spectrum 3 in Figure S16). Despite the prevalent occurrence of Fe(III)-phases, these locations were all associated with dark color assumed to be induced by minor GRC or Mag admixture, in line with the As *K*-edge XANES data, which suggested that some of the As(V) was incorporated into Mag. Qualitatively, these observations derived from the chemical imaging results are in agreement with the interpretation of the full field data.

### Spatiotemporal Observations of Coupled Geochemical Processes in ZVI-Quartz Microflow Channels

From the combination of effluent data, time-resolved optical microscopy, and spatially-resolved synchrotron X-ray characterization of resin-impregnated flow channels, new insights into the interplay of hydrodynamics and geochemical processes in ZVI-sand filters were obtained in this work.

#### Mass-Balance Considerations

Based on an O_2_-saturated (∼8 mg/L) influent, the extent of Fe corrosion in Models A and B was estimated under the assumption that Fe was completely oxidized to Fe(III) or to 50% Fe(II) and 50% Fe(III) (based on full field XANES results for Model A, secondary Fe-phases were estimated to consist of 64% GRC, 19% Mag, and 17% Lp, corresponding to a Fe(II)/Fe(III) ratio of ∼1) (Table S4). Combined with the amounts of As, P, Si, and Ca retained in the micromodels (from influent and effluent concentrations), the molar ratios of retained As, P, Si, and Ca and of introduced bicarbonate over corroded Fe in Models A and B were derived (Table S4) and used in the discussion below.

#### Cyclic Color Changes and As Retention

With regard to the marked spatial color variations in Models A and B, XAS results of the resin-impregnated samples confirmed that orange colors were associated with Fe(III)-phases, represented by lepidocrocite and amorphous Ca-Fe-phosphate. Dark colors were associated with Fe(II)- and Fe(II/III)-phases, mainly carbonate green rust (GRC) and lower shares of magnetite and vivianite or a mixture of Fe(II), Fe(II/III), and Fe(III)-phases. This correlation could thus be exploited for the interpretation of time-resolved optical microscopy data collected during the operation of Model A.

In the upstream part of the microchannels, DO was introduced during water flow, which allowed for the oxic corrosion of the first ZVI band, that is, release of dissolved Fe(II) (eq 1), Fe(II) oxidation to Fe(III), and formation of Fe(III)- or Fe(II/III) oxides that provide sorption sites for P or As (eqs 3 and 4). Concomitantly, the oxidation of As(III) to more strongly sorbing As(V) was driven by co-oxidation with Fe(II).^49^ During no-flow, color changes in the upstream part of Model A indicated that after rapid DO depletion, remaining Fe(II) and Fe(II) from ongoing anoxic ZVI corrosion (eq 2) caused the transformation of Fe(III)-phases around the ZVI grains into Fe(II/III)-phases (eq 4). When flow was resumed, this reduction process was rapidly and nearly completely reversed, indicating that the dark phase was mainly GRC, which is rapidly oxidized by DO. XAS results confirmed that the observed cyclic phase transformation over flow/no-flow cycles was mainly attributed to transformations between lepidocrocite (bright zone) and GRC (dark zone), although the formation of a minor and more persistent magnetite fraction close to the ZVI grains also contributed to the observed color changes.^27^ The transformation of green rust to Lp has previously been reported to occur through a dissolution–oxidation–precipitation (DOP),^25,51,52^ with phosphate contributing to preferential formation of lepidocrocite over goethite.^10^ In contrast to GRC, lepidocrocite has a rather large window of stability and may not be rapidly transformed at circumneutral to slightly alkaline pH in the presence of adsorbed As.^27,30^ We therefore hypothesize that in analogy to the accelerated dissolution of inert γ-alumina (γ-Al_2_O_3_) in the presence of dissolved nickel (Ni(II)) or cobalt (Co(II)) and the subsequent precipitation of Ni–Al or Co–Al layered double hydroxides,^53^ dissolved Fe(II) may drive the transformation of lepidocrocite into GRC via a dissolution-precipitation process.^27,30^

Over multiple flow/no-flow cycles, a permanent dark zone resistant to oxidation gradually grew away from the ZVI grains. The Fe XAS data showed that Mag was formed in this zone, and the As XAS data indicated that As(V) became partly incorporated into Mag. This could be attributed to the transformation of GRC into Mag close to the ZVI, either through a redox^29^ or a nonredox reaction,^25^ also in agreement with the transformation of lepidocrocite to Mag via GRC.^30,54^ The prolonged no-flow phase in Model B leads to the absence of Lp/GRC cycling, suggesting that a larger amount of persistent Mag was formed, in agreement with XANES data. Overall, considering the molar ratio of bicarbonate to corroded Fe of 12–15 (Table S4), the observation of a large fraction of GRC in Model A was in line with a laboratory study, which showed that in the presence of Fe(II) and Fe(III) at low DO concentrations, GRC formation dominated over Mag formations at HCO_3_^–^/Fe ≥ 0.17.^54^

With increasing numbers of flow periods in Model B, effluent As(V) and P concentrations concomitantly decreased, but the As(III) concentration remained relatively constant, reflecting the more effective retention of As(V) and P than As(III) by the secondary Fe(II/III)-oxides.^36^ This also indicated that sufficient adsorption sites for As(V) and P were available after 20–25 days of operation; even at a high loading of 0.25–034 retained P per corroded Fe (Table S4). Previous work showed that As(V) was neither reduced nor released into solution upon the oxidation of sulfate green rust, but instead adsorbed to the surface of the newly formed lepidocrocite,^55^ whereas another study showed that nanoscale ZVI induced the reduction of adsorbed As(V).^18^ Significantly more As(III) was found in Model B compared to Model A. This could be attributed to enhanced As(III) adsorption on a larger mass of secondary Fe-phases at the later stage of the experiment or to the reduction of adsorbed As(V) during the prolonged no-flow period.^56^ Previous research showed that As(V) adsorbed onto magnetite was partly reduced to As(III) under anoxic conditions at pH 8.5.^57^

In Model A, As accumulation was correlated with the occurrence of repeated redox cycling (Figure 4e, Video 5). In contrast, the GRC further downstream did not undergo redox cycling, and its color remained green throughout the experiment, likely due to insufficient DO concentrations for GRC oxidation, and insignificant As accumulation was observed in this zone without GRC redox cycling. Previous research indicated that the transformation of GRC to magnetite was slowed down in the presence of As.^30^ Thus, the overlap of the zone of repeated lepidocrocite-GRC cycling with a zone of As accumulation may have resulted from delayed formation of magnetite in the presence of adsorbed As, which, on the other hand, did not hinder the oxidation of GRC with DO and subsequent formation of lepidocrocite. The combination of full field Fe XANES and As distribution data indicated that most As was retained where the speciation was dominated by Lp and Mag with smaller contributions from GRC. The Fe chemical imaging data combined with the As distribution data showed that As accumulations were always associated with high CaFeP and Lp (sum larger than 50%) and rarely with GRC despite the widespread occurrence of the latter phase. Although comparable Fe speciation distribution data for Model B are missing, a qualitative comparison between the μ-XANES results and As distribution data suggests that CaFeP and Mag positively correlated with the occurrence of As, whereas GRC precipitation did not co-occur with significant As retention (Figure 5). These correlated observations were in agreement with the reported reduced performance of GRC in As retention compared to magnetite.^39^

#### Phosphate and Ca Retention

In the effluent, the decrease in P concentration preceded the decrease of Ca, thus showing that quantitatively significant Ca removal required preceding phosphate removal. This can be attributed to the inhibition of CaCO_3_ precipitation (eq 6) at phosphate concentrations above 20 μM.^48^
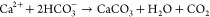
6

At the end of the operation of Models A and B, Ca, Fe, and P co-occurred in the upstream ZVI band (Figure S6a, b, f). In Model B, however, most Ca was retained as CaCO_3_ in one part of the downstream ZVI band (Figures S3d and S6a, c, g). For this to occur, water must have preferentially flown through this part of the ZVI band, the phosphate concentration was already sufficiently decreased, and the pH increased. The precipitation of CaCO_3_ led to H^+^ release (eq 6), which in turn enhanced anoxic ZVI corrosion and formation of H_2_ (eq 2). This coupling of multiple processes was supported by the observed formation of gas bubbles in this part of the microchannel and by the enhanced precipitation of Fe-solids downstream, which provided further sorption sites for As. These results show that the reactive layer geometry may critically influence the spatial sequence of geochemical reactions.

Based on the estimated molar ratios of retained Ca over corroded Fe in Models A and B (Table S4), about twice as much Ca was retained as CaCO_3_ than Fe by Fe(II/III)-phase formation upon ZVI corrosion. Combined with the relatively low density of CaCO_3_ (2.7 g/cm^3^ for calcite) compared to Fe-oxides, this suggested that CaCO_3_ precipitation consumed a larger share of the initial pore space compared to the secondary Fe-phases, in agreement with previous research.^58^

### Implications for Upscaled ZVI Filter Operation

The occurrence of GRC in Fe-based water treatment systems for As removal has previously been highlighted.^20,54,56,59,60^ This study emphasizes that GRC is particularly relevant as a transitional phase in water treatment with ZVI under intermittent flow. During no-flow, anoxic conditions allow for the formation of Fe(II) by anoxic ZVI corrosion, which in turn leads to the transformation of voluminous HFO phases to mixed-valence GRC and, incrementally, to dense magnetite. This transformation reduces the volume of the Fe-phases in the pore space and effectively enhances the hydraulic conductivity. The macroscopic result of this effect was demonstrated with flow through column experiments, which showed that intermittent flow (12 h/12 h flow/no-flow) prevented the rapid loss of hydraulic conductivity that was observed with constant flow (Section S3; Figures S26 and S27). Thus, intermittent flow operation of ZVI-based filters can delay pore clogging and requires lower hydraulic heads to maintain acceptable filtration rates as compared to constant operation via its impact on Fe phase transformations, but possibly also via its effects on CaCO_3_ formation. Our findings on Fe-phase formation and (cyclic) transformation may also be relevant with respect to the previously observed loss in hydraulic conductivity^61,62^ in ZVI-based permeable reactive barriers for groundwater remediation^61−64^ that could possibly be mitigated by periodic operation of push or pull wells.

### Implications for Geochemical Pore-Scale Investigations

This microfluidic study demonstrates the strengths and opportunities of combining spatiotemporal optical microscopy and X-ray micro-spectroscopy data with effluent data for the study of coupled geochemical and transport processes in porous media. Possible adaptations to explore in future research include the use of X-ray microspectroscopy to follow transformation reactions in situ with spatiotemporal resolution or the use of fluorescence microscopy in combination with suitable indicator compounds to probe chemical parameters, such as pH or DO in situ with spatiotemporal resolution. Moreover, microchannels with reduced geometric complexity can enable the implementation of reactive transport models for a quantitative description of the involved processes. Finally, such studies could benefit from a lab-on-a-chip approach for the simultaneous study of multiple samples for faster identification of key controls on geochemical processes at the pore scale.

## Supporting Information Available

Additional content on ZVI corrosion; arsenic removal; synthetic groundwater preparation; synchrotron experimentation; synchrotron data evaluation; and experiments with laboratory-scale columns to monitor hydraulic resistance increase (PDF)
Five Web-Enhanced Objects contain videos of (Video 1) Model A: optical microscopy data days 1–13 (MPG)
(Video 2): Model B: optical microscopy data days 43-49 (MPG)
(Video 3): Model B: time lapse video days 1 to 42 (MPG)
(Video 4) Model A: color threshold optical microscopy data of redox cycling (MPG)
(Video 5) Model A: semi-daily redox cycling arsenic distribution (MPG)

## Supplementary Material

es2c02189_si_001.pdf

es2c02189_si_002.mpg

es2c02189_si_003.mpg

es2c02189_si_004.mpg

es2c02189_si_005.mpg

es2c02189_si_006.mpg
