# Supplementary material for: Spatiotemporal Mineral Phase Evolution and Arsenic Retention in Microfluidic Models of Zerovalent Iron-Based Water Treatment
Source: Environ Sci Technol. 2022 Sep 12;56(19):13696–708. doi: 10.1021/acs.est.2c02189 (PMC9535812; doi:10.1021/acs.est.2c02189)
Supplement: Supplementary file 1 — es2c02189_si_001.pdf [file es2c02189_si_001.pdf]

Supporting Information for

Spatiotemporal mineral phase evolution and arsenic retention in microfluidic models of zerovalent iron based water treatment

Jonas Wielinski[1,*], Joaquin Jimenez-Martinez[1, 2], Jörg Göttlicher[3], Ralph Steininger[3], Stefan Mangold[3], Stephan J. Hug[1], Michael Berg[1], Andreas Voegelin[1,*]

[1] Eawag, Swiss Federal Institute of Aquatic Science and Technology, 8600 Dübendorf, Switzerland

[2] Department of Civil, Environmental and Geomatic Engineering, ETH Zürich, 8092 Zürich, Switzerland

[3] Institute for Photon Science and Synchrotron Radiation, Karlsruhe Institute of Technology, Hermann-von-Helmholtz-Platz 1, 76344 Eggenstein-Leopoldshafen, Karlsruhe, Germany.

* Corresponding authors:

Jonas Wielinski, Überlandstrasse 133, 8600 Dübendorf, Switzerland, email: jonas.wielinski@eawag.ch, office: +41 58 765 5336, fax: +41 58 765 5802

Andreas Voegelin, Überlandstrasse 133, 8600 Dübendorf, Switzerland, email: andreas.voegelin@eawag.ch, office: +41 58 765 5470, fax: +41 58 765 5802

This Supporting Information contains:

49 pages,
10 Tables, and
27 Figures.

Table of contents:

Section S1: As removal from groundwater in Bangladesh p. S2

Section S2: Supporting materials and methods p. S4

Section S3: Supporting experimental data p. S10

Section S4: Supporting Tables p. S13

Section S5: Supporting Figures p. S22

S1. ARSENIC REMOVAL FROM GROUNDWATER IN BANGLADESH

Arsenic (As) is a non-essential element and its occurrence in products dedicated for human consumption such as rice [1, 2] or drinking water [3] poses a threat to human health resulting from the mostly geogenic contamination of groundwater. The World Health Organization recommends an upper limit of 10 µg/L As in drinking water. In industrialized, high-income countries, large-scale treatment facilities are operated for As removal.[4] Despite global efforts to provide access to safe drinking water to everyone, 80 to 220 million people, mostly in Southeast Asian countries consume drinking water with geogenic As concentrations above 10 µg/L.[5] Depending on the geochemical composition of the water source, different As removal methods are applied. In Vietnam, anaerobic groundwater often contains ferrous iron (Fe(II)) that is oxidized to ferric iron (Fe(III)) when the water is pumped to the surface and aerated.[6] At near-neutral pH, Fe(III) rapidly precipitates as hydrous ferric oxides (HFO) to which As is adsorbed.[7, 8] This principle is used in sand filters with sufficient residence time for Fe(II) oxidation and precipitation to occur and thus to eliminate As from the filtered water.[9] In contrast, groundwater in other regions, for example Nepal, [10] Burkina Faso [11] and rural Bangladesh often contains concentrations of Fe(II) that are too low to effectively remove As, or higher levels of phosphate that interfere with As removal by competing for sorption sites.[9, 12] In this case, Fe(II) must be added artificially, *e.g.*, through the corrosion of zerovalent iron (ZVI), to enable the removal of As and P. In Bangladesh, the commercial ZVI-based, "SONO filter" provides such a low cost means for decentralized household-level As-removal from drinking water.[13, 14] ZVI corrosion is also a prominent method in the remediation of trichloroethylene or explosives from groundwater using permeable reactive barriers.[15-18]

The SONO filter contains a composite iron matrix (CIM, here denoted as ZVI), which corrodes in contact with aerated groundwater and releases Fe(II) into solution (eq. S1). At neutral to alkaline pH, Fe(II) is rapidly oxidized to Fe(III) that precipitates as HFO of varying crystallinity and structure such as ferrihydrite (Fh), lepidocrocite (Lp) or goethite (Gt) (HFO denoted as $Fe(OH)_3$, eqs. S2) [3, 14] or as carbonate green rust (GRC) at low dissolved oxygen (DO) concentrations.[19, 20]

$$2Fe^0 + O_2 + 4CO_2 + 2H_2O \rightarrow 2Fe^{2+} + 4HCO_3^- \qquad (S1)$$

$$2Fe^{2+} + 4HCO_3^- + \frac{1}{2}O_2 + H_2O \rightarrow 2Fe(OH)_3 + 4CO_2 \quad (S2)$$

GRC was discovered as a corrosion product in steel pipes[21] and thereafter also identified in natural systems such as soils or aquifers.[22-24] The GRC window of stability is narrow and GRC rapidly transforms into Lp, Fh or Gt in the presence of DO. By reaction with Fe^{2+}, on the other hand, GRC can transform into magnetite (Mag).[25] GRC has the ability to adsorb As(III) and As(V), [26-31] but also other oxyanions such as phosphate [32] or silicate [33]. The kinetics of GRC transformation and the nature of the products were shown to strongly depend on the water chemistry and adsorbed oxyanions.[25, 28, 34] GRC was reported to contribute to As removal in sediment remediation using ZVI-based permeable reactive barrier [20] and to As removal from drinking water using an electrocoagulation process.[29]

In reducing groundwater, As mainly occurs as As(III) (H_3AsO_3, arsenous acid / arsenite), which tends to be more mobile than As(V) (H_3AsO_4, arsenic acid / arsenate). At relevant pH values (7-9), arsenate occurs as negatively charged oxyanion ($H_2AsO_4^-$ / $HAsO_4^{2-}$), whereas arsenite mainly occurs as fully protonated species explaining its enhanced mobility. Through intermediates formed during the oxidation of Fe(II) → Fe(III), arsenite can be oxidized to arsenate [35] which then can strongly adsorb to the HFO formed during ZVI corrosion [36].

These processes occur during filtration of fresh water in SONO filters, when DO is continuously transported into the ZVI matrix. Without further water being added to the filter, the DO concentration in the stagnant water is depleted by ZVI corrosion. Thereafter, ZVI corrosion may continue by the reduction of protons (H^+) to hydrogen (H_2), a process that is significantly slowed as the pH increases concomitantly (eqn. S3).

$$Fe^0 + 2CO_2 + 2H_2O \rightarrow Fe^{2+} + H_2 + 2HCO_3^- \quad (S3)$$

The released dissolved Fe^{2+}, can induce the formation of the dense, mixed-valence Mag (ρ = 5.2 g/cm^3) from the lower density HFO or GRC.[3, 14, 25, 37]

$$2Fe(OH)_3 + Fe^{2+} + 2HCO_3^- \rightarrow Fe_3O_4 + 4H_2O + 2CO_2 \quad (S4)$$

This reaction reduces the molar volume of the Fe solids in the pores and thereby may also reduce the risk of pore clogging and increasing hydraulic resistance of the filter column. At the same time, Mag may also effectively immobilize As(III) through adsorption and As(V) through structural incorporation.[25]

Although a general understanding of the geochemical processes involved in the As removal in filters depending on the geochemistry of the raw water, has been acquired in the last decades, a mechanistic understanding of the coupling of these geochemical reactions with the hydrodynamics at the pore scale, is currently missing. Therefore, pore-scale studies are necessary to gain insights into these coupled processes that enable the design of more effective filter schemes. Furthermore, they are also essential for an improved understanding of the controlling processes in ZVI-containing reactive barriers for groundwater remediation.

S2. SUPPORTING MATERIALS AND METHODS

Chemicals and synthetic groundwater preparation. Quartz (CAS #: 14808-60-7, 50-70 mesh particle size, Sigma-Aldrich) was sieved to between 200 and 250 µm using two sieves (Retsch). ZVI (CAS #: 7439-89-6, -50/+100 mesh, 99.8+% metal basis, analysis results in Table S1, American Elements) was sieved to between 200 and 250 µm as well. $CaCO_3$, $NaHCO_3$, $Na_2SiO_3 \cdot 9\ H_2O$, $NaH_2PO_4 \cdot H_2O$ were obtained from Sigma Aldrich in analytical grade quality and used as received. Synthetic groundwater was prepared similar to previous work.[38] First $CaCO_3$ was added to doubly deionized (DDI) water and dissolved by purging the solution with CO_2 overnight, resulting in a pH of ca. 5.6. Solid $NaHCO_3$ was then added. Thereafter, $Na_2SiO_3 \cdot 9\ H_2O$ was added from a 20 g/L Si stock solution while the solution was stirred vigorously. Subsequently, air was passed through the solution to raise the pH to ≈ 7.0. Then, NaH_2PO_4 was added from a 20 g/L P stock solution. Air was passed through the solution until it reached pH 7.50. Arsenite was added from a 1000 mg/L $NaAsO_2$ stock solution. Finally, the pH was readjusted to 7.50 by further air/CO_2 purging. The synthetic groundwater was stored in the dark at 3 °C for up to 50 days. The pH increased to 7.6 after 2 weeks of storage time and was readjusted to 7.5 by air/CO_2 purging. The calculated concentrations of Na, Si, P, Ca, As, HCO_3^- were 3, 0.5, 0.1, 1.5, 0.0067, 4.9 mmol/L, respectively (Table S3).

Speciation of dissolved As by high performance liquid chromatography. Concentration of dissolved As(III) and As(V) were measured by high performance liquid chromatography (HPLC, Agilent 1260 Infinity II) coupled to an Agilent 8900QQQ ICP-MS (HPLC-ICP-MS) after adaption of a method by Raber et al.[39] Chromatographic separation was done using a Hamilton PRP-X100 HPLC column (150 mm × 4.1 mm, 5 µm particle size) after 20 µL injection and a 1 mL/min mobile phase flow with a malonic acid gradient from 4 to 40 mL at pH 5.2. The total run time was 6 min. Arsenic was measured using ICP-MS/MS with mass shift after reaction with O_2.

Microchannel impregnation. To impregnate the microchannels and prepare them for X-ray measurements, 24 mL of resin were mixed with 3 mL of hardener (ratio according to the manufacturer; EpoFix, Struers, Denmark), stirred thoroughly and degassed at 200 mbar for 20 min until no further gas bubbles appeared. The degassed mixture was filled into a disposable syringe (B. Braun) and injected into the aged microchannels at 100 µL/min for 5 min using a syringe pump (PHD Ultra, Harvard Apparatus). The resin was allowed to harden for 48 h in a glovebox under nitrogen (N_2) atmosphere (O_2 and H_2O < 0.1 ppm). The glass cover was removed using a diamond-file and a surgical blade. The resin cast was several micrometer elevated against the chip body due to a removed glue layer (Figure 1b). This elevation were removed on a diamond grindstone (Diamond sharpening system, DMT). Finally, the top was polished using diamond polishing pastes of 6 - 12 µm and thereafter 1 µm diamonds (FUTURO G10 and G01). From the polished microchannels, transmitted and reflected light optical microscopy images were recorded. The impregnated microchannels were stored in the glovebox prior to X-ray measurements. Models A and B were investigated using a benchtop micro-XRF spectrometer (M4, Tornado, Bruker) and Model B was additionally investigated by scanning electron microcopy including energy dispersive X-ray measurements (15 kV acceleration voltage, NanoSEM, FEI). These measurements were performed after the synchrotron measurements.

Synchrotron X-ray data acquisition. X-ray spectroscopy was performed at the KIT Light Source (Karlsruhe Institute of Technology, Germany). The electron storage ring was operated at 2.50 GeV and initially ca. 150 mA, the beam current constantly declining during operation. The storage ring was refilled

daily to 150 mA. At the SUL-X beamline, a 27 pole in-vacuum wiggler provides synchrotron light, which is monochromatized by a Si(111) double-crystal fixed exit monochromator (DCM). The monochromatized beam is focused by Kirkpatrick-Baez mirrors and cut by horizontal and vertical slits. The samples were kept in a huge vacuum chamber at 1.2×10^{-5} mbar at room temperature during three days of measurements. Three ionization chambers were used to record the transmission signal of the sample and of a Fe or Au foil used for energy calibration. A 7-channel silicon drift detector was used to record the X-ray fluorescence (XRF) signal. The cumulative counts within selected energy regions encompassing specific fluorescence lines (regions of interest) were recorded for P, Ca, Fe and As. Additionally, the Fe fluorescence pileup and Si escape peak were recorded, but mostly remained insignificant. A weak pileup was observed where ZVI grains were directly exposed to the surface.

Micro-focused XRF mappings (μ-XRF) at 12.5 keV (for Fe, Ca and As) and at 3.95 keV (below the Ca K edge for P measurements to avoid secondary P excitation from Ca $K\alpha$ fluorescence) were recorded (Model A: 10.70 x 5.14 mm^2 and Model B: 24.62 x 5.50 mm^2). The beam was focused to 40×40 μm^2. To display the μ-XRF data, the color intensity was scaled between 1 (for pixels with ROI counts exceeding or equal to the counts of 99.5% of all pixels) and 0 (minimum counts).

For micro focused X-ray absorption spectroscopy (μ-XAS) measurements at the As and Fe *K*-edges, the beam size was set to 50×50 μm^2 to reduce the flux density and thus prevent beam induced speciation changes in the sample. Fe μ-XAS spectra were recorded from 6978.5 eV to 7512.5 eV and As μ-XAS from 11,722 eV to 12,256 eV (both $k = 10$ Å^{-1}) with 0.3 eV steps around the edge in both cases.

Fe chemical images in four areas of interest (sizes in Table S2) were reconstructed from 20 Fe μ-XRF maps (beam size 40×40 μm^2) recorded at 20 energies around the Fe *K*-edge.[40] This data revealed the spatial distribution of Fe-phases and their relation to the colors in the microscopy images and to the As distribution.

Full field Fe X-ray absorption near edge structure (XANES) measurements for full field spectroscopic imaging were performed at the XAS beamline, where a 1.5 T bending magnet provides synchrotron light that is monochromatized by a Si(111) DCM.[41] The full field data acquisition setup has been previously

described.[42] Briefly, the X-ray beam of around 12×1.1 mm^2 passed through the sample at 90°. The transmitted beam hit a YAG fluorescence screen and the visible light image was recorded by a PCO-edge camera. Between the recording of the radiograms at increasing energy values, the sample was moved in and out of the beam by a hexapod (M-850.50 Hexapod, PI USA, operated at µm-precision) to determine the spatially distributed photon intensity with and without the sample. Per run, the method allowed the parallel recording of more than 500,000 XANES spectra with an effective 5×5 µm^2 pixel resolution, at 312 energies between 6962 and 7664 eV and with an 0.5 eV stepsize over 79 energies around the Fe *Kα*-edge. On Model A, 4 runs with about 200 µm vertical overlap were recorded to obtain a dataset of 2495×873 pixels covering an area of 11.84×4.14 mm^2.

XAS reference spectra. Reference spectra were recorded to analyze XAS data by linear combination fitting (LCF). For Fe, reference spectra of Mag (Sigmal Aldrich), 2- and 6-line Fh were measured in transmission mode at the SUL-X beamline, using the same settings as for µ-XAS measurements. A siderite spectrum was available but did not improve the fit quality. Reference spectra of Lp with a low degree of crystallinity and an Fe-phosphate precipitated in the presence of high Ca concentrations (CaFeP) were obtained from Senn et al. (2015).[43] A GRC reference spectrum was kindly provided by Case M. van Genuchten (Geologic Survey of Denmark and Greenland). A vivianite (Viv) reference spectrum recorded at the XAS beamline (KIT Light Source, Germany) was kindly provided by Mingkai Ma (University of Utrecht). The Viv sample was synthesized according to a method described in Al-Borno and Tomson (1994),[44] except that N_2-filled glove bag instead of an anoxic chamber was used for the synthesis.

For As, reference spectra of As(III) adsorbed onto goethite, $NaAsO_2$ and Na_2AsO_3 were recorded at the SUL-X beamline, using the same settings as for µ-XAS measurements. Reference spectra for As(III) and As(V) adsorbed onto ferrihydrite and goethite that had also been recorded at the SUL-X beamline were kindly provided by Hongyang Wang (KIT), and reference spectra for As(III) and As(V) adsorbed onto green rust and magnetite and of As(V) incorporated into magnetite [45] were provided by Case can Genuchten (Geological Survey of Denmark and Greenland). Based on preliminary LCF analyses, the reference spectra

of As(III) or As(V) adsorbed onto ferrihydride (Fh) (As(III)-Fh and As(V)-Fh) and of As(V) incorporated into magnetite (As(V)-Mag) were retained for the final XANES LCF analysis.

Evaluation of Fe *K*-edge µ-XAS data. For Fe, the transmission data were in general evaluated, with the exception of pFe 17 (Model A). Nevertheless, some spectra suffered at locations with very high Fe concentrations were distorted in both fluorescence and transmission mode because of over-absorption (fluorescence) or spectral compression (transmission). These spectra were removed from the analyses.

Normalization and background subtraction of spectra was performed using the Athena software code.[46] To extract the Fe *K*-edge EXAFS spectra, the AUTOBK algorithm implemented in the Athena software code was used with an R_{bkg} value of 0.85. The LCF analyses of the XANES and EXAFS data were performed using Matlab by solving

$$\arg(\min(\sum|\vec{\mu} - \mathbf{M}\vec{x}|^2)) \quad \text{(S5)}$$

using the Levenberg-Marquardt algorithm, where $\vec{\mu}$ is a vector containing the normalized X-ray absorption coefficients relevant for the LCF, $\mathbf{M}$ is a matrix containing the normalized X-ray absorption coefficients of the relevant reference material spectra and $\vec{x}$ is a vector containing the contributions of the individual reference material spectra (x_i), which were allowed to contribute with a factor between 0 and 3 and thus constrained to non-negative values. For each LCF, eq. (S5) was solved 200-times with randomly generated initial conditions (entries in $\vec{x}_0$) between 0 and 0.5 of each reference material spectrum. After fitting 200-times with varying initial conditions, the fit with the best result for eq. (S5) was accepted. For the automated analysis of a large number of spectra, this procedure yielded significantly improved results as opposed to constant initial condition, *e.g.*, $1/n \times [1,1,..,1]^T$, where n is the number of references. This evaluation procedure was used for the LCF to µ-XAS spectra and chemical imaging data. For µ-XAS LCF, Fe XANES was evaluated between 7108 and 7180 eV, and Fe EXAFS between k = 3 - 8 Å^{-1}. Arsenic XANES was evaluated between 11854 and 11939 eV. Reference spectra and LCF results are included in this SI (Figure S7, Figure S8, Figure S18 and Table S5 to Table S10). We recorded the normalized sum of squared residuals

(NSSR) for all XANES and EXAFS LCF, the fitting results (normalized individual fractions) and the sum of all fitted fractions. The energy shift was never fitted.

Evaluation of As *K*-edge µ-XAS data For As, the fluorescence XANES data was evaluated due to the generally lower As concentrations compared to Fe. All XANES were of sufficient quality and were analyzed in the same manner as the Fe XANES indicated above.

Evaluation of Fe chemical imaging data. In four areas of interest, chemical images were recorded at the Fe *K*-edge to determine the Fe speciation distribution. For the construction of chemical images, µ-XRF maps were recorded at 20 energies across the Fe *K*-edge (7040, 7080, 7110, 7114, 7115, 7117, 7118, 7119, 7120, 7122, 7125, 7128, 7130, 7132, 7135, 7140, 7170, 7200, 7250, and 7350 eV). The µ-XRF maps were stacked, and the normalized spectra in each pixel were evaluated by LCF. On each spectrum, ten LCF with varying, randomized initial component fractions were performed and the best fit was accepted (see above). Based on previously derived data quality criteria that account for data quality and the difference in the reference spectra, we find that in between 75% (area of interest 2) and 84.0% (area of interest 1) of all pixels, the dominant component was correctly determined.[40] The high uncertainty was related to the spectral similarity between the references in the XANES as we similarly showed for oxidized or sulfidized copper species.[40]

Evaluation of full field Fe XANES data. Around 2,200,000 full field XANES spectra were recorded on Model A. The spectra were normalized and a principle component analysis (PCA) was performed to identify the principal spectral components. The first 5 principle components were used to calculate the respective loadings at each point for the components. The second and third component showed in lot of cases either positive between 0.5 – 2 or negative values between -3 and -0.5. We therefore used in a three component RGB-plot. Zero was selected for negative values and one for positive values. For each area identified by cluster analysis, a representative spectrum was derived by averaging the normalized XANES spectra over a sub-area. All these computations were performed using custom scripts based on the Igor Pro software code (WaveMetrics).

The average spectra of the different clusters were interpreted by comparing them with relevant Fe reference spectra and μ-XANES spectra recorded on the microchannels (bold, black lines Figure S17). Based on their similarity to reference spectra, these clusters are referred to as Lp-like, Mag-like, GRC-like and metallic Fe-like (from top to bottom). In addition, the reference spectra used for the LCF analysis of μ-XANES spectra and chemical imaging data are displayed (dashed, blue curves). To relate the full field cluster spectra that suffer from spectral compression (due to locally enhanced absorber concentrations) to the μ-XANES data, selected spectra are displayed (dotted, red curves). In Figure S17 we use the labels $^{\alpha\text{-}\delta}$ to refer to the four average spectra calculated from the four clusters. $^{\alpha}$ LCF results for p_{Fe} 3 (Model B) are dominated by 65% CaFeP and 16% Lp, $^{\beta}$ p_{Fe} 61 (Model B; not included in the main analysis due to a low sum of fitted EXAFS fractions) is dominated by 33% GRC, 29% Mag and 21% CaFeP (NSSR = 0.00022), $^{\gamma}$ p_{Fe} 2 (Model A) is dominated by 46% GRC, 35% metallic Fe and 19% Viv, and $^{\delta}$ p_{Fe} 32 (Model A) is represented by 100% metallic Fe.

The combination of the full field and μ-XANES LCF results indicated that the Lp-like cluster is best viewed as representative of CaFeP and Lp, the Mag-like cluster as representative of a mixture of GRC and Mag (This explains the onset of the edge at lower energies compared to Mag and the light shoulder at energies just below the white line.) The GRC-like cluster can be seen as a mixture of GRC, metallic Fe, and Viv (This explains the onset of the edge at lower energies compared to pure GRC and the splitting of the white line). The metallic Fe-like cluster can be viewed as pure ZVI (suffering from extreme spectral compression and a resulting apparent shift of the onset of the edge to lower energies).

S3. SUPPORTING EXPERIMENTAL DATA

Head loss evolution in constant and intermittent laboratory flow columns. Two glass columns (d = 10 mm, L = 100 mm, Omnifit) were filled with 3 cm, 1 cm and 2 cm of quartz grains, ZVI grains and quartz grains from bottom to top, respectively (Figure S26). The quartz (Sigma Aldrich) and ZVI (American Elements, 99.8+% purity) grains were -50/+100 Mesh (d = 150 - 300 μm) (compare Materials and Methods section). In total, each column contained 5.4 g quartz and 3.0 g ZVI. Cotton was added to the top of the

upper quartz layer. In a trial experiment, column fittings were mounted on top and capillaries were used to transport the column effluent to the waste. However, oxygen diffusion through the fittings and capillaries led to Fe-hydroxides precipitation in the fittings and significantly impacted the head loss measurements. Operating the column with an open top prevented this issue. No red/orange Fe(III)-hydroxide precipitates were visible on the upper end of the quartz grains, *i.e.* DO diffusion from the top side was insignificant. Synthetic groundwater electrolyte as used in the microchannel experiments was passed through the columns in upflow mode, at a flow rate of 5 mL/h using two peristaltic pumps (Gilson). One column was operated continuously, the other column was operated intermittently with 12 h of flow followed by 12 h of no-flow (stagnation). Below the inflow of each column, an uncompensated gage pressure transducer (24PC series, 0-30 psi (0-2.07 bar) range, Honeywell) was installed using a T-piece. The transducer output signals were recorded by a memograph (Ecograph T RSG35, Endress+Hauser) using a 1 Hz sampling frequency and exported as 1-min average values. The pressure transducers were externally calibrated against a compressed air bottle pressure indicator between 0 and 2 bar at 10 points ($R^2 = 0.9993$).

Using Darcy's law (eq. S6) with the hydraulic gradient (i) and the hydraulic conductivity (K), (eq. S7) the relation between flux (J), volume flow (Q) and column cross section (A) and (eq. S8) the definition of the definition of the hydraulic gradient with hydraulic head (ΔH) and length of the column (L), we calculate the hydraulic conductivity (eq. S9). The hydraulic head (ΔH in m) is calculated from the pressure drop (in Pa) through the hydrostatic equation.

$$J = -Ki \qquad \text{(S6)}$$

$$J = \frac{Q}{A} \qquad \text{(S7)}$$

$$i = -\frac{\Delta H}{L} \qquad \text{(S8)}$$

$$K = \frac{L}{\Delta H}\frac{Q}{A} \qquad \text{(S9)}$$

The head losses/hydraulic conductivity of the columns in constant and intermittent flow mode are compared (Figure S27). The head loss of the intermittent flow column was around 0.1 bar in the beginning of the

experiment and stabilized at 0.11 to 0.12 bar before the end of the experiment. This translates into a hydraulic conductivity of 0.8×10^{-6} m/s, comparable to silty sand.[47] Although the head loss of the constantly operated column started off comparably, it continuously increased until the end of the experiment and the average hydraulic conductivity in the column decreased to around 10^{-7} m/s. Therefore, we conclude the intermittent flow operation to be beneficial at preventing a rapid increase in hydraulic resistance, at least on short time scales. Although pressure drop and changes in hydraulic conductivity were averaged over the entire length of the column, significant changes likely resulted mostly from processes occurring in the ZVI layer and shortly above.

We propose that through geochemical reactions observed in the microchannels, namely, the repeated transformation of Lp or GRC during no-flow periods and subsequent precipitation of Mag from GRC sufficient pore space remained available for the filtration of water with only moderate increase in hydraulic resistance. Additionally, stopping and restarting the flow sent a short pulse through the intermittent column. This pulse could cause the periodic detachment and transport of particles as observed in the microchannel (M3-4, WEO 1). Downstream quartz grains may act as colloid collectors (Figure S6d,e). This effect is likely beneficial to reduce hydraulic resistance originating from secondary Fe-phases formed in the ZVI layer. However, it cannot account for the large differences between the two operation modes.

S4. SUPPORTING TABLES

Table S1: Impurities of the ZVI used in this study as indicated by the manufacturer (American Elements).

Analysis	C	S	O	P	Si	Mn	Cu	Ni	Mo	Cr	V
%	0.003	0.01	0.13	0.004	0.002	0.11	0.11	0.06	0.04	0.05	0.001

Table S2: Experimental parameters, properties and means of investigation employed for the two microchannels (Models A and B), which were used to represent ZVI / quartz filters for As removal from groundwater. Masses of ZVI and quartz were obtained using a balance during model preparation and the pore volume was calculated from the empty microchannel volume and the masses and densities of ZVI and quartz. The filtered pore volumes were calculated from the volume of groundwater passed through the micro flow channel. The mass of accumulated As was determined from the water flow and the difference between the influent and the effluent As concentrations.

Property / Model	Model A	Model B
Experimental runtime	13 day	49 day (44 days flow)
ZVI (mg)	23	21
Quartz (mg)	96	110
Pore volume (μL)	23.3	18.4
Pore volume (%)	37	29
Filtered pore volumes	2006	8600
Accumulated As (μg)	16 (days 1-11)	40
Laboratory camera images	-	Days 1-43
Optical microscopy images	Days 1-13	Days 44-49
Collected effluent fractions	12 fractions / flow period (days 1-11)	1 fraction / flow period (days 1-36 and days 41-49)
Available elements from synchrotron μ-XRF	Fe, Ca, P, As (10.70 x 5.14 mm^2)	Fe, Ca, As (24.62 x 5.50 mm^2)
Fe μ-XAS spectra	24 points	69 points
As μ-XAS spectra	40 points	31 points
Chemical images	4 areas of interest:<br>1: 1.76×0.84 mm^2<br>2: 1.84×1.02 mm^2<br>3: 1.58×1.10 mm^2<br>4: 2.00×0.92 mm^2	N/A
Full field Fe XANES available	ca. 11×4 mm^2, ca. 2.2×10^6 pixel	N/A

Table S3: Target (calculated) and measured concentrations of Na, Si, P, Ca and As in synthetic groundwater in mmol/L (mM) or mg/L. Two standard deviations are included for the values of the measured concentrations.

	Calculated concentration (mM)	Calculated concentration (mg/L)	Measured concentrations (mg/L)	Excess measured concentration (%)
Na	3.0	69.0	73.4 ± 1.5	6
Si	0.5	14.0	16.4 ± 0.1	17
P	0.1	3.1	3.5 ± 0.4	13
Ca	1.5	60.0	62.5 ± 0.5	4
As	0.0067	0.50	0.57 ± 0.06	14

Table S4: Molar concentrations of introduced and retained compounds, estimates for corroded ZVI and the molar ratios between the compounds introduced with the influent and available Fe. [a] For Model A only day 1-11 out of 13 days total runtime are considered. [b] Assumption is that the influent contains 8 mg/L DO. [c] Assumptions are that the influent contains 8 mg/L DO and the Fe(II)/Fe(III) ratio is 1 as derived from full field XANES data. [d] Assumption is that the Fe(II)/Fe(III) ratio is unit, as for Model A.

	Compound	Total introduced (µmol)	Retained (µmol)	ZVI corroded: oxic, Fe(III) (µmol) [b]	ZVI corroded: oxic, Fe(II/III) (µmol) [c]	Molar ratios of $X_{introduced}$/Fe	Molar ratios of $X_{retained}$/Fe
Model A [a]	Ca	62	25	13	17	3.8 - 4.7	1.5 - 1.9
	Silicate	23	nd			1.4 - 1.7	nd
	Phosphate	4.5	4.4			0.27 - 0.34	0.27 - 0.33
	As	0.30	0.17			0.018 - 0.023	0.010 - 0.013
	Bicarbonate	194	nd			12 - 15	nd
Model B [d]	Ca	248	113	53	66	3.8 - 4.7	1.7 - 2.1
	Si	94	20			1.4 - 1.7	0.31 - 0.38
	P	18	17			0.27 - 0.34	0.25 - 0.32
	As	1.2	0.54			0.018 - 0.023	0.0081 - 0.010
	Bicarbonate	776	nd			12 - 15	nd

Table S5: Model A. Fe *K*-edge XANES LCF results. Fractions (in %) of metallic Fe, vivianite (Viv), carbonate green rust (GRC), magnetite (Mag), lepidocrocite with a low degree of crystallinity (Lp) and amorphous Fe-phosphate precipitated in Ca-containing background electrolyte (CaFeP), normalized to a sum of 100%. Effective sum of fitted fractions is listed. The normalized sum of squared residuals (NSSR) corresponds to the r-factor from Athena.[46] The locations of the spectra are indicated in Figure S1c.

p_{Fe}	Metallic Fe	Viv	GRC	Mag	Lp	CaFeP	Sum of fitted fractions	NSSR
2	35	19	46	0	0	0	1.02	0.00114
3	100	0	0	0	0	0	1.30	0.04615
4	4	10	6	22	31	28	1.02	0.00027
5	12	6	8	18	31	25	1.13	0.00022
6	2	23	60	11	0	5	1.02	0.00019
7	12	26	62	0	0	0	1.02	0.00012
8	96	4	0	0	0	0	1.26	0.01680
9	21	41	38	0	0	0	1.03	0.00035
10	4	13	35	26	0	21	1.01	0.00016
14	3	9	59	28	1	0	0.99	0.00023
15	2	0	0	0	67	31	1.01	0.00021
16	7	0	0	0	38	55	1.17	0.00038
17	27	16	13	9	33	1	1.09	0.00005
18	14	2	0	16	18	50	1.02	0.00069
19	15	0	0	6	17	62	1.04	0.00062
20	7	1	0	6	61	26	1.05	0.00009
27	9	0	0	4	58	29	1.09	0.00008
31	13	0	0	2	64	20	1.04	0.00013
32	100	0	0	0	0	0	1.24	0.02225
33	33	13	54	0	0	0	1.06	0.00037
34	16	10	49	14	3	8	1.05	0.00014
50	37	24	2	0	24	13	1.05	0.00019

Table S6: Model B. Fe *K*-edge XANES LCF results. Fractions (in %) of metallic Fe, vivianite (Viv), carbonate green rust (GRC), magnetite (Mag), lepidocrocite with a low degree of crystallinity (Lp) and amorphous Fe-phosphate precipitated in Ca-containing background electrolyte (CaFeP), normalized to a sum of 100%. Effective sum of fitted fractions is listed. The normalized sum of squared residuals (NSSR) corresponds to the r-factor from Athena.[46] The locations of the spectra are indicated in Figure S3b.

p_{Fe}	Metallic Fe	Viv	GRC	Mag	Lp	CaFeP	Sum of fitted fractions	NSSR
2	11	8	3	37	1	40	1.03	0.00040
3	6	6	0	6	15	66	1.03	0.00041
5	0	8	5	22	10	55	1.00	0.00038
7	2	3	0	41	2	52	1.03	0.00035
8	10	6	2	18	7	56	1.02	0.00052
12	6	7	8	26	10	43	0.97	0.00027
15	10	4	0	6	16	64	1.08	0.00079
16	4	5	0	9	21	62	1.05	0.00028
17	6	10	6	29	9	41	1.02	0.00040
19	8	17	9	25	6	36	1.00	0.00039
20	0	5	8	18	12	57	1.05	0.00044
21	1	8	9	42	2	38	1.06	0.00021
26	16	27	32	2	2	21	0.96	0.00136
32	0	12	1	31	12	45	1.04	0.00032
33	13	6	0	7	22	52	1.05	0.00046
34	1	3	4	25	13	54	1.05	0.00037
38	3	6	1	9	21	60	1.08	0.00019
42	7	18	52	16	2	5	0.97	0.00027
44	0	13	53	24	0	10	0.95	0.00063
45	1	8	10	38	4	38	1.02	0.00017
46	7	8	21	26	0	38	1.00	0.00030
48	13	5	3	9	12	59	1.00	0.00048
50	12	8	2	12	9	57	1.00	0.00081
59	18	9	0	5	17	51	1.10	0.00090
62	1	11	14	20	6	48	1.03	0.00030
63	6	4	22	32	0	36	1.18	0.00022
64	0	12	45	25	0	18	0.98	0.00043
65	0	14	52	21	0	13	0.98	0.00092
66	0	10	58	19	0	13	0.93	0.00097
68	0	12	11	31	2	44	0.99	0.00048
69	4	8	8	25	12	42	1.03	0.00041

Table S7: Model A. As *K*-edge XANES LCF results (in %). As(III)-Fh and As(V)-Fh correspond to As(III) or As(V) adsorbed onto ferrihydrite, respectively. As(V)-Mag corresponds to As(V) incorporated into magnetite. Effective sum of fitted fractions is listed. The normalized sum of squared residuals (NSSR) corresponds to the r-factor from Athena.[46] The locations of the spectra are indicated in Figure S1d.

p_{As}	As(III)-Fh	As(V)-Fh	As(V)-Mag	Sum of fitted fractions	NSSR
1	19	46	35	0.97	0.000454
2	30	48	22	0.98	0.000175
3	22	46	31	0.97	0.000226
4	29	51	20	0.98	0.000801
5	22	43	35	0.97	0.000202
6	23	60	17	0.98	0.000144
7	18	74	8	0.99	0.002779
8	24	50	26	0.97	0.000339
9	21	55	25	0.98	0.000122
10	18	68	14	0.98	0.002541
11	21	53	25	0.97	0.000098
12	39	41	20	0.95	0.004399
13	26	42	32	0.98	0.000799
14	21	63	16	0.98	0.001090
15	31	63	6	0.97	0.003816
16	20	63	18	0.99	0.001643
17	13	64	24	1.00	0.000595
18	22	51	27	0.98	0.000182
19	34	37	29	0.97	0.000247
20	14	63	23	0.98	0.000551
21	22	46	32	0.98	0.000180
22	17	52	31	0.98	0.000674
23	29	47	24	0.98	0.000208
24	26	51	23	0.97	0.000084
25	28	46	27	0.99	0.000325
26	15	68	17	0.98	0.001564
27	16	50	34	0.98	0.000468
28	25	63	12	0.98	0.001334
29	18	54	28	0.98	0.000093
30	25	53	22	0.98	0.000184
31	29	51	21	0.98	0.000762
32	20	57	23	0.98	0.000101
33	28	53	19	0.98	0.000120
34	28	42	30	0.98	0.000229
35	19	60	20	0.98	0.000937
36	13	45	42	0.97	0.000377
37	15	63	22	0.98	0.000733
38	31	38	31	0.98	0.000788
39	16	64	20	0.97	0.000838
40	25	44	31	0.97	0.000075

Table S8: Model B. As *K*-edge XANES LCF results (in %). As(III)-Fh and As(V)-Fh correspond to As(III) or As(V) adsorbed onto ferrihydrite, respectively. As(V)-Mag corresponds to As(V) incorporated into magnetite. Effective sum of fitted fractions is listed. The normalized sum of squared residuals (NSSR) corresponds to the r-factor from Athena.[46] The locations of the spectra are indicated in Figure S3c.

p_{As}	As(III)-Fh	As(V)-Fh	As(V)-Mag	Sum of fitted fractions	NSSR
15	24	76	0	1.01	0.001276
16	37	54	8	1.00	0.000473
17	68	20	12	0.99	0.000633
18	78	13	8	0.99	0.000689
19	58	35	7	0.99	0.000519
20	40	55	5	0.99	0.000548
21	71	24	5	1.00	0.000590
22	90	5	5	0.97	0.001814
23	91	2	7	0.98	0.002074
24	85	10	5	0.99	0.001849
25	71	23	6	0.99	0.000710
26	82	7	11	0.99	0.001566
41	78	16	5	0.98	0.001173
42	76	13	11	0.99	0.001632
43	80	11	9	0.98	0.001136
44	84	7	8	0.99	0.001244
45	62	33	5	1.00	0.000492
46	59	37	4	1.00	0.000531
47	69	26	5	0.99	0.000595
48	44	52	4	0.99	0.000350
49	45	55	0	0.98	0.000393
50	46	49	4	0.98	0.000263
51	82	14	4	0.97	0.001216
52	79	12	9	0.98	0.000560
53	36	56	8	0.98	0.000300
54	40	45	16	0.98	0.000315
55	52	40	8	0.98	0.000247
56	69	23	9	0.98	0.000252
57	74	15	11	0.98	0.000525
58	59	32	9	0.99	0.000602
59	23	65	12	1.00	0.000603

Table S9: Model A. Fe *K*-edge EXAFS LCF results. Fractions (in %) of metallic Fe, vivianite (Viv), carbonate green rust (GRC), magnetite (Mag), lepidocrocite with a low degree of crystallinity (Lp) and amorphous Fe-phosphate precipitated in Ca-containing background electrolyte (CaFeP), normalized to a sum of 100%. Effective sum of fitted fractions is listed. The normalized sum of squared residuals (NSSR) corresponds to the r-factor from Athena.[46] The locations of the spectra are indicated in Figure S1c.

p_{Fe}	Metallic Fe	Viv	GRC	Mag	Lp	CaFeP	Sum of fitted fractions	NSSR
2	0	0	96	0	4	0	0.70	0.30257
3	13	5	67	0	1	13	0.12	0.40920
4	0	5	4	6	65	20	0.80	0.04131
5	0	8	9	15	51	16	0.96	0.16501
6	0	6	81	0	6	8	0.83	0.20276
7	0	2	98	0	0	0	0.84	0.02431
8	5	3	92	0	0	0	0.30	0.04721
9	3	17	80	0	0	0	0.68	0.04940
10	1	16	42	0	19	22	0.84	0.09192
14	0	2	87	0	12	0	0.82	0.04356
15	0	0	0	1	72	26	1.08	0.14787
16	0	0	10	32	15	43	1.18	0.55097
17	4	9	43	0	24	19	0.70	0.09302
18	0	2	3	4	89	2	0.76	0.01196
19	0	0	0	2	63	35	0.82	0.15252
20	0	3	4	2	90	1	0.97	0.01292
27	0	0	3	0	82	15	0.95	0.05585
31	3	21	0	0	76	0	1.13	0.20672
32	97	0	0	0	0	3	0.34	0.11757
33	9	20	71	0	0	0	0.88	0.17867
34	0	7	78	0	5	9	0.86	0.20928
50	7	28	16	0	15	34	0.66	0.26465

Table S10: Model B. Fe *K*-edge EXAFS LCF results. Fractions (in %) of metallic Fe, vivianite (Viv), carbonate green rust (GRC), magnetite (Mag), lepidocrocite with a low degree of crystallinity (Lp) and amorphous Fe-phosphate precipitated in Ca-containing background electrolyte (CaFeP), normalized to a sum of 100%. Effective sum of fitted fractions is listed. The normalized sum of squared residuals (NSSR) corresponds to the r-factor from Athena.[46] The locations of the spectra are indicated in Figure S3b.

p_{Fe}	Metallic Fe	Viv	GRC	Mag	Lp	CaFeP	Sum of fitted fractions	NSSR
2	0	0	13	4	15	68	0.66	0.09795
3	0	0	0	7	10	84	0.82	0.05974
5	0	0	25	5	0	70	0.80	0.16309
7	1	0	0	0	17	82	0.75	0.08030
8	0	0	3	0	16	80	0.71	0.05724
12	0	0	6	3	32	60	0.68	0.07373
15	2	0	0	0	0	98	0.93	0.25963
16	0	0	0	0	7	93	0.94	0.08065
17	0	0	8	0	15	77	0.68	0.04160
19	0	0	26	0	13	61	0.76	0.16517
20	0	0	11	0	20	69	1.00	0.22022
21	0	0	10	0	12	78	0.68	0.13670
26	0	0	63	0	12	24	0.69	0.36996
32	0	0	0	0	24	76	0.70	0.05463
33	5	0	0	0	8	87	0.89	0.12764
34	2	0	0	17	8	73	0.74	0.28362
38	0	0	0	6	16	78	0.82	0.08437
42	0	0	75	0	0	25	0.68	0.02536
44	0	0	63	0	8	30	0.75	0.08895
45	0	0	13	0	5	82	0.95	0.39534
46	0	0	25	1	21	53	0.70	0.03328
48	0	0	0	9	2	88	0.72	0.03791
50	0	0	0	3	12	86	0.67	0.03322
59	0	0	0	6	21	73	0.72	0.09254
62	0	0	17	0	24	59	0.80	0.05432
63	0	0	18	0	17	66	0.78	0.10949
64	0	0	53	0	12	35	0.71	0.02813
65	0	0	69	2	11	19	0.76	0.04964
66	0	0	62	0	5	33	0.72	0.06735
68	0	0	12	0	14	75	0.68	0.03170
69	0	0	0	0	20	80	0.72	0.02759

S5. SUPPORTING FIGURES

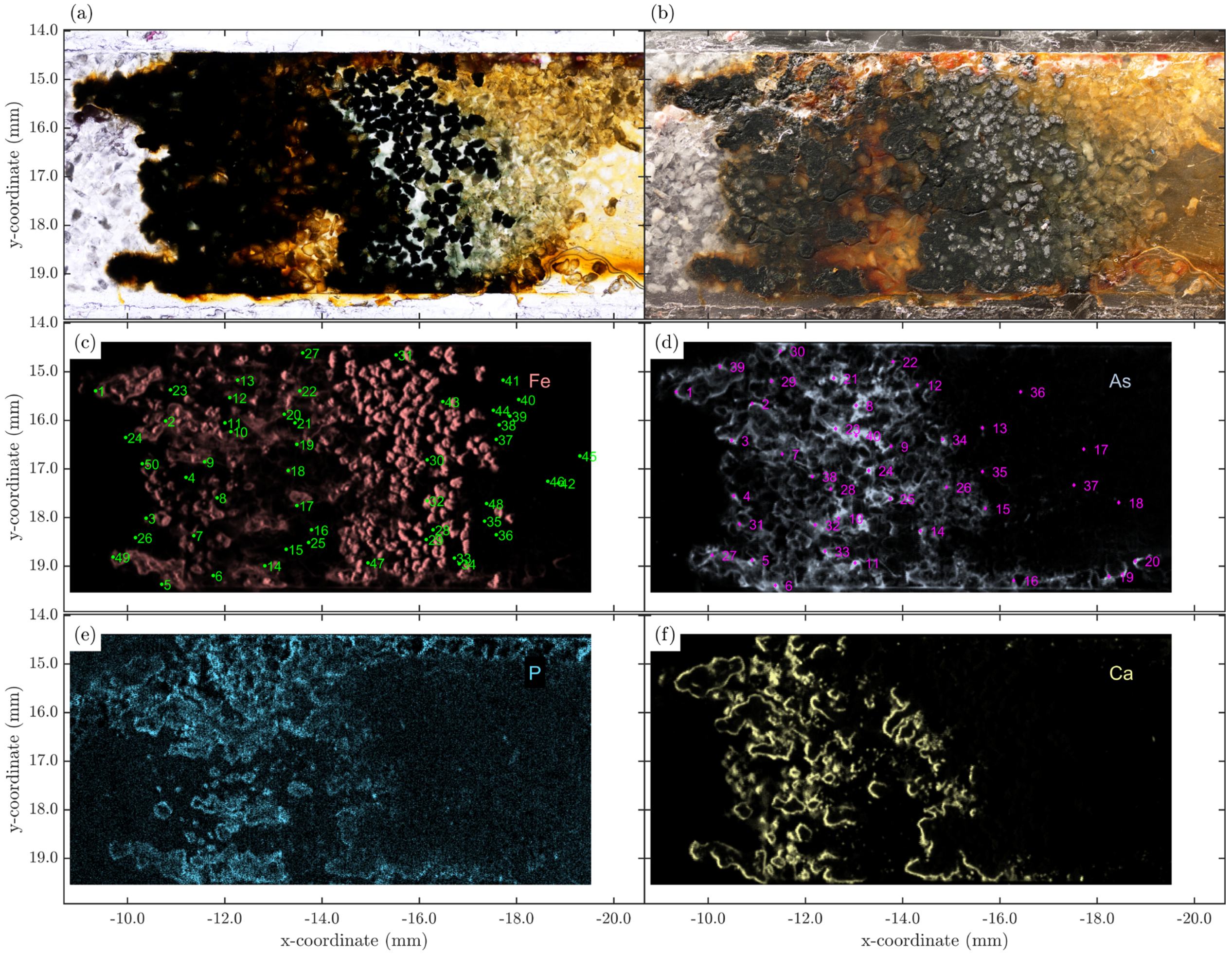

Figure S1: Model A. (a) Transmitted and (b) reflected light optical microscopy images. Panels (d-f) show the distribution of Fe, As, P and Ca, respectively. The numbers in (c-d) indicate the locations of the recorded µ-XAS data for Fe and As, respectively. Numbers and locations included in this graph but not in the main text indicate locations in which the quality of the recorded data was insufficient to be included. The corresponding information is indicated in Figure 4, but including annotations and tri-color maps instead of single element maps.

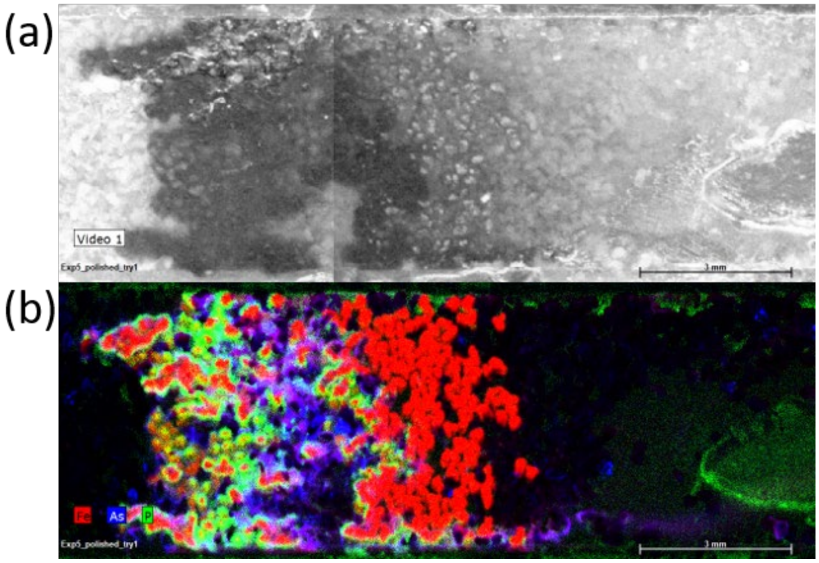

Figure S2: Model A. (a) Optical image and (b) Fe, As and P distributions determined using a laboratory micro X-ray fluorescence (XRF) spectrometer (silver (Ag) tube, emission at ca 22 keV, M4 Tornado, Bruker). The data in (b) can be compared to the synchrotron μ-XRF data displayed in Figure 4c.

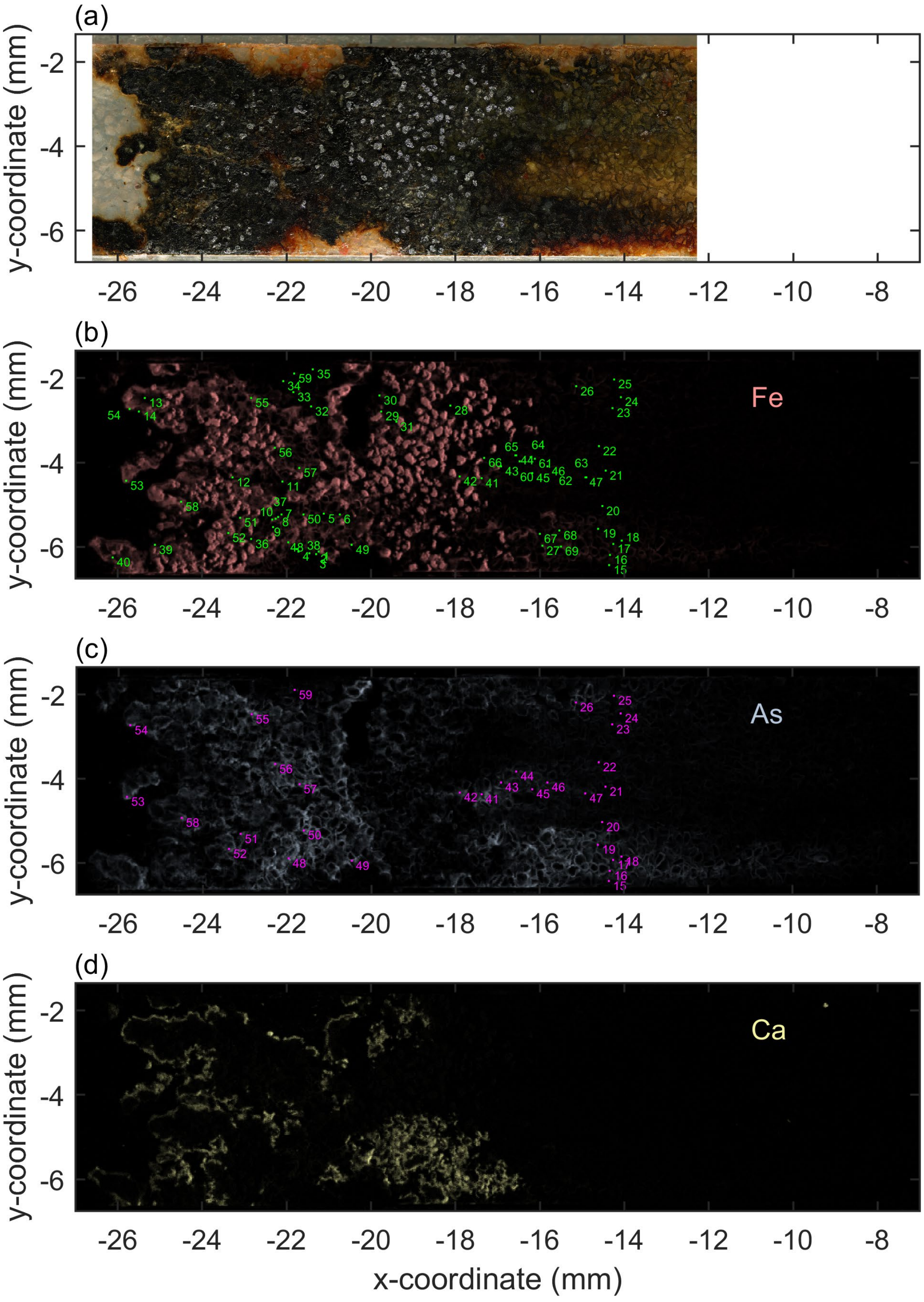

Figure S3: Model B. (a) Reflected light optical microscopy image. Panels (b-d) show the distribution of Fe, As and Ca, respectively. The numbers in (b-c) indicate the locations of the recorded μ-XAS data for Fe and As, respectively. Numbers and locations included in this graph but not in the main text indicate locations in which the quality of the recorded data was insufficient to be included. The corresponding information is indicated in Figure 5, but including annotations and tri-color maps instead of single element maps.

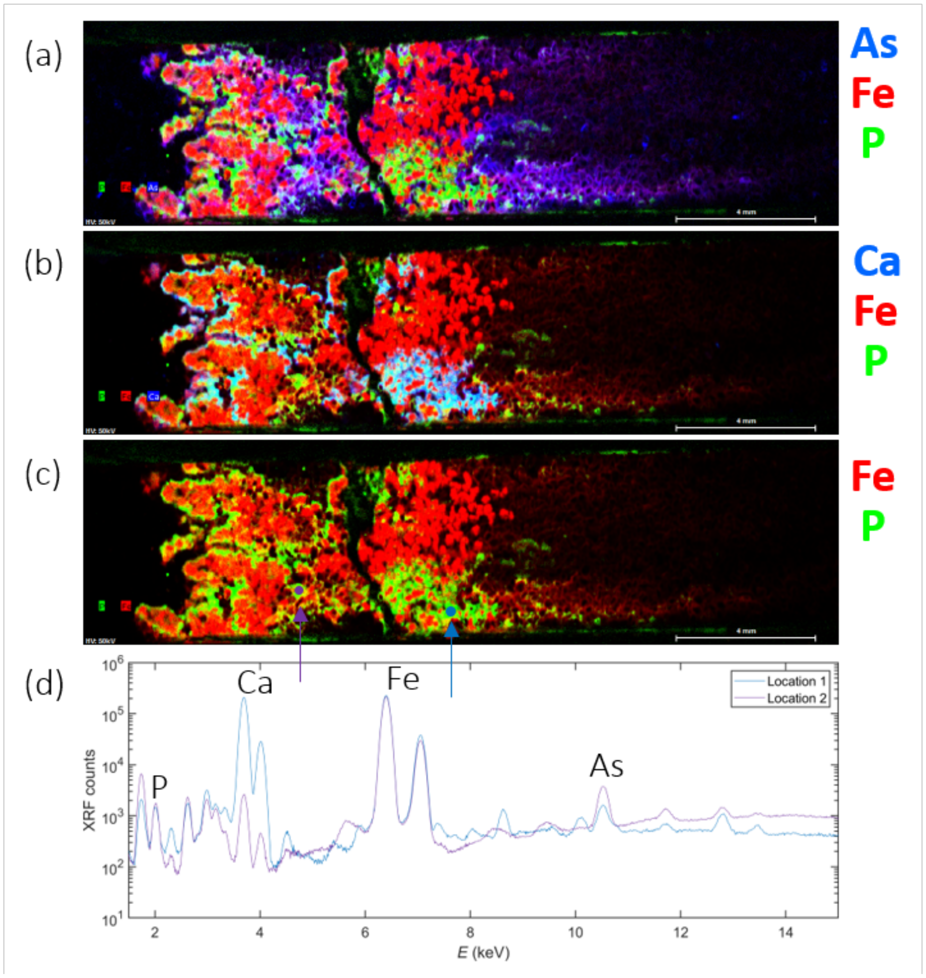

Figure S4: Model B. (a-c) Fe, Ca, P and As distributions determined using a laboratory micro X-ray fluorescence (XRF) spectrometer (Ag tube, emission at ca. 22 keV M4, Tornado, Bruker) in different combinations. The data can be compared to synchrotron µ-XRF displayed in Figure 5b. (d) XRF spectra at two locations indicated in (c). In Location 1 (blue) significant Ca precipitations occurred. Calcium excitation and subsequent Ca *K*α XRF leads to secondary P excitation. Therefore in Location 1 and wherever high Ca concentrations were reported, the P concentration is overestimated. In Location 2, only low Ca signal was observed. This was confirmed by scanning electron microscopy (SEM) energy dispersive X-ray spectroscopy (EDX) data presented in Figure S6.

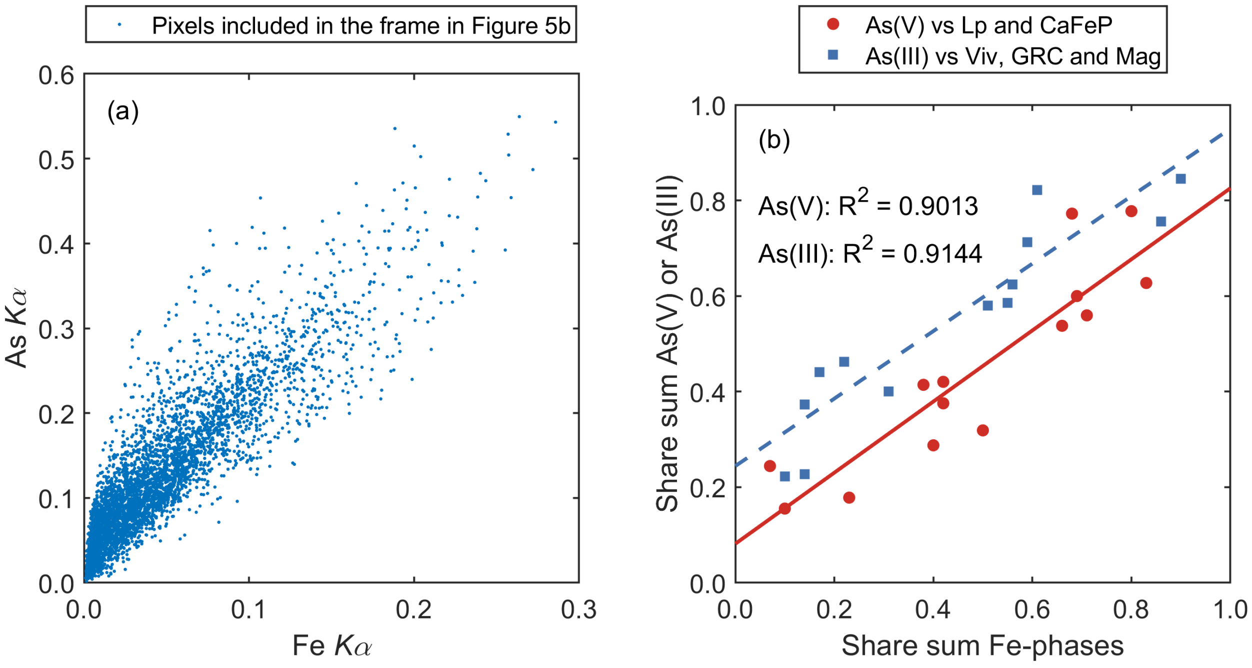

Figure S5: Model B. (a) Arsenic *versus* Fe *K*α fluorescence intensities in pixels displayed in the white frame in Figure 5b. (b) LCF-derived sum of As(V)-Fh and As(V)-Mag fractions *versus* only Fe(III) containing phases (Lp, CaFeP) (bold, red line) and As(III)-Fh fractions *versus* LCF-derived Fe(II)-containing phases (Viv, GRC, Mag) (dashed, blue line). For both, As and Fe, the XANES LCF data were used.

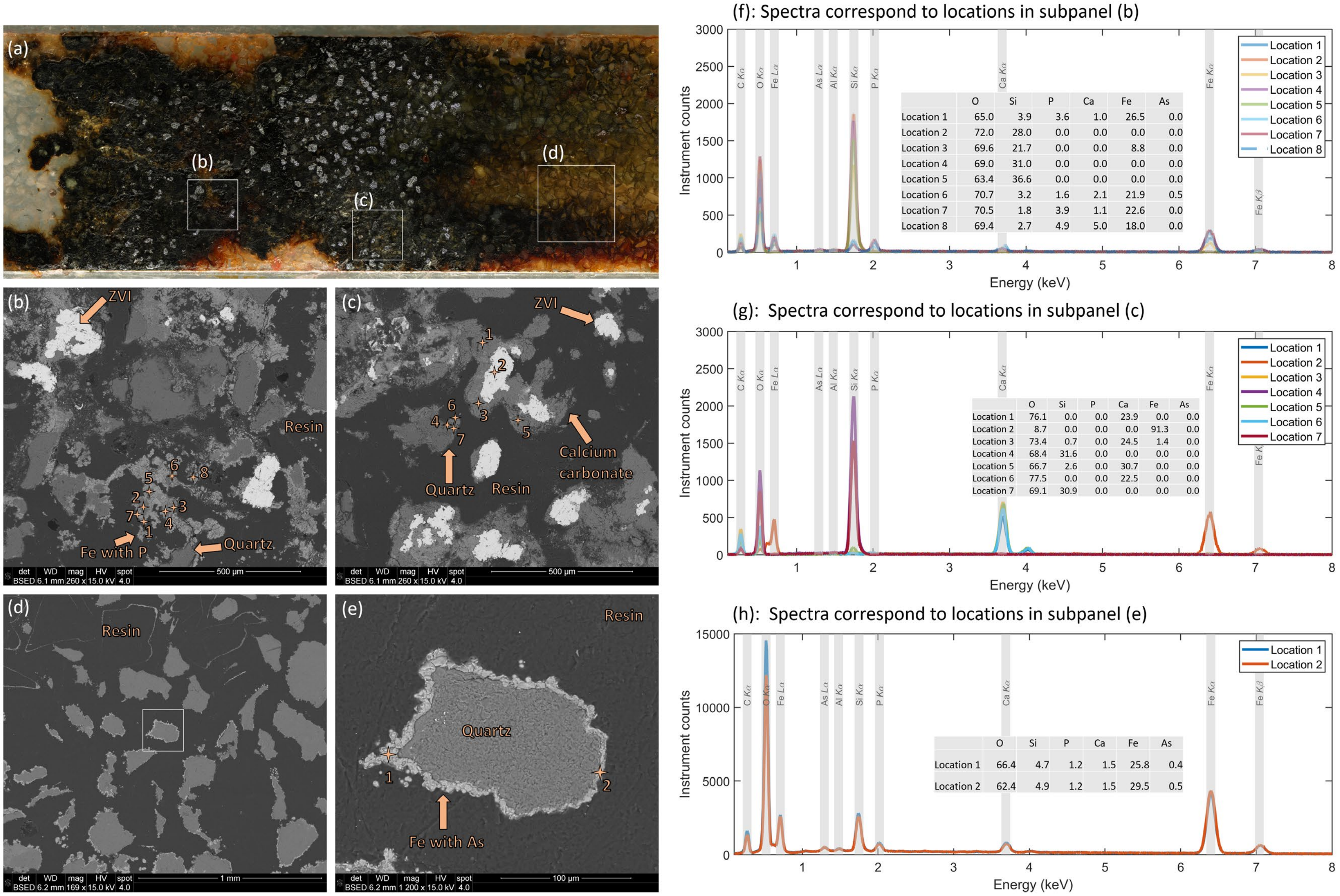

(f)

	O	Si	P	Ca	Fe	As
Location 1	65.0	3.9	3.6	1.0	26.5	0.0
Location 2	72.0	28.0	0.0	0.0	0.0	0.0
Location 3	69.6	21.7	0.0	0.0	8.8	0.0
Location 4	69.0	31.0	0.0	0.0	0.0	0.0
Location 5	63.4	36.6	0.0	0.0	0.0	0.0
Location 6	70.7	3.2	1.6	2.1	21.9	0.5
Location 7	70.5	1.8	3.9	1.1	22.6	0.0
Location 8	69.4	2.7	4.9	5.0	18.0	0.0

(g)

	O	Si	P	Ca	Fe	As
Location 1	76.1	0.0	0.0	23.9	0.0	0.0
Location 2	8.7	0.0	0.0	0.0	91.3	0.0
Location 3	73.4	0.7	0.0	24.5	1.4	0.0
Location 4	68.4	31.6	0.0	0.0	0.0	0.0
Location 5	66.7	2.6	0.0	30.7	0.0	0.0
Location 6	77.5	0.0	0.0	22.5	0.0	0.0
Location 7	69.1	30.9	0.0	0.0	0.0	0.0

(h)

	O	Si	P	Ca	Fe	As
Location 1	66.4	4.7	1.2	1.5	25.8	0.4
Location 2	62.4	4.9	1.2	1.5	29.5	0.5

Figure S6: Model B. SEM-EDX data. (a) Reflected light optical microscope image with locations. Areas mapped by SEM-EDX and displayed in panels (b-d) are marked by white rectangles. (b-e) Micrographs recorded by scanning electron microscopy (SEM) (FEI NanoSEM, 15 kV, back scattered electron detection). In panel (d), the area probed in panel (e) is indicated by a white frame. (f-h) Energy dispersive X-ray fluorescence (EDX) spectra recorded on locations indicated in panels (b), (c) and (e). Calculated compositions in each spectrum are indicated in tables. Orange labels and arrows in (b-d) describe the phases that are observed in the micrographs based on the EDX results.

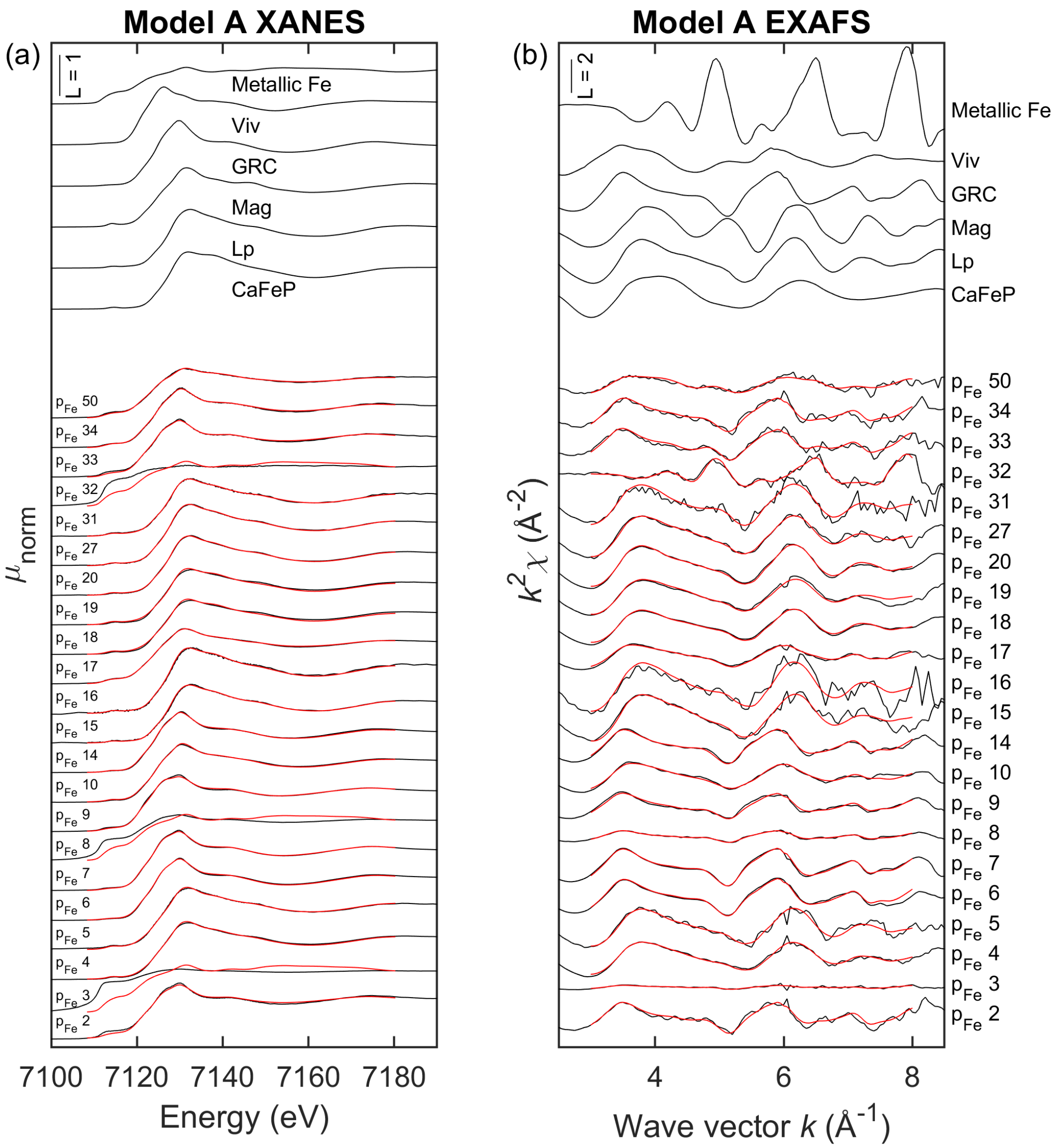

Figure S7: Model A. Experimental Fe *K*-edge (a) μ-XANES and (b) μ-EXAFS spectra (black lines) and linear combination reconstructions (red lines) from LCF results. Reference spectra are displayed. The corresponding fit results can be found in Table S5 (XANES) and Table S9 (EXAFS).

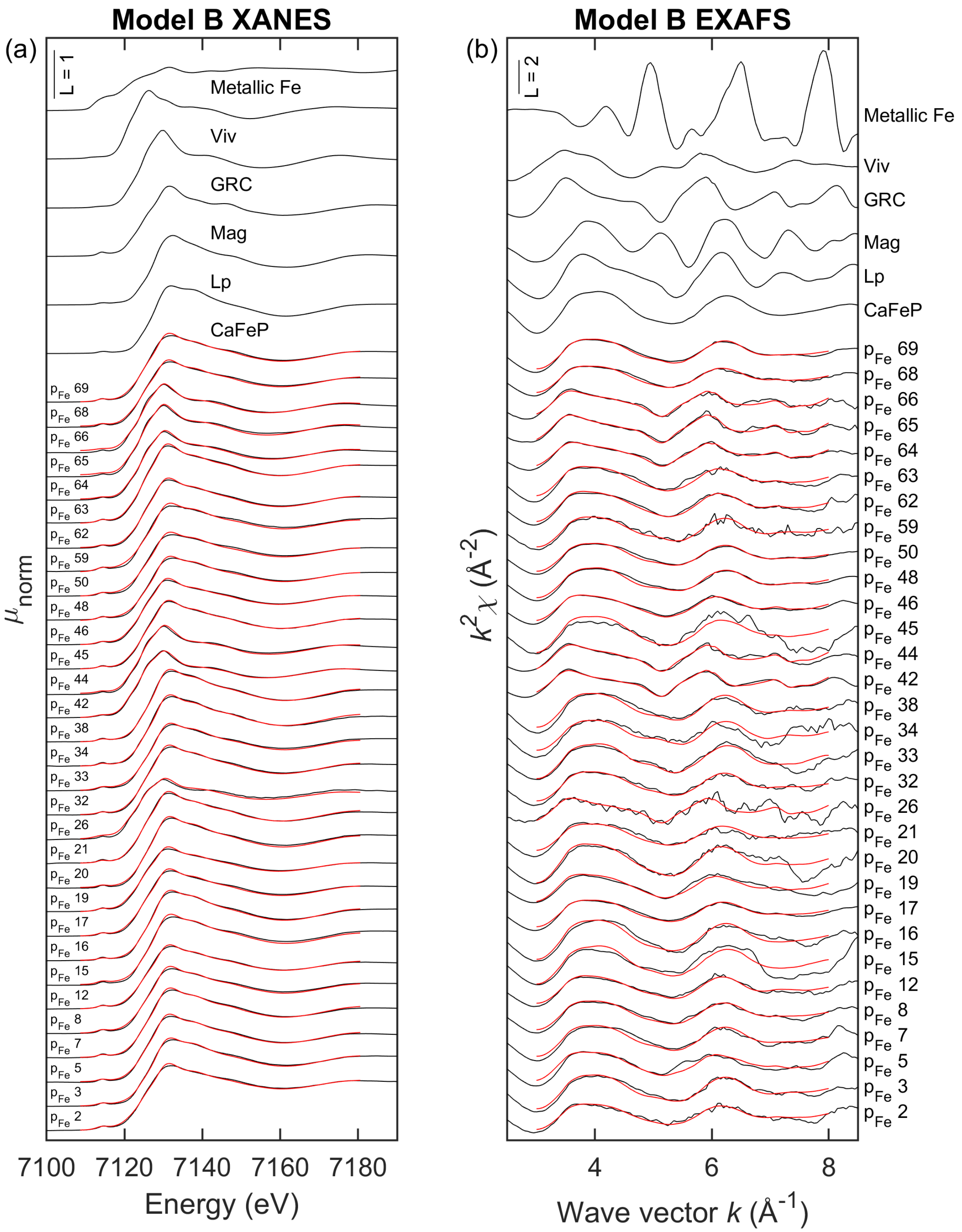

Figure S8: Model B. Recorded Fe *K*-edge (a) μ-XANES and (b) μ-EXAFS spectra (black lines) and linear combination reconstructions (red lines) from LCF results. Reference spectra are displayed. The corresponding fit results can be found in Table S6 (XANES) and Table S10 (EXAFS).

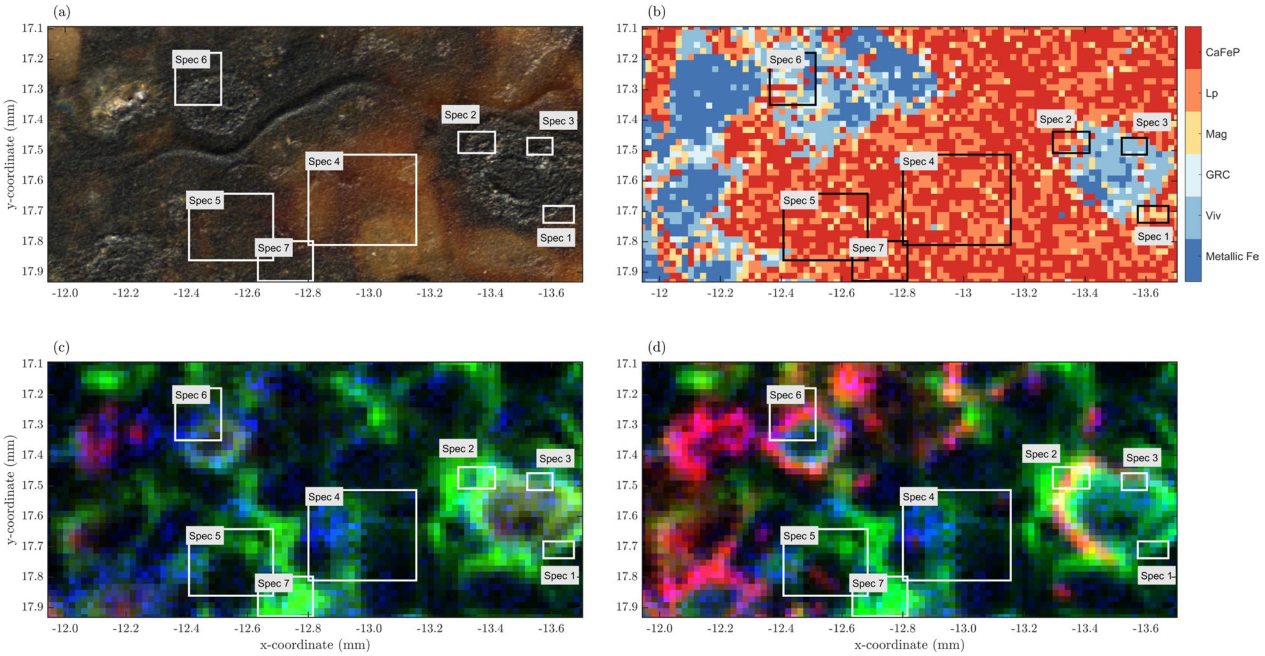

Figure S9: Model A. Chemical imaging results of area of interest 1. (a) Reflected light optical microscopy image of the area of interest 1, (b) chemical image, (c) Fe (red), As (green), P (blue) tri-color map and (d) Ca (red), As (green), P (blue) tricolor map. The corresponding data is displayed in Figure 4 in the main text. The frames labeled Spec 1 to 7 indicate areas over which an integrated XANES spectrum was calculated from the chemical imaging data. This data is displayed in Figure S10.

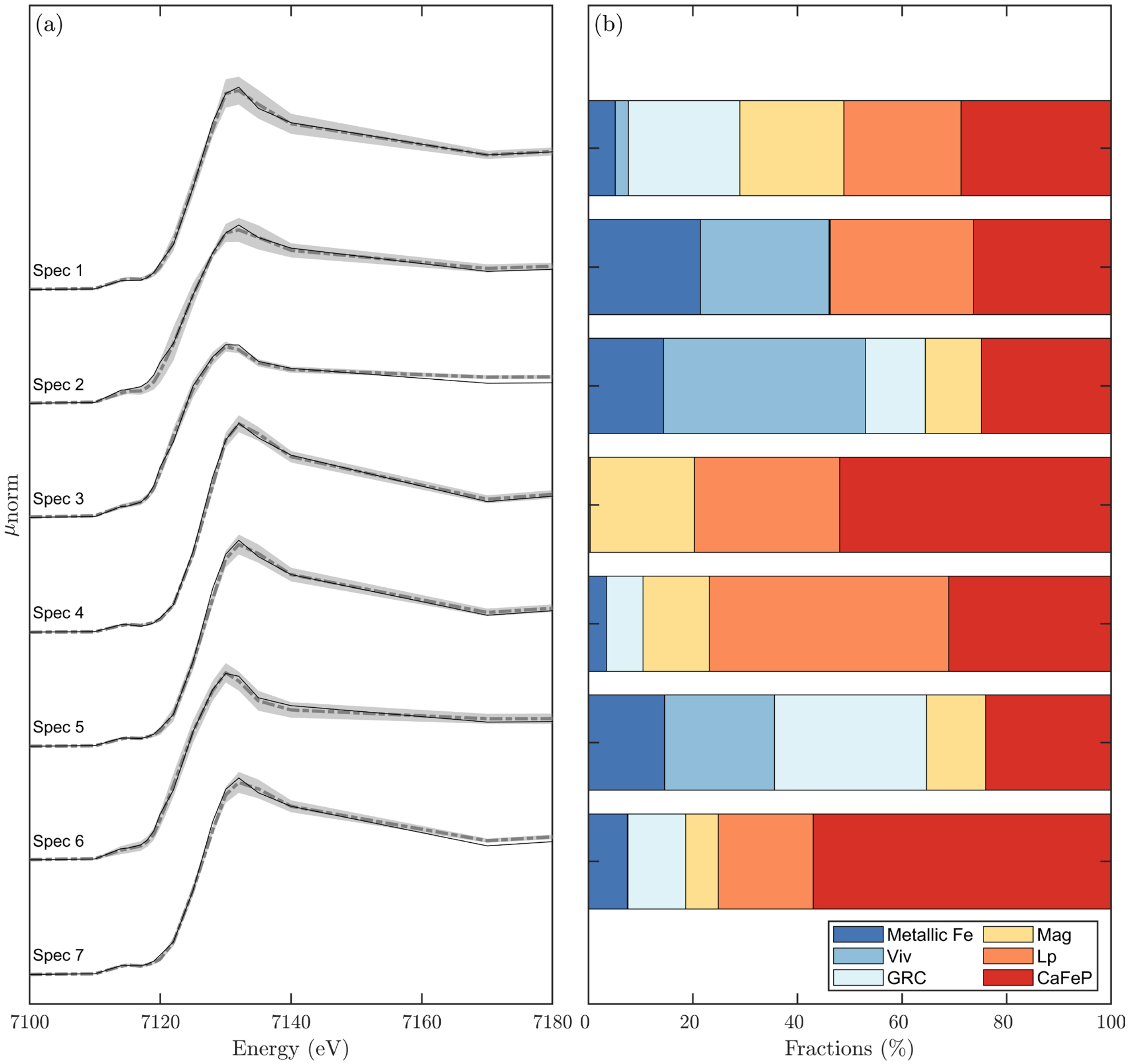

Figure S10: Model A. (a) Linear combination fit reconstructions (bold, black lines) of integrated chemical imaging data (dotted, grey lines) obtained from the area of interest 1. The shaded areas indicate two standard deviations at each energy value. (b) LCF fractions. For the locations of Spec 1 to 7 refer to Figure S9.

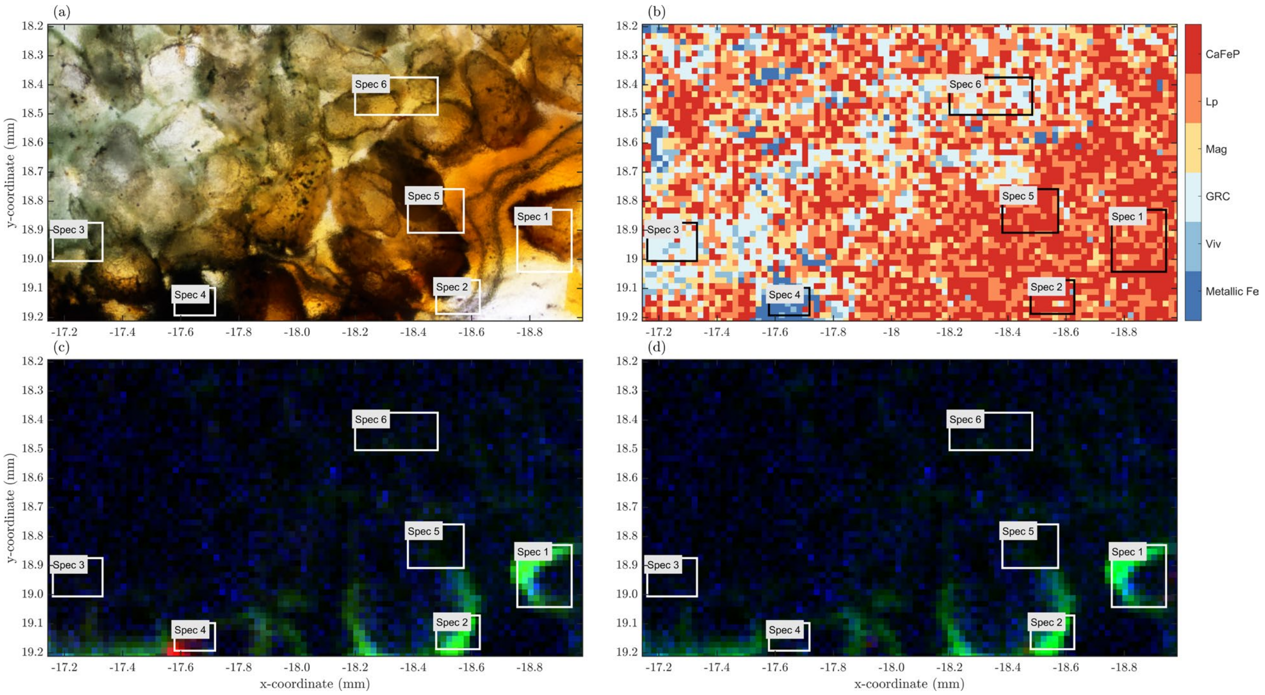

Figure S11: Model A. Chemical imaging results of the area of interest 2. (a) Transmitted light optical microscopy image of the area of interest, (b) chemical image, (c) Fe (red), As (green), P (blue) tri-color map and (d) Ca (red), As (green), P (blue) tricolor map. The corresponding data is displayed in Figure 4 in the main text. The frames labeled Spec 1 to 6 indicate locations in which an integrated XANES spectrum was calculated from the chemical imaging data. This data is displayed in Figure S12.

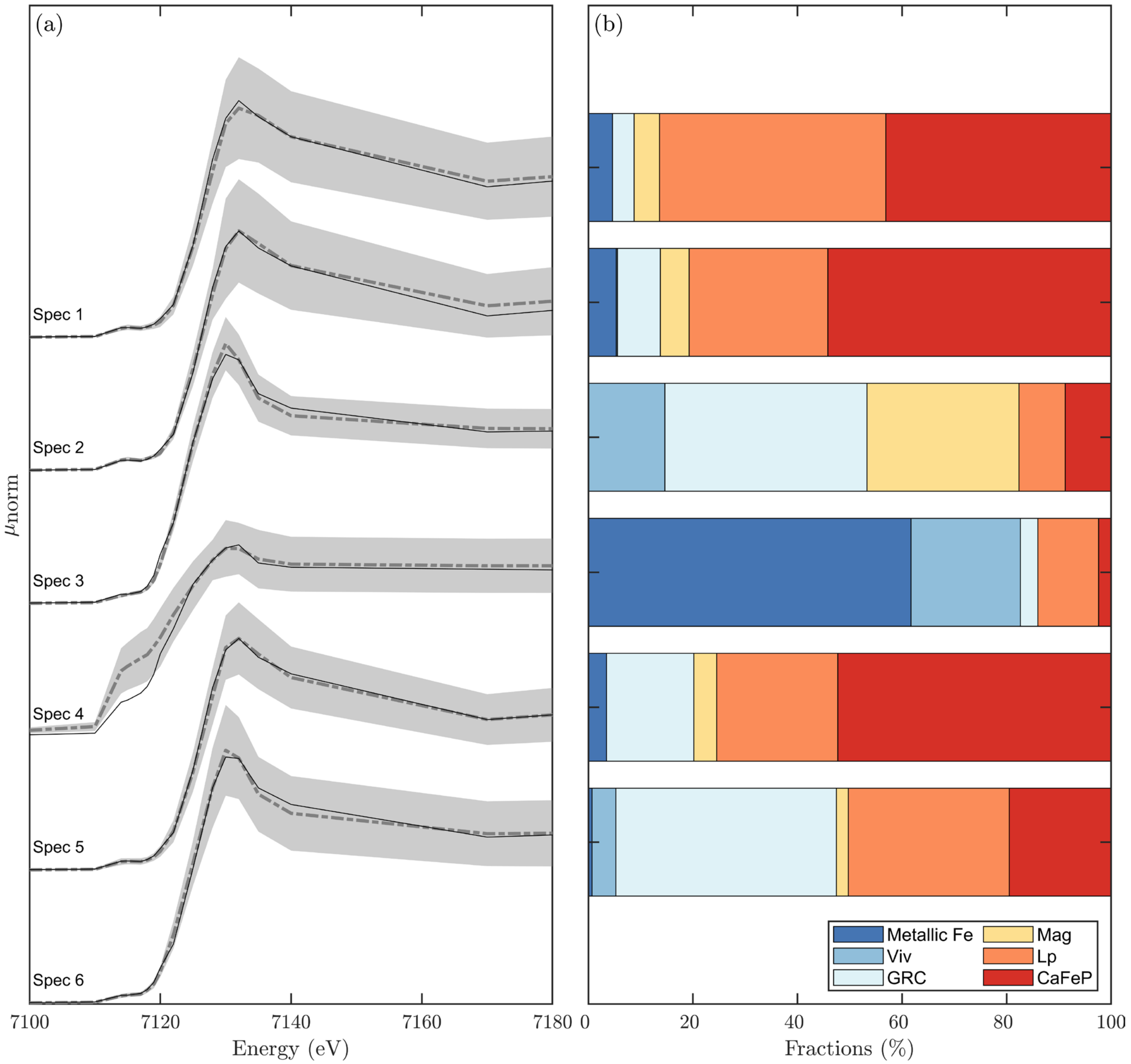

Figure S12: Model A. (a) linear combination fits (bold, black lines) to integrated chemical imaging data (dotted, grey lines) obtained from the area of interest 2. The shaded areas indicate two standard deviations at each energy value. (b) LCF fractions. For the locations of Spec 1 to 6 refer to Figure S11.

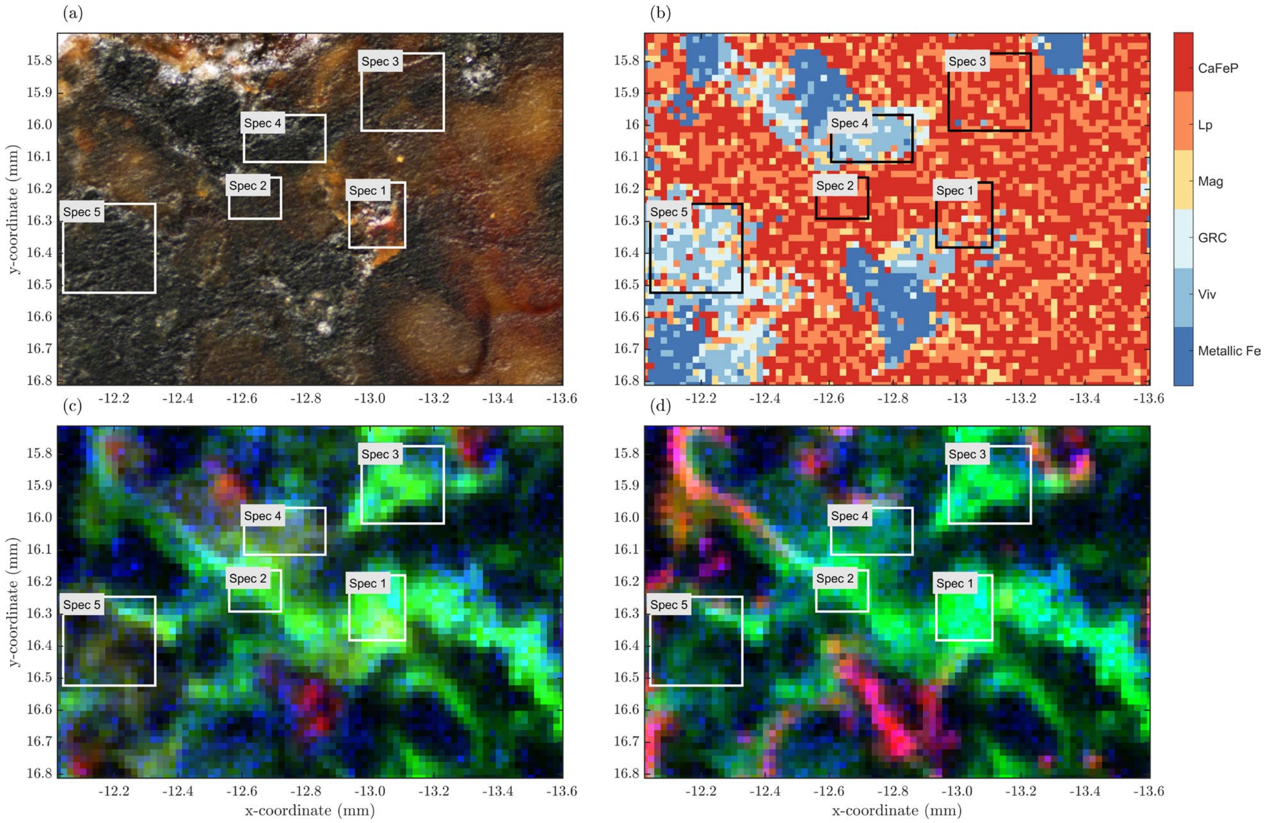

Figure S13: Model A. Chemical imaging results of the area of interest 3. (a) Reflected light optical microscopy image of the area of interest, (b) chemical image, (c) Fe (red), As (green), P (blue) tri-color map and (d) Ca (red), As (green), P (blue) tricolor map. The corresponding data is displayed in Figure 4 in the main text. The frames labeled Spec 1 to 5 indicate locations in which an integrated XANES spectrum was calculated from the chemical imaging data. This data is displayed in Figure S14.

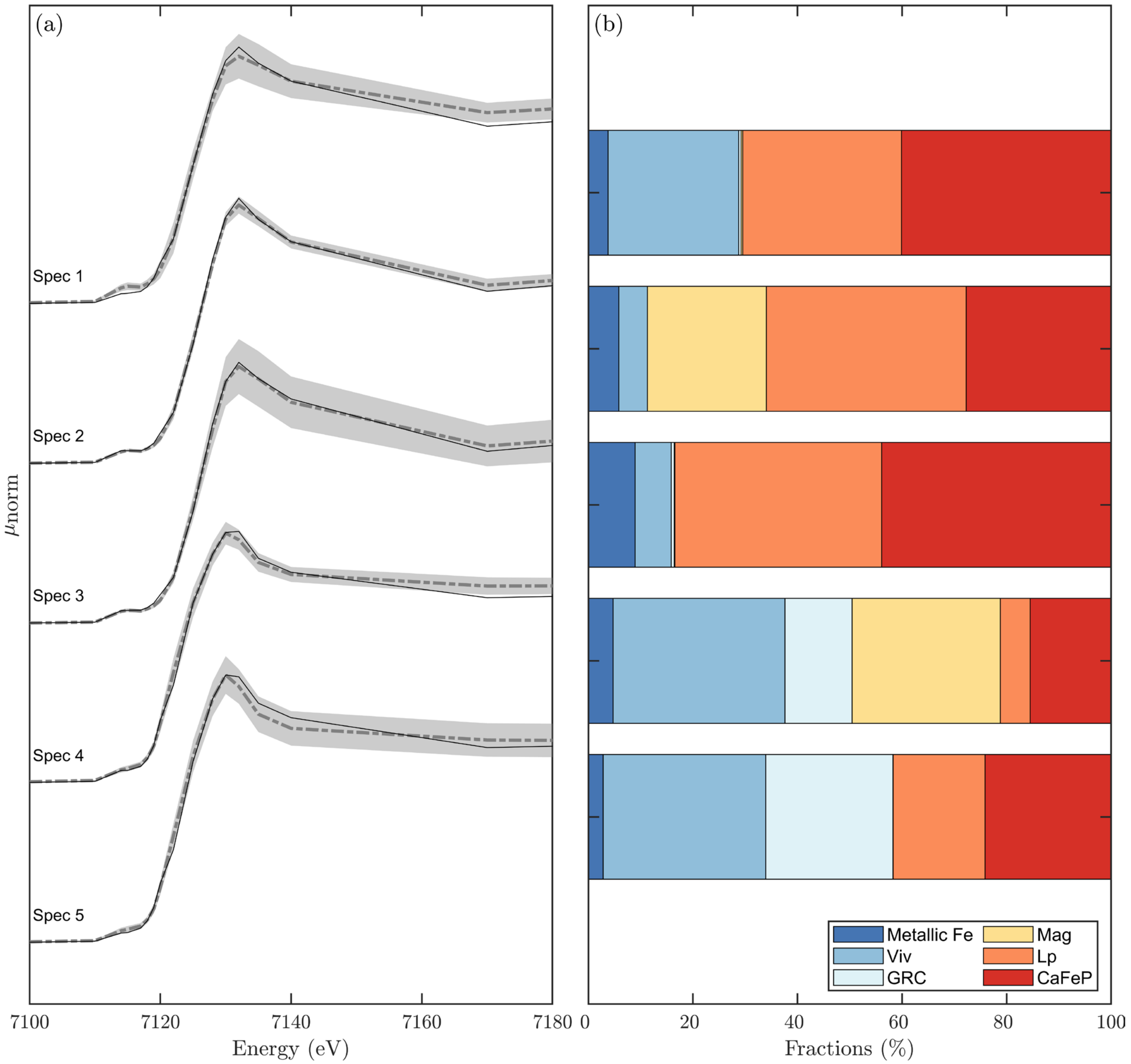

Figure S14: Model A. (a) linear combination fits (bold, black lines) to integrated chemical imaging data (dotted, grey lines) obtained from the area of interest 3. The shaded areas indicate two standard deviations at each energy value. (b) LCF fractions. For the locations of Spec 1 to 5 refer to Figure S13.

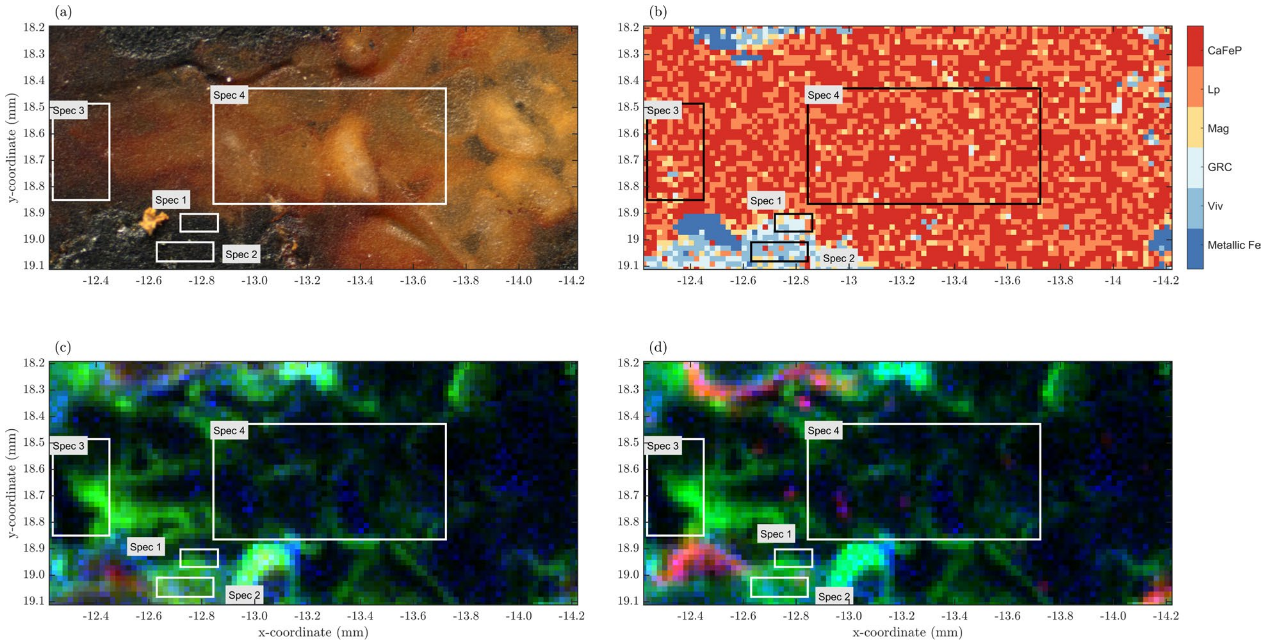

Figure S15: Model A. Chemical imaging results of the area of interest 4. (a) Reflected light optical microscopy image of the area of interest, (b) chemical image, (c) Fe (red), As (green), P (blue) tri-color map and (d) Ca (red), As (green), P (blue) tricolor map. The corresponding data is displayed in Figure 4 in the main text. The frames labeled Spec 1 to 4 indicate locations in which an integrated XANES spectrum was calculated from the chemical imaging data. This data is displayed in Figure S16.

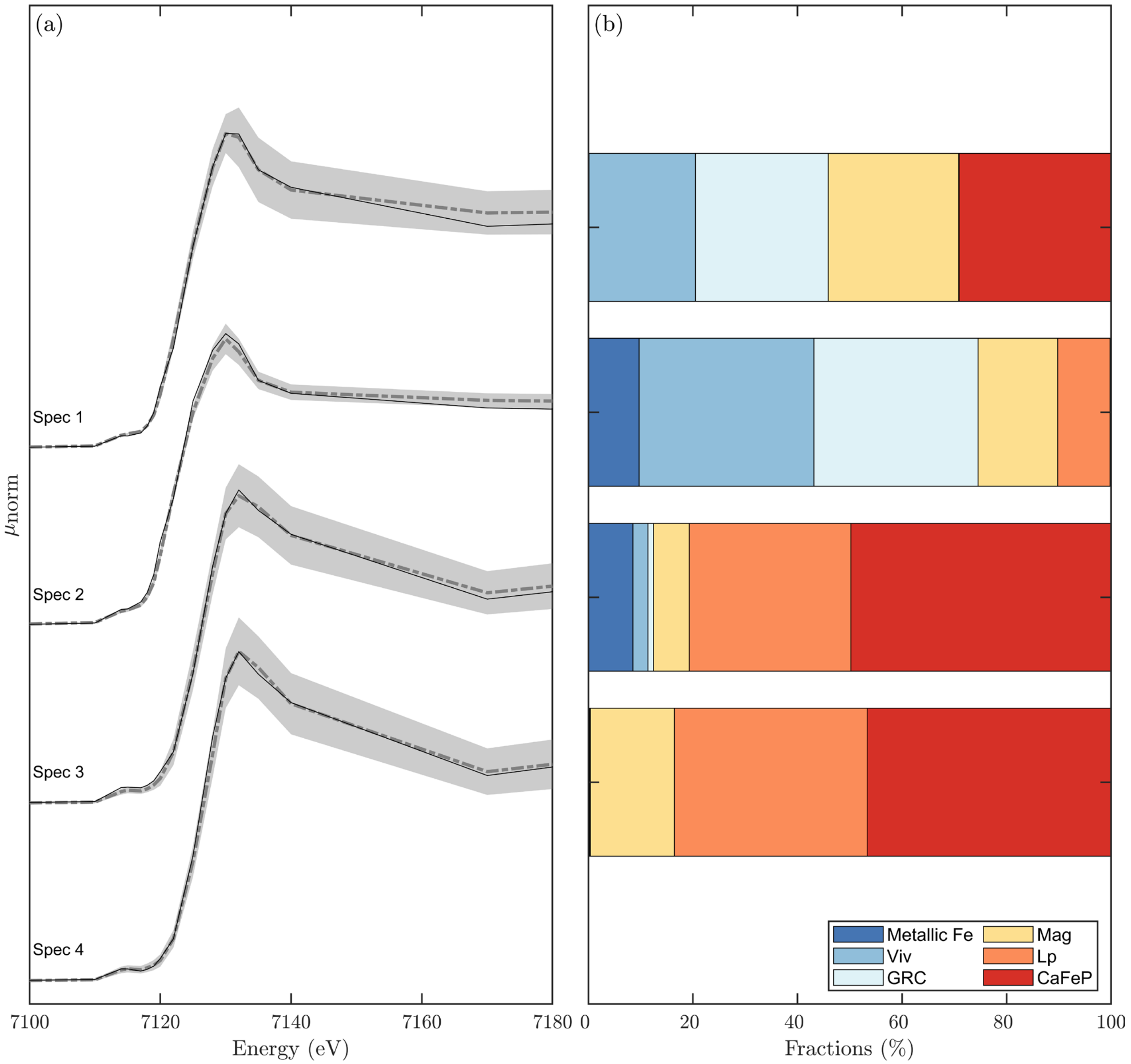

Figure S16: Model A. (a) linear combination fits (bold, black lines) to integrated chemical imaging data (dotted, grey lines) obtained from the area of interest 4. The shaded areas indicate two standard deviations at each energy value. (b) LCF fractions. For the locations of Spec 1 to 4 refer to Figure S15.

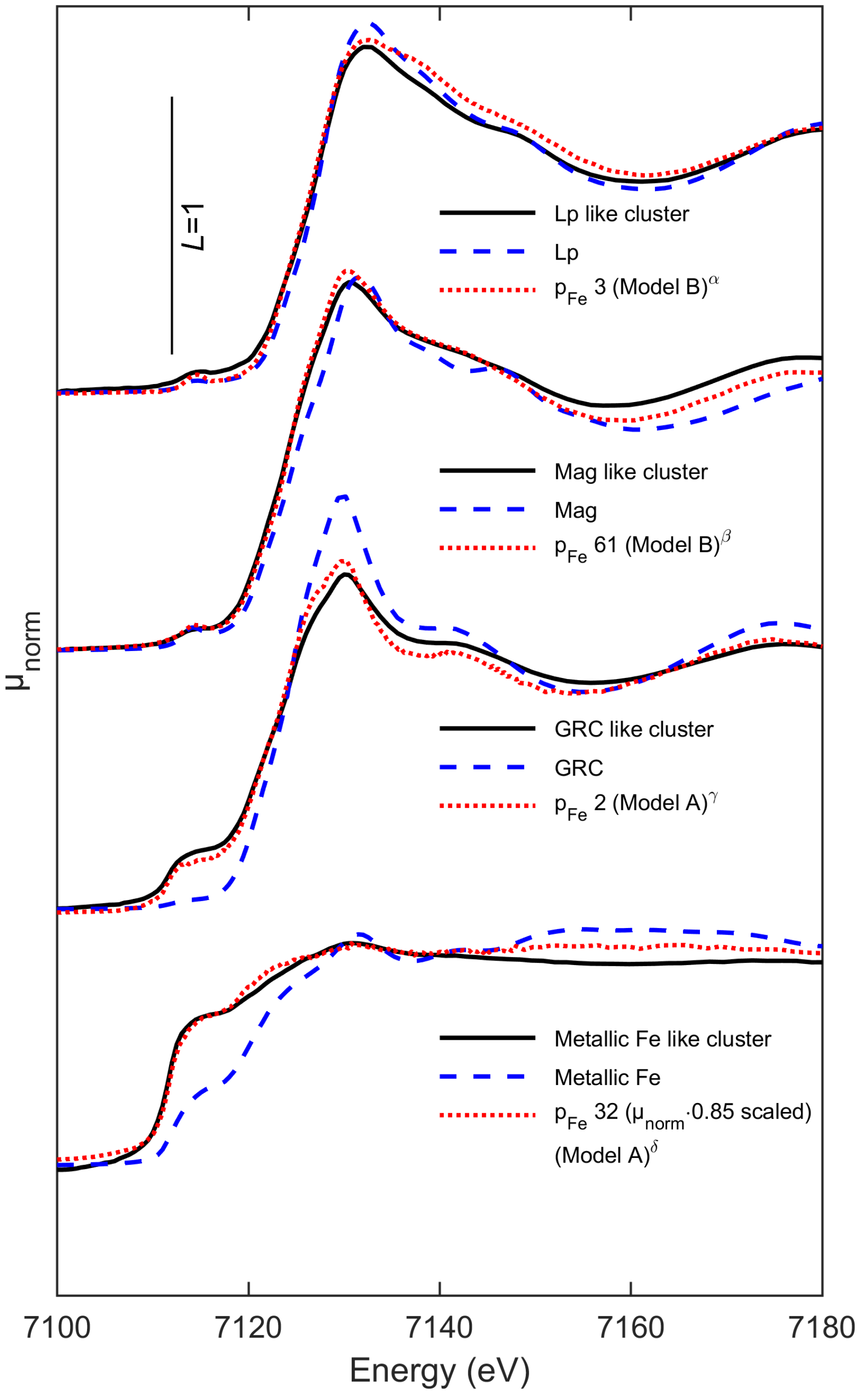

Figure S17: Model A. Full field data derived average XANES spectra in four clusters (bold, black lines). Based on their similarity to reference spectra, these clusters are referred to as Lp-like, Mag-like, GRC-like and metallic Fe-like (from top to bottom). In addition, the reference spectra used for the LCF analysis of μ-XANES spectra and chemical imaging data are displayed (dashed, blue curves). To relate the full field cluster spectra that suffer from spectral compression (due to locally enhanced absorber concentrations) to the μ-XANES data, selected μ-XANES spectra are displayed (dotted, red curves). For a discussion of the presented data, see section S2, paragraph **Evaluation of full field Fe XANES data**.

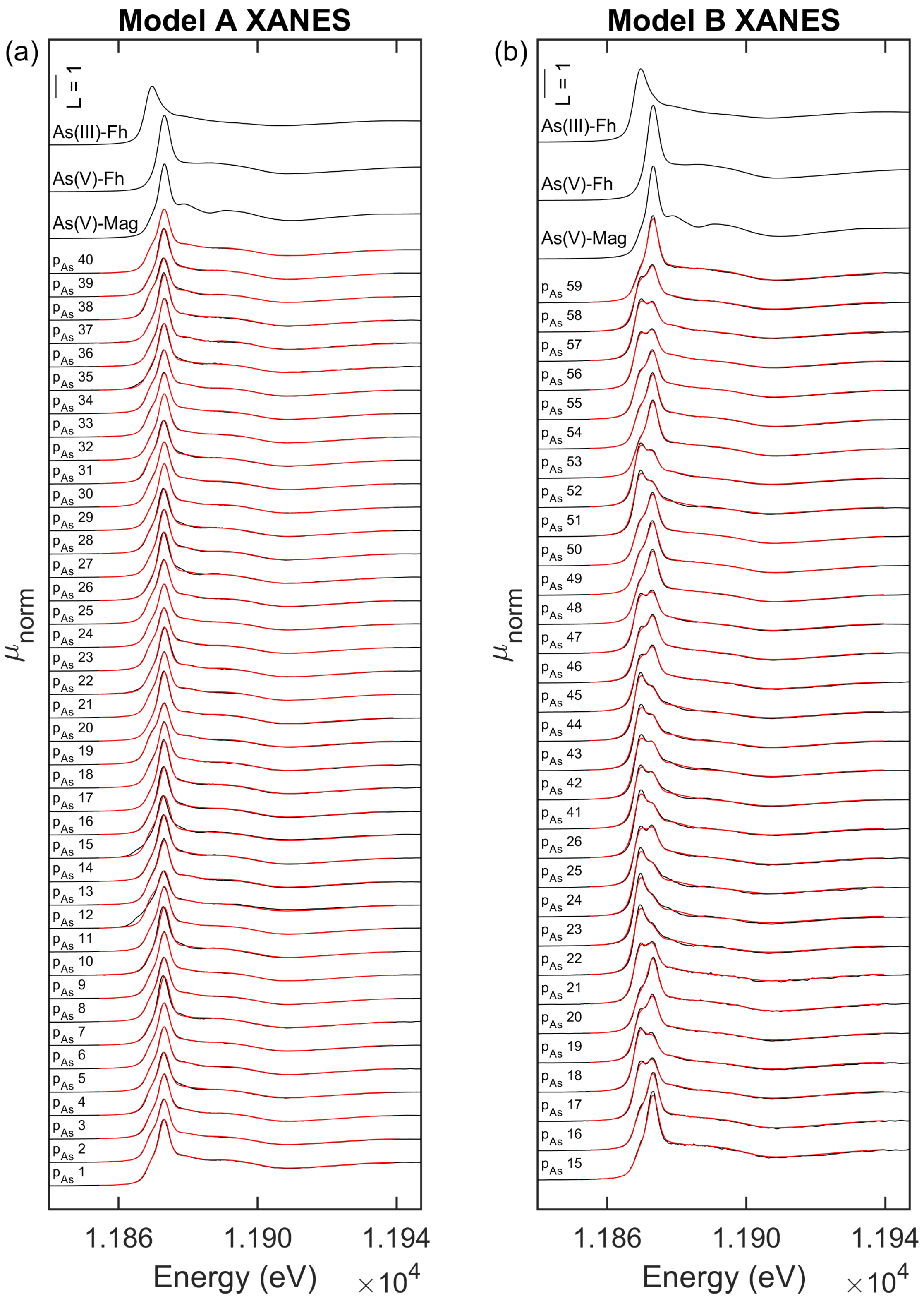

Figure S18: Models A and B. Recorded As K-edge μ-XANES spectra (black lines) of Models A (a) and B (b) and linear combination reconstructions (red lines) from LCF results. Reference spectra are displayed. The corresponding fit results can be found in Tables S7 (Model A) and S8 (Model B).

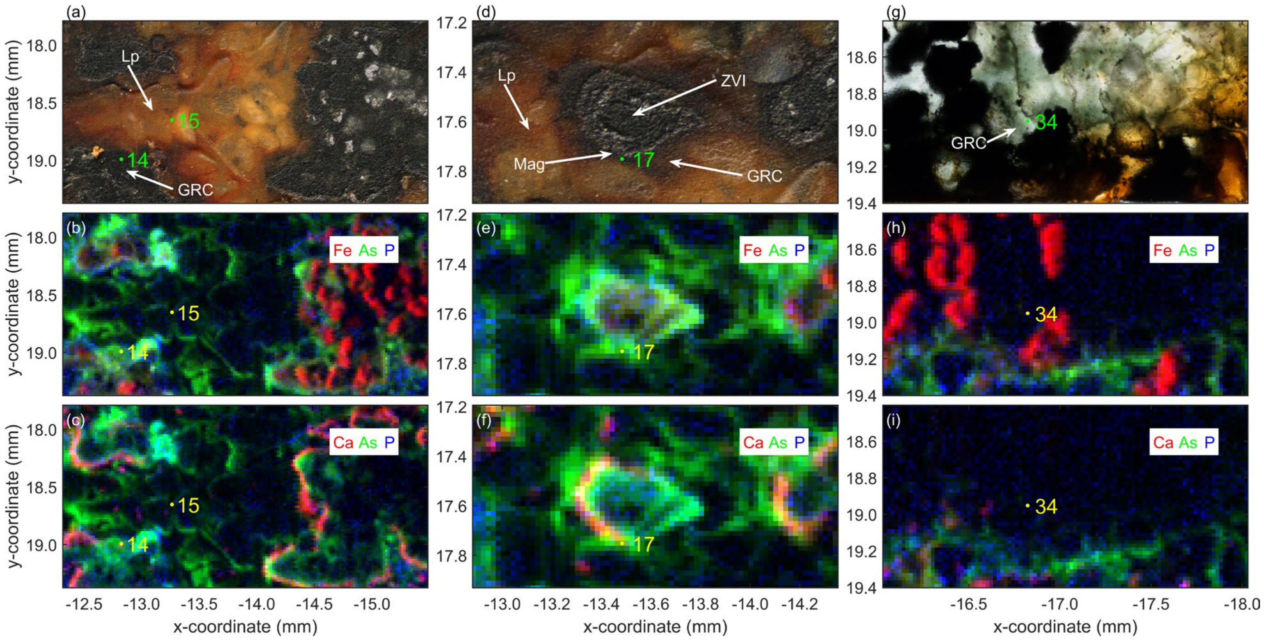

Figure S19. Model A. (a, d) Reflected or (g) transmitted light optical microscopy images of three representative locations displayed in Figure 4. (b,e,h) Tricolor maps for Fe-As-P and (c,i,h) Ca-As-P. This data corresponds to the data shown in Figure 4c,d. XANES and EXAFS spectra measured on the locations indicated in (a,d,g) are shown in Figure S20.

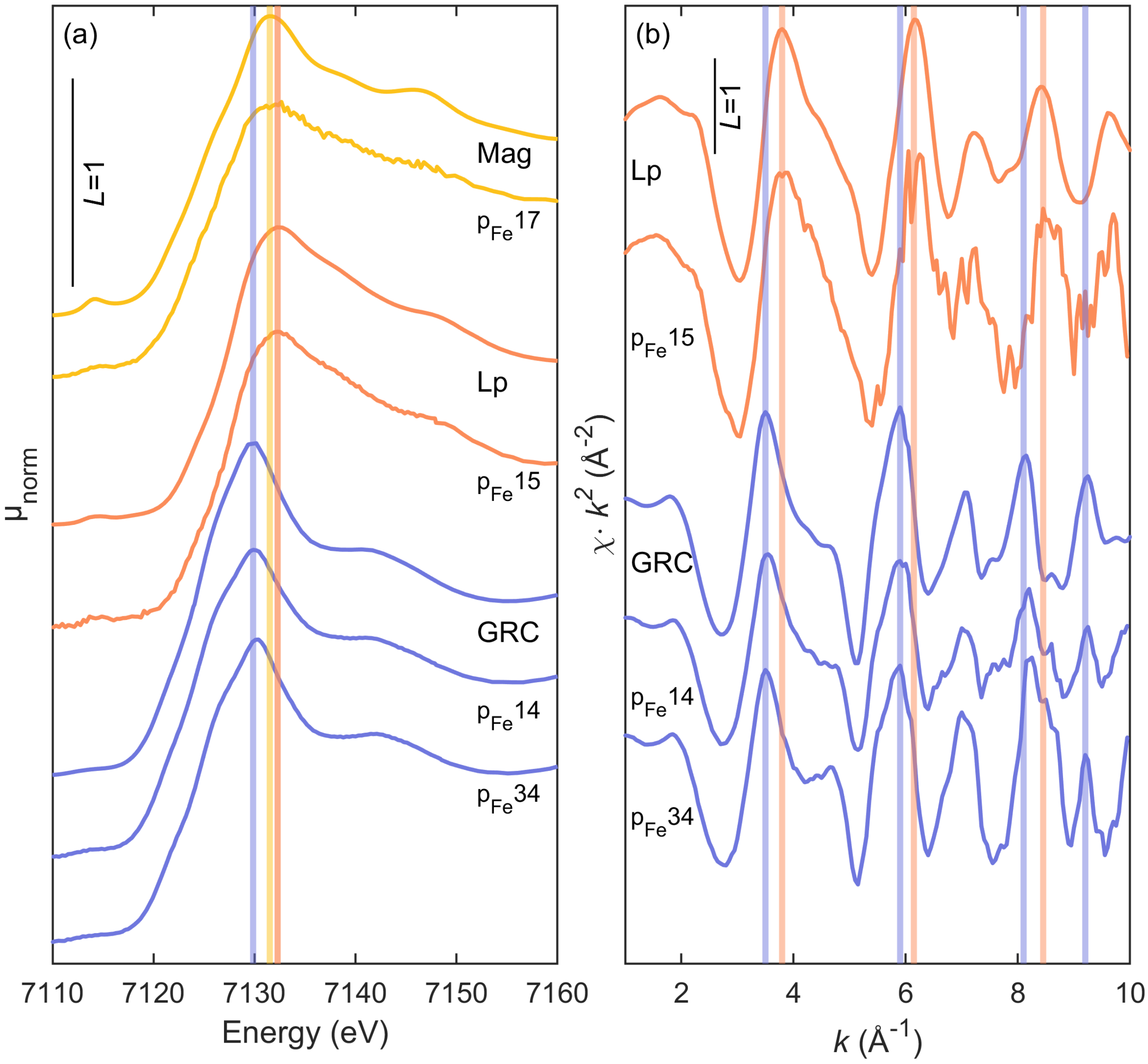

Figure S20: Model A. Fe *K*-edge (a) XANES and (b) EXAFS data in locations p_{Fe} 14, 15, 17 and 34. The spectrum p_{Fe} 17 was recorded in fluorescence mode, the other spectra in transmission mode. The optical and μ-XRF results corresponding to these spectra are shown in Figure S19.

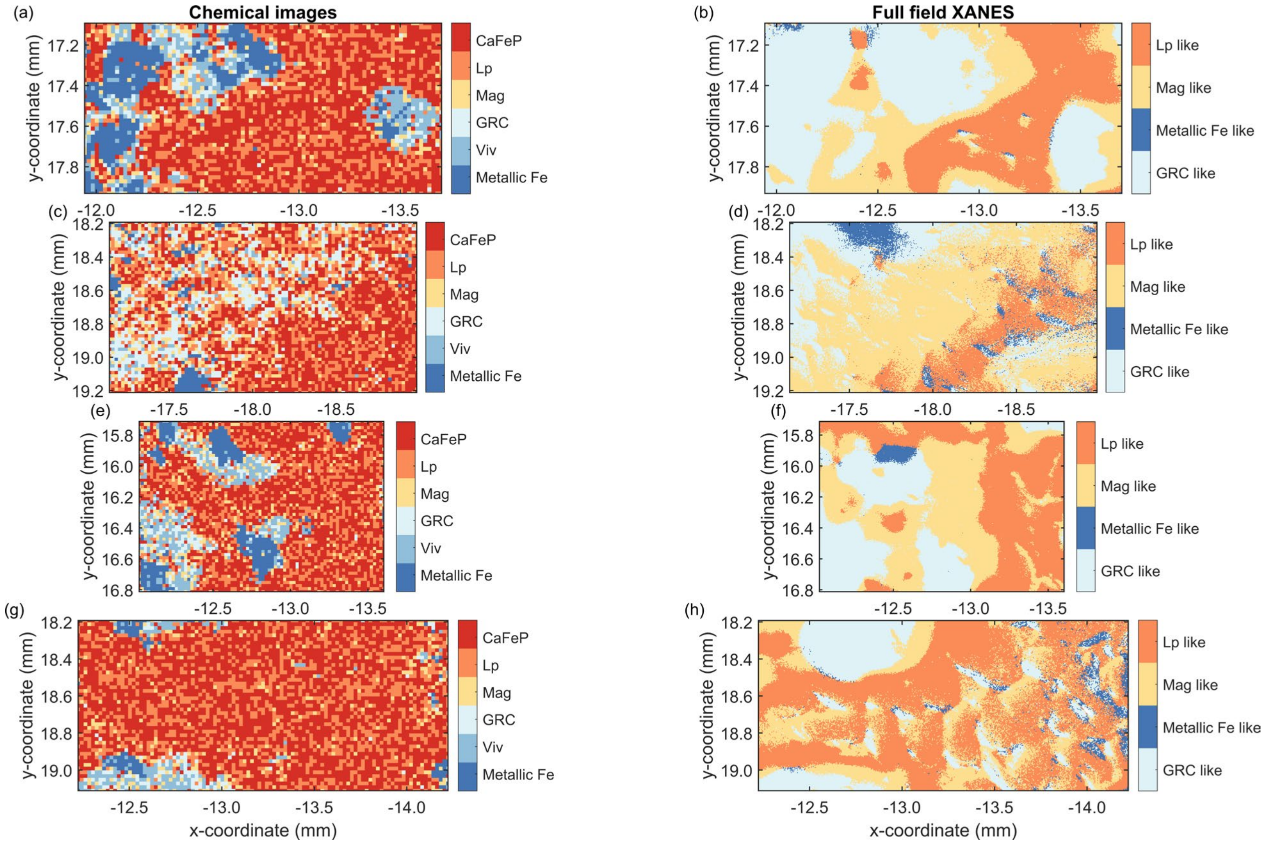

Figure S21: Model A. Chemical images (left column) and full field XANES data (right column) in the areas of interest 1 to 4 from top to bottom.

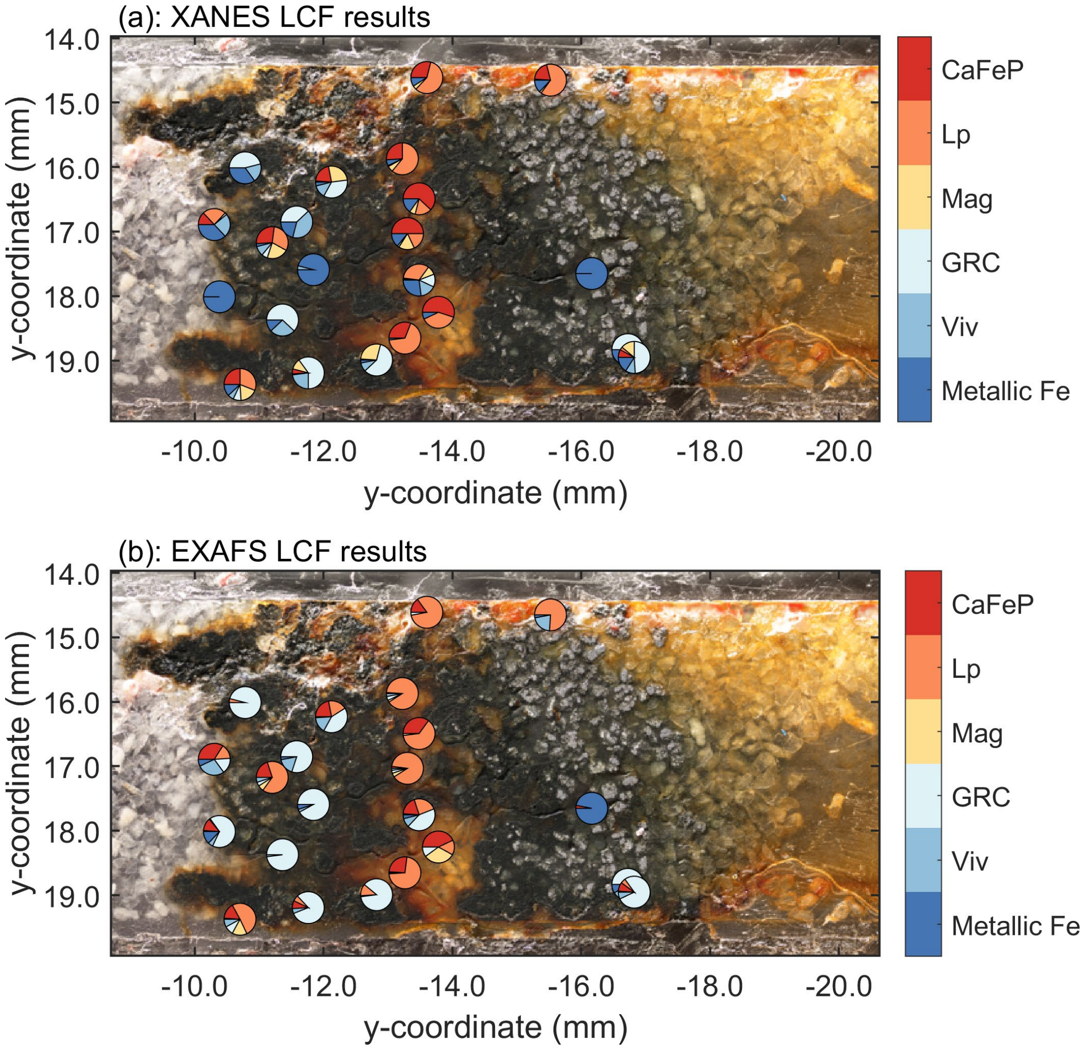

Figure S22: Model A. Comparison of Fe *K*-edge (a) XANES (Table S5) and (b) EXAFS LCF results (Table S9). Significant differences between the results displayed in (a) and (b) arise from the quality of the LCF as discussed in the section on Fe and As speciation in the main text.

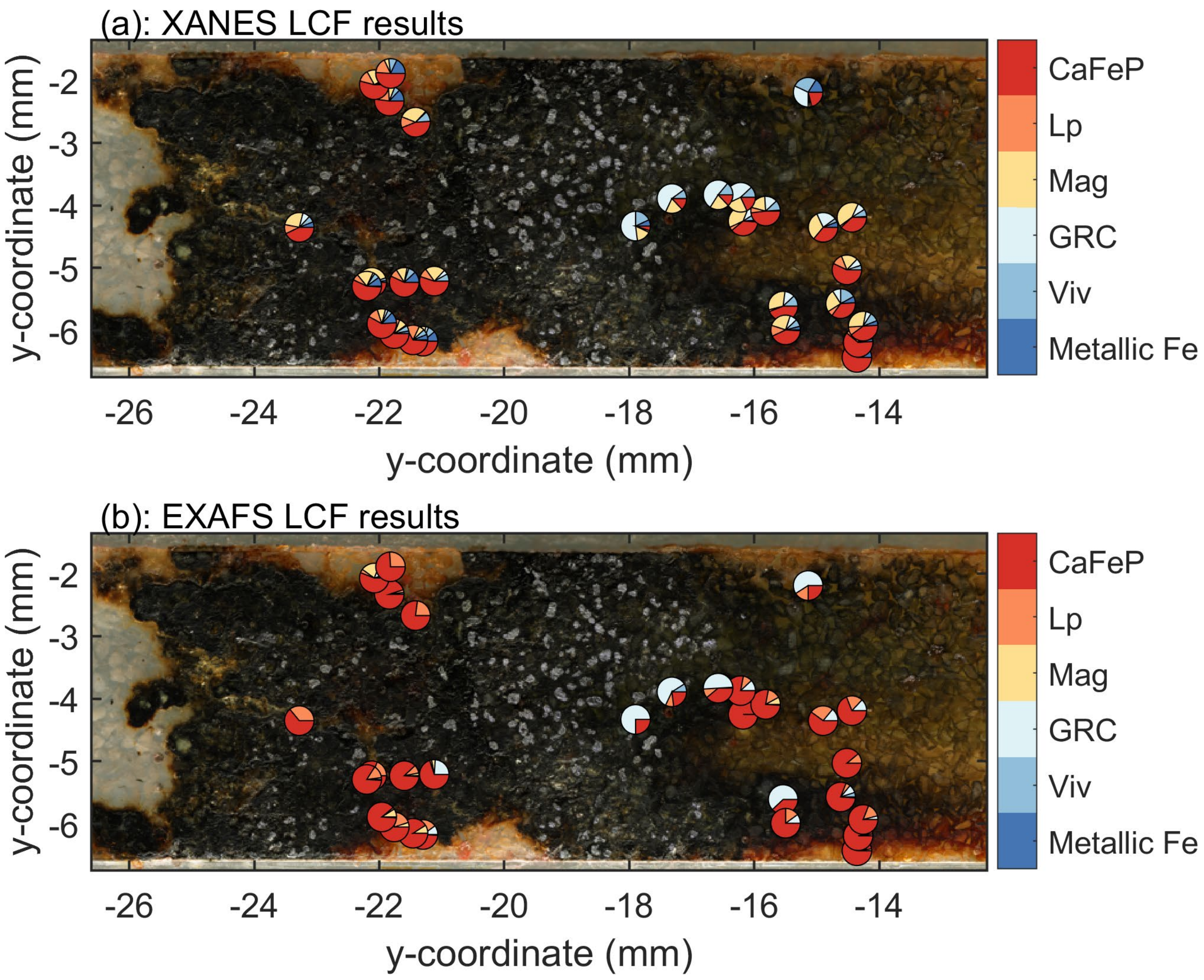

Figure S23: Model B. Comparison of Fe *K*-edge (a) XANES (Table S6) and (b) EXAFS LCF results (Table S10). Significant differences between the results displayed in (a) and (b) arise from the quality of the LCF as discussed in the section on Fe and As speciation in the main text.

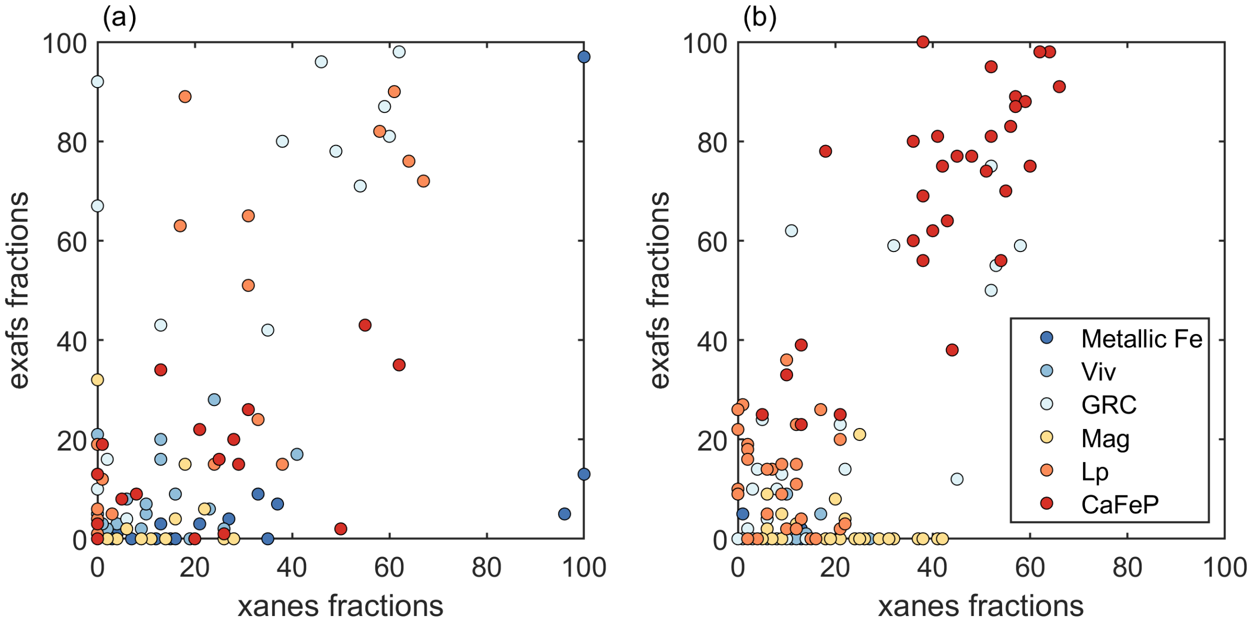

Figure S24: Models A and B. Fe *K*-edge XANES *versus* EXAFS based LCF result for (a) Model A and (b) Model B. Data was taken from Table S5, Table S6, Table S9 and Table S10.

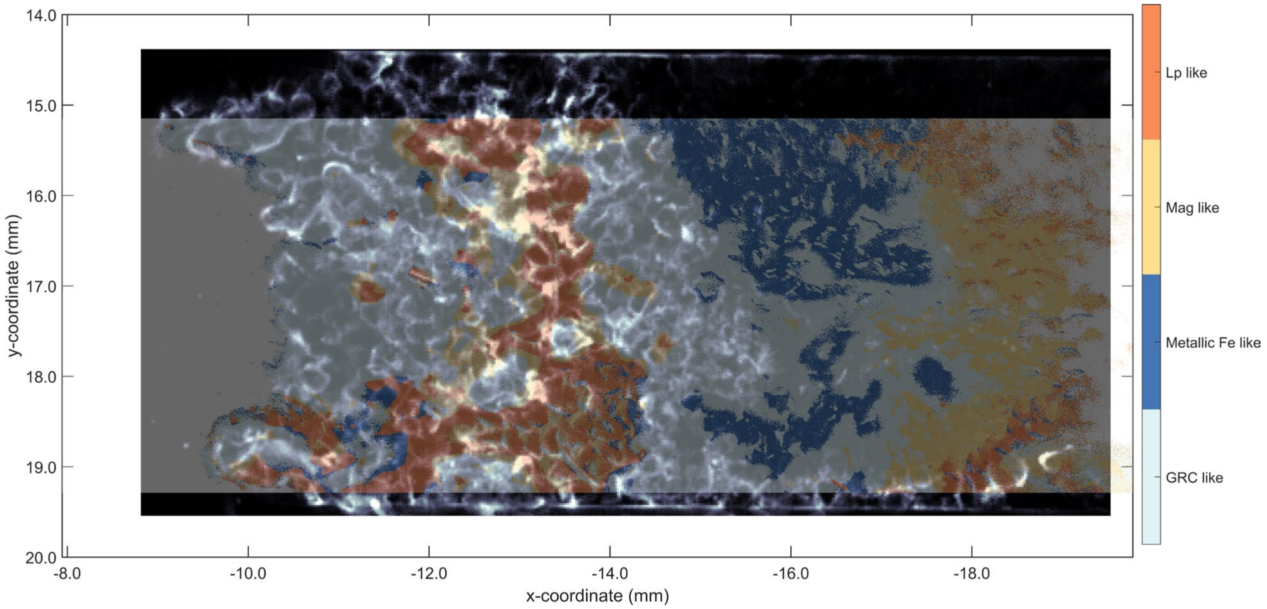

Figure S25: Model A. Overlay plot of As distribution map (black to white) and Fe full field XANES results (colors indicated in the colorbar on the right).

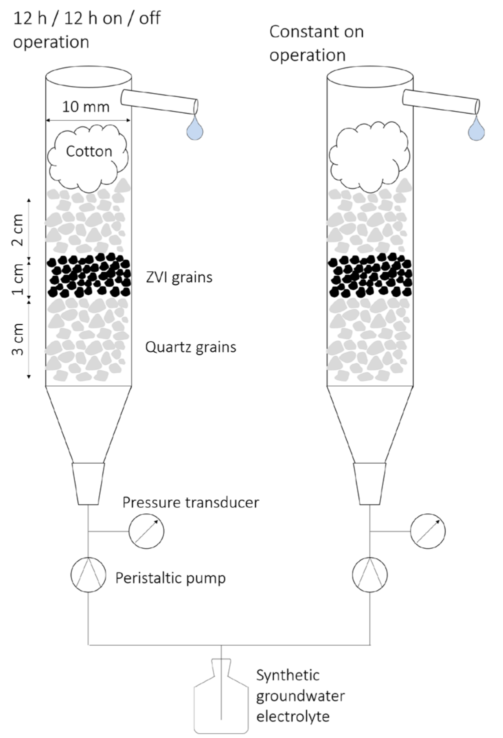

Figure S26: The laboratory column setup used to determine the head loss during constant column operation.

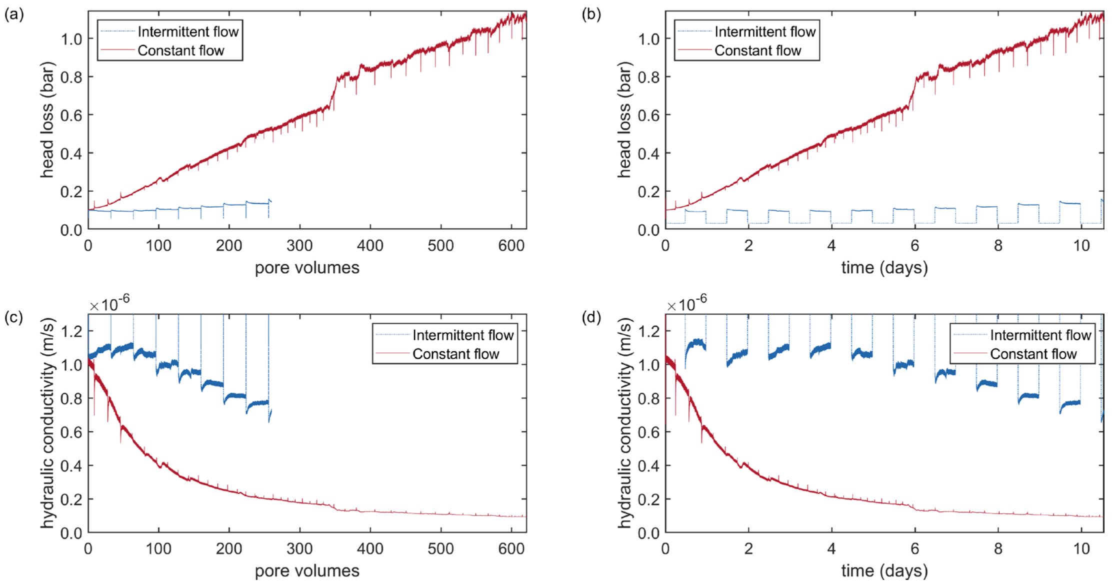

Figure S27: Head loss as a function of (a) filtered bed volumes and (b) experimental run time. The dashed, blue and bold, orange curves represent the head losses measured for the constantly and intermittently operated column, respectively. The head loss data is used to calculate the hydraulic conductivity, displayed as (c) a function of bed volumes and (d) column operation time.

REFERNCES

1. Abedin, M. J.; Cresser, M. S.; Meharg, A. A.; Feldmann, J.; Cotter-Howells, J., Arsenic Accumulation and Metabolism in Rice (Oryza sativa L.). *Environmental Science & Technology* **2002,** *36*, (5), 962-968.
2. Meharg, A. A.; Rahman, M. M., Arsenic Contamination of Bangladesh Paddy Field Soils: Implications for Rice Contribution to Arsenic Consumption. *Environmental Science & Technology* **2003,** *37*, (2), 229-234.
3. Hug, S. J.; Winkel, L. H. E.; Voegelin, A.; Berg, M.; Johnson, A. C., Arsenic and Other Geogenic Contaminants in Groundwater - A Global Challenge. *CHIMIA International Journal for Chemistry* **2020,** *74*, (7-8), 524-537.
4. Hering, J. G.; Katsoyiannis, I. A.; Theoduloz, G. A.; Berg, M.; Hug, S. J., Arsenic Removal from Drinking Water: Experiences with Technologies and Constraints in Practice. *Journal of Environmental Engineering* **2017,** *143*, (5), 03117002.
5. Podgorski, J.; Berg, M., Global threat of arsenic in groundwater. *Science* **2020,** *368*, (6493), 845-850.
6. Berg, M.; Luzi, S.; Trang, P. T. K.; Viet, P. H.; Giger, W.; Stüben, D., Arsenic Removal from Groundwater by Household Sand Filters: Comparative Field Study, Model Calculations, and Health Benefits. *Environmental Science & Technology* **2006,** *40*, (17), 5567-5573.
7. Nitzsche, K. S.; Lan, V. M.; Trang, P. T. K.; Viet, P. H.; Berg, M.; Voegelin, A.; Planer-Friedrich, B.; Zahoransky, J.; Müller, S.-K.; Byrne, J. M.; Schröder, C.; Behrens, S.; Kappler, A., Arsenic removal from drinking water by a household sand filter in Vietnam — Effect of filter usage practices on arsenic removal efficiency and microbiological water quality. *Science of The Total Environment* **2015,** *502*, 526-536.
8. Voegelin, A.; Kaegi, R.; Berg, M.; Nitzsche, K. S.; Kappler, A.; Lan, V. M.; Trang, P. T. K.; Göttlicher, J.; Steininger, R., Solid-phase characterisation of an effective household sand filter for As, Fe and Mn removal from groundwater in Vietnam. *Environmental Chemistry* **2014,** *11*, (5), 566-578.
9. Hug, S. J.; Leupin, O. X.; Berg, M., Bangladesh and Vietnam: Different Groundwater Compositions Require Different Approaches to Arsenic Mitigation. *Environmental Science & Technology* **2008,** *42*, (17), 6318-6323.
10. Mueller, B.; Hug, S. J., Climatic variations and de-coupling between arsenic and iron in arsenic contaminated ground water in the lowlands of Nepal. *Chemosphere* **2018,** *210*, 347-358.
11. Bretzler, A.; Nikiema, J.; Lalanne, F.; Hoffmann, L.; Biswakarma, J.; Siebenaller, L.; Demange, D.; Schirmer, M.; Hug, S. J., Arsenic removal with zero-valent iron filters in Burkina Faso: Field and laboratory insights. *Science of The Total Environment* **2020,** *737*, 139466.
12. Dixit, S.; Hering, J. G., Comparison of Arsenic(V) and Arsenic(III) Sorption onto Iron Oxide Minerals: Implications for Arsenic Mobility. *Environmental Science & Technology* **2003,** *37*, (18), 4182-4189.
13. Hussam, A.; Munir, A. K. M., A simple and effective arsenic filter based on composite iron matrix: Development and deployment studies for groundwater of Bangladesh. *Journal of Environmental Science and Health, Part A* **2007,** *42*, (12), 1869-1878.
14. Neumann, A.; Kaegi, R.; Voegelin, A.; Hussam, A.; Munir, A. K. M.; Hug, S. J., Arsenic Removal with Composite Iron Matrix Filters in Bangladesh: A Field and Laboratory Study. *Environmental Science & Technology* **2013,** *47*, (9), 4544-4554.
15. Fu, F.; Dionysiou, D. D.; Liu, H., The use of zero-valent iron for groundwater remediation and wastewater treatment: A review. *Journal of Hazardous Materials* **2014,** *267*, 194-205.
16. Su, C.; Puls, R. W., In Situ Remediation of Arsenic in Simulated Groundwater Using Zerovalent Iron: Laboratory Column Tests on Combined Effects of Phosphate and Silicate. *Environmental Science & Technology* **2003,** *37*, (11), 2582-2587.
17. Phenrat, T.; Thongboot, T.; Lowry, G. V., Electromagnetic Induction of Zerovalent Iron (ZVI) Powder and Nanoscale Zerovalent Iron (NZVI) Particles Enhances Dechlorination of Trichloroethylene in

Contaminated Groundwater and Soil: Proof of Concept. *Environmental Science & Technology* **2016,** *50*, (2), 872-880.
18. Johnson, R. L.; Thoms, R. B.; O'Brien Johnson, R.; Krug, T., Field Evidence for Flow Reduction through a Zero-Valent Iron Permeable Reactive Barrier. *Groundwater Monitoring & Remediation* **2008,** *28*, (3), 47-55.
19. Legrand, L.; Mazerolles, L.; Chaussé, A., The oxidation of carbonate green rust into ferric phases:solid-state reaction or transformation via solution. *Geochimica et Cosmochimica Acta* **2004,** *68*, (17), 3497-3507.
20. Su, C.; Puls, R. W., Significance of Iron(II,III) Hydroxycarbonate Green Rust in Arsenic Remediation Using Zerovalent Iron in Laboratory Column Tests. *Environmental Science & Technology* **2004,** *38*, (19), 5224-5231.
21. Stampfl, P. P., Ein basisches eisen-II-III-karbonat in rost. *Corrosion Science* **1969,** *9*, (3), 185-187.
22. Trolard, F.; Génin, J. M. R.; Abdelmoula, M.; Bourrié, G.; Humbert, B.; Herbillon, A., Identification of a green rust mineral in a reductomorphic soil by Mossbauer and Raman spectroscopies. *Geochimica et Cosmochimica Acta* **1997,** *61*, (5), 1107-1111.
23. Feder, F.; Trolard, F.; Klingelhöfer, G.; Bourrié, G., In situ Mössbauer spectroscopy: Evidence for green rust (fougerite) in a gleysol and its mineralogical transformations with time and depth. *Geochimica et Cosmochimica Acta* **2005,** *69*, (18), 4463-4483.
24. Christiansen, B. C.; Balic-Zunic, T.; Dideriksen, K.; Stipp, S. L. S., Identification of Green Rust in Groundwater. *Environmental Science & Technology* **2009,** *43*, (10), 3436-3441.
25. Wang, Y.; Morin, G.; Ona-Nguema, G.; Brown, G. E., Arsenic(III) and Arsenic(V) Speciation during Transformation of Lepidocrocite to Magnetite. *Environmental Science & Technology* **2014,** *48*, (24), 14282-14290.
26. Perez, J. P. H.; Freeman, H. M.; Schuessler, J. A.; Benning, L. G., The interfacial reactivity of arsenic species with green rust sulfate (GRSO4). *Science of The Total Environment* **2019,** *648*, 1161-1170.
27. Perez, J. P. H.; Schiefler, A. A.; Rubio, S. N.; Reischer, M.; Overheu, N. D.; Benning, L. G.; Tobler, D. J., Arsenic removal from natural groundwater using 'green rust': Solid phase stability and contaminant fate. *Journal of Hazardous Materials* **2021,** *401*, 123327.
28. Perez, J. P. H.; Tobler, D. J.; Freeman, H. M.; Brown, A. P.; Hondow, N. S.; van Genuchten, C. M.; Benning, L. G., Arsenic species delay structural ordering during green rust sulfate crystallization from ferrihydrite. *Environmental Science: Nano* **2021**.
29. van Genuchten, C. M.; Behrends, T.; Dideriksen, K., Emerging investigator series: interdependency of green rust transformation and the partitioning and binding mode of arsenic. *Environmental Science: Processes & Impacts* **2019,** *21*, (9), 1459-1476.
30. Jönsson, J.; Sherman, D. M., Sorption of As(III) and As(V) to siderite, green rust (fougerite) and magnetite: Implications for arsenic release in anoxic groundwaters. *Chemical Geology* **2008,** *255*, (1), 173-181.
31. Randall, S. R.; Sherman, D. M.; Ragnarsdottir, K. V., Sorption of As(V) on green rust (Fe4(II)Fe2(III)(OH)12SO4 · 3H2O) and lepidocrocite (γ-FeOOH): Surface complexes from EXAFS spectroscopy. *Geochimica et Cosmochimica Acta* **2001,** *65*, (7), 1015-1023.
32. Benali, O.; Abdelmoula, M.; Refait, P.; Génin, J.-M. R., Effect of orthophosphate on the oxidation products of Fe(II)-Fe(III) hydroxycarbonate: the transformation of green rust to ferrihydrite. *Geochimica et Cosmochimica Acta* **2001,** *65*, (11), 1715-1726.
33. Feng, X.; Wang, X.; Zhu, M.; Koopal, L. K.; Xu, H.; Wang, Y.; Liu, F., Effects of phosphate and silicate on the transformation of hydroxycarbonate green rust to ferric oxyhydroxides. *Geochimica et Cosmochimica Acta* **2015,** *171*, 1-14.
34. Dubrawski, K. L.; van Genuchten, C. M.; Delaire, C.; Amrose, S. E.; Gadgil, A. J.; Mohseni, M., Production and Transformation of Mixed-Valent Nanoparticles Generated by Fe(0) Electrocoagulation. *Environmental Science & Technology* **2015,** *49*, (4), 2171-2179.

35. Hug, S. J.; Leupin, O., Iron-Catalyzed Oxidation of Arsenic(III) by Oxygen and by Hydrogen Peroxide: pH-Dependent Formation of Oxidants in the Fenton Reaction. *Environmental Science & Technology* **2003,** *37*, (12), 2734-2742.
36. Manceau, A., The mechanism of anion adsorption on iron oxides: Evidence for the bonding of arsenate tetrahedra on free Fe(O, OH)6 edges. *Geochimica et Cosmochimica Acta* **1995,** *59*, (17), 3647-3653.
37. Liu, J.; Sheng, A.; Li, X.; Arai, Y.; Ding, Y.; Nie, M.; Yan, M.; Rosso, K. M., Understanding the Importance of Labile Fe(III) during Fe(II)-Catalyzed Transformation of Metastable Iron Oxyhydroxides. *Environmental Science & Technology* **2022,** *56*, (6), 3801–3811.
38. Roberts, L. C.; Hug, S. J.; Ruettimann, T.; Billah, M. M.; Khan, A. W.; Rahman, M. T., Arsenic Removal with Iron(II) and Iron(III) in Waters with High Silicate and Phosphate Concentrations. *Environmental Science & Technology* **2004,** *38*, (1), 307-315.
39. Raber, G.; Stock, N.; Hanel, P.; Murko, M.; Navratilova, J.; Francesconi, K. A., An improved HPLC–ICPMS method for determining inorganic arsenic in food: Application to rice, wheat and tuna fish. *Food Chemistry* **2012,** *134*, (1), 524-532.
40. Wielinski, J.; Marafatto, F. F.; Gogos, A.; Scheidegger, A.; Voegelin, A.; Müller, C. R.; Morgenroth, E.; Kaegi, R., Synchrotron hard X-ray chemical imaging of trace element speciation in heterogeneous samples: development of criteria for uncertainty analysis. *Journal of Analytical Atomic Spectrometry* **2020,** *35*, (3), 567-579.
41. Mangold, S.; Steininger, R.; Rolo, T. d. S.; Göttlicher, J., Full field spectroscopic imaging at the ANKA-XAS- and -SUL-X-Beamlines. *Journal of Physics: Conference Series* **2013,** *430*, 012130.
42. Mangold, S.; van de Kamp, T.; Steininger, R., New data evaluation procedure including advanced background subtraction for radiography using the example of insect mandibles. *Journal of Physics: Conference Series* **2016,** *712*, 012141.
43. Senn, A.-C.; Kaegi, R.; Hug, S. J.; Hering, J. G.; Mangold, S.; Voegelin, A., Composition and structure of Fe(III)-precipitates formed by Fe(II) oxidation in water at near-neutral pH: Interdependent effects of phosphate, silicate and Ca. *Geochimica et Cosmochimica Acta* **2015,** *162*, 220-246.
44. Al-Borno, A.; Tomson, M. B., The temperature dependence of the solubility product constant of vivianite. *Geochimica et Cosmochimica Acta* **1994,** *58*, (24), 5373-5378.
45. van Genuchten, C. M.; Behrends, T.; Stipp, S. L. S.; Dideriksen, K., Achieving arsenic concentrations of <1 µg/L by Fe(0) electrolysis: The exceptional performance of magnetite. *Water Research* **2020,** *168*, 115170.
46. Ravel, B.; Newville, M., ATHENA, ARTEMIS, HEPHAESTUS: data analysis for X-ray absorption spectroscopy using IFEFFIT. *Journal of Synchrotron Radiation* **2005,** *12*, (4), 537-541.
47. Freeze, R. A.; Cherry, J. A., *Groundwater*. Prentice-Hall, Inc.: Englewood Cliffs, New Jersey 07632, 1979.
